# Degradation of p47 by autophagy contributes to CADM1 overexpression in ATLL cells through the activation of NF-κB

**DOI:** 10.1038/s41598-019-39424-7

**Published:** 2019-03-05

**Authors:** Bidhan Sarkar, Ichiro Nishikata, Shingo Nakahata, Tomonaga Ichikawa, Toshiyuki Shiraga, Hasi Rani Saha, Masahiro Fujii, Yuetsu Tanaka, Kazuya Shimoda, Kazuhiro Morishita

**Affiliations:** 1 Division of Tumor and Cellular Biochemistry, Department of Medical Sciences, Faculty of Medicine, University of Miyazaki, Miyazaki, Japan; 2 Department of Foods and Human Nutrition, Faculty of Human Life Sciences, Notre Dame Seishin University, Okayama, Japan; 3 Division of Virology, Niigata University Graduate School of Medical and Dental Sciences, Niigata, Japan; 4 Department of Immunology, Graduate School of Medicine, University of the Ryukyus, Okinawa, Japan; 5 Division of Gastroenterology and Hematology, Department of Internal Medicine, Faculty of Medicine, University of Miyazaki, Miyazaki, Japan

## Abstract

Cell adhesion molecule 1 (CADM1), a member of the immunoglobulin superfamily, is identified as a novel cell surface marker for human T-cell leukemia virus (HTLV-1)-infected T cells. Adult T-cell leukemia/lymphoma (ATLL) is developed in HTLV-1-infected T-cells after a long infection period. To examine the mechanism of CADM1 overexpression in ATLL, we first identified that CADM1 is transcriptionally up-regulated by a transcriptional enhancer element through NF-κB signaling pathway. In HTLV-1-infected T-cells, CADM1 expression is dependent on HTLV-1/Tax through activation of canonical and non-canonical NF-κB; however, in ATLL cells with frequent loss of Tax expression, the activation of canonical NF-κB only enhances the CADM1 expression. Along with active mutations in signaling molecules under T-cell recepor (TCR) signaling, degradation of p47, a negative regulator of NF-κB, was essential for activation of canonical NF-κB through stabilization of NEMO (NF-κB essential modulator). The mechanism of p47 degradation is primarily dependent on activation of lysosomal-autophagy and the autophagy is activated in most of the HTLV-infected and ATLL cells, suggesting that the p47 degradation may be a first key molecular event during HTLV-1 infection to T-cells as a connector of two important signaling pathways, NF-κB and autophagy.

## Introduction

Adult T-cell leukemia/lymphoma (ATLL) is a malignancy of CD4^+^ T-cells associated with human T-cell leukemia virus type 1 (HTLV-1) infection. ATLL occurs after 40 to 50 years of latency in a small percentage (1–5%) of infected individuals. HTLV-1 is endemic in certain regions of the world, including southwestern Japan, the Caribbean islands, parts of South America, and Central Africa. An estimated over 20 million people worldwide are currently infected with HTLV-1. Although new therapeutic strategies such as hematopoietic stem cell transplantation or anti CCR4 antibodies are now being developed to treat ATLL, the overall prognosis of ATLL patients remains very poor^1^.

Cell adhesion molecule 1 (CADM1/TSLC1) is a cell adhesion molecule of the immunoglobulin superfamily that participates in cell-cell adhesion and transmembrane protein localization in epithelial cells. The *CADM1* gene was originally identified as a tumor suppressor gene in non-small cell lung cancer, and the loss of CADM1 expression is associated with a poor prognosis and metastasis in various types of solid cancers^2^. By contrast, CADM1 is highly expressed in ATLL cells, while CD4^+^ T-cells from healthy subjects do not express detectable CADM1^3^. The expression of CADM1 promotes the self-aggregation of ATLL cells and attachment of ATLL cells to endothelial cells^3^. Moreover, CADM1 expression enhances tumor growth and invasion of ATLL cells in a xenograft mouse model^4^. Because CADM1 is specifically and consistently expressed in ATLL cells^3,5^, CADM1 is considered not only the best cell surface marker but also an attractive molecular target for ATLL. On the other hand, how the *CADM1* gene is transcriptionally activated in ATLL cells remains debatable.

The expression of HTLV-1-encoded oncoprotein Tax has been shown to up-regulate CADM1 expression in various organs of *Tax*-transgenic mice and is partly involved in the nuclear factor-κB (NF-κB) signaling pathway^6^. Another study showed that Tax expression in the T-cell acute lymphoblastic leukemia (T-ALL) CEM cell line does not induce CADM1 expression, but it highly activates CADM1 expression in combination with the phorbol 12-myristate 13-acetate (PMA)/calcimycin (CAI) treatment through T-cell activation^7^. Because Tax expression is frequently lost at later stages of the development of ATLL^8^, Tax appears not to be the primary determinant for the overexpression of CADM1 in ATLL cells.

The NF-κB family of transcription factors plays an important role in immune and inflammatory responses by inducing the expression of pro-inflammatory cytokines. The canonical NF-κB pathway is activated by the stimulation of proinflammatory receptors, such as tumor necrosis factor-α (TNF-α), and the non-canonical NF-κB pathway is activated by members of the TNF-receptor superfamily, including the lymphotoxin β receptor^9^. In HTLV-1-infected T-cells, Tax was reported to activate the canonical and noncanonical NF-κB signaling pathways through the association with various signaling proteins, including IκB kinase (IKK) and NF-κB transcriptional complex^10^. In addition, activation of the NF-κB pathway is also observed in ATLL cells with low levels of Tax expression through mechanisms involving the down-regulation of miR-31 and frequent somatic alterations in the TCR-NF-κB pathway^11,12^. Because the NF-κB pathway plays important roles in the survival and proliferation of HTLV-1-infected T-cells and ATLL cells, there must be some additional mechanism to ensure the sustained activation of the NF-κB pathway in ATLL cells.

Along with activating alterations in the NF-κB pathway, the deregulation of negative regulators of the NF-κB pathway could be involved in the constitutive activation of NF-κB in ATLL. In the NF-κB pathway, three negative feedback regulators, TNF-α-induced protein 3 (TNFAIP3, A20), Cylindromatosis (CYLD), and NSFL1 cofactor (p47), have been identified to regulate NF-κB activation after signal stimulation. Both A20 and CYLD cleave K63-linked polyubiquitin chains from signaling components involved in NF-κB activation, such as TNF receptor associated factor 6 (TRAF6) and NEMO/IKKγ^13^. The p97/NSFL1 cofactor p47, a major adaptor molecule of the cytosolic AAA ATPase p97 that participates in the biogenesis of endoplasmic reticulum and Golgi apparatus^14^, has recently been reported to bind to NEMO with the Lys63-linked and linear polyubiquitin chains, leading to the lysosome-dependent NEMO protein degradation and suppression of IKK activation^15^. Although the down-regulation of A20 or CYLD has been reported in certain malignancies^16,17^, it has not been clarified whether the deregulation of expression of these negative regulators is involved in the persistent activation of the NF-κB pathway in ATLL.

In this study, we first precisely examined the promoter activity of *CADM1* in ATLL cells and found an enhancer element for the CADM1 expression at the *CADM1* promoter region in ATLL cells that contain the NF-κB-binding sequence. In HTLV-1-infected T-cell lines expressing Tax, Tax directly activated both the canonical and non-canonical NF-κB pathways; however, in ATLL cell lines with low Tax expression, only the canonical NF-κB pathway was activated by factor(s) other than Tax. Because the loss of p47 protein expression was found along with increased NEMO protein levels in most ATLL-related cell lines and primary ATLL cells, the down-regulation of p47 protein was a candidate for activating CADM1 expression in ATLL cells. Indeed, ectopic expression of p47 in ATLL cell lines induced NEMO degradation and inhibition of NF-κB activation with retardation of cell growth, while the knock-down of p47 in HTLV-1-negative T-ALL cell lines induced NF-κB activation and acceleration of cell growth under TNF-α stimulation. Furthermore, the down-regulation of p47 in ATLL-related cell lines is caused by the activation of the autophagy degradation pathway. Thus, the down-regulation of p47 is an important mechanism for the constitutive activation of the NF-κB pathway in ATLL cells along with HTLV-1/Tax, and CADM1 is one of the important target genes for NF-κB activation during leukemogenesis after HTLV-1 infection, which may render CADM1 as a specific cell surface marker for HTLV-1-infected T-cells.

## Materials and Methods

### Patient samples

Peripheral blood samples were collected from the patients at the time of hospital admission before the chemotherapy started. Blood samples were also obtained from healthy volunteers as controls. Blood samples were collected at the Department of Medical Sciences, Faculty of Medicine, University of Miyazaki, as a collaboration with the Miyazaki University Hospital. The diagnosis of ATLL was based on clinical features, hematological characteristics, the presence of anti-HTLV-1 antibodies, and clonal integration of the HTLV-1 provirus. The study was performed in accordance with the Declaration of Helsinki, the Ethical Guidelines for Medical and Health Research Involving Human Subjects, and the Ethics Guidelines for Human Genomic/Genetic Analysis Research. Written informed consent was obtained from all participants in this study. The study was approved by the Institutional Review Board at Faculty of Medicine, University of Miyazaki. Peripheral blood mononuclear cells (PBMCs) were isolated by density gradient centrifugation using Histopaque (Sigma-Aldrich, St. Louis, MO, USA). The method for the separation of ATLL cells from PBMCs has been described elsewhere^5^. CD4^+^ T-cells were purified from the PBMCs of healthy volunteers using anti-CD4 magnetic beads (Miltenyi Biotec, Auburn, CA, USA).

### Cell lines

T-ALL cell lines (Jurkat and MOLT4), HTLV-1-infected T-cell lines (HUT102, MT2, MT4, and SLB1), and ATLL-derived cell lines (ATL3I, ATL5S, ED, KOB, S1T, and KK1) were maintained in RPMI 1640 medium supplemented with 10% fetal bovine serum (FBS) and 50 µg/ml of penicillin/streptomycin in a 5% CO_2_ chamber at 37 °C. The interleukin-2 (IL-2)-dependent ATLL-derived cell lines KK1 and KOB were maintained in complete RPMI 1640 medium supplemented with 0.75 μg/mL of recombinant human IL-2 (Peprotech, Rocky Hill, NJ, USA). The human embryonic kidney (HEK) 293 T cell line and mouse embryonic fibroblasts (MEFs) were cultured in Dulbecco’s modified Eagle’s medium (DMEM, Wako, Osaka, Japan) supplemented with 10% FBS and 50 µg/ml of penicillin/streptomycin. Tax-immortalized human T-lymphocytes^18,19^ (kind gifts from Dr. M. Hijikata, Kyoto University, Japan) were maintained in AIM-V medium (Thermo Fisher Scientific, Waltham, MA, USA) supplemented with 20% FBS, 10 μM 2-mercaptoethanol (Thermo Fisher Scientific) and 0.75 μg/mL recombinant human IL2. JPX-9 is a derivative of Jurkat cells stably transfected by the *Tax* gene under the control of a ZnCl_2_-inducible metallothionein promoter^20^ and was kindly provided by Dr. K Sugamura (Tohoku University School of Medicine, Japan). Jurkat and MOLT4 were obtained from the Fujisaki Cell Center, Hayashibara Biochemical Laboratories (Okayama, Japan). HUT102, MT2, MT4, and SLB1 were kindly provided by Dr. H. Iha (Oita University, Japan). ST1, KOB, and KK1 were kindly provided by Dr. Y. Yamada (Nagasaki University, Japan). S1T and Su9T were a kind gift from Dr. N. Arima (Kagoshima University, Japan). ED was kindly provided by Dr. M. Maeda (Kyoto University, Japan). ATL3I and ATL5S were kindly provided by Dr. T. Sugimura (National Cancer Center, Japan). HEK 293 T cells were obtained from the RIKEN Bioresource Center (Tsukuba, Japan).

### Antibodies and reagents

Anti-NSFL1C p47 mouse polyclonal antibody (Abnova, Taipei City, Taiwan) and anti-A20/TNFAIP3 mouse monoclonal antibody were purchased from Novus Biologicals (LLC 8100 South Park Way, USA). Anti-IκBα (C-21), anti-NEMO (FL-419), and anti-NIK (H-248) rabbit polyclonal antibodies were from Santa Cruz Biotechnology (Santa Cruz, CA, USA). Anti-p-IκBα (Ser32/36) mouse monoclonal antibody, anti-CYLD (D1A10) rabbit monoclonal antibody, and anti-ATG5 (D5F5U) rabbit monoclonal antibody were from Cell Signaling Technology (Danvers, MA, USA), and anti-β-actin mouse monoclonal antibody was from Sigma-Aldrich. Anti-LC3B rabbit polyclonal antibody was purchased from Novus Biological. Anti-Tax (MI73^21^ and TAXY-7^22^) mouse monoclonal antibodies were a kind gift from Dr. M. Matsuoka (Kyoto University, Japan) and Dr. Y. Tanaka (University of Ryukyus, Japan), respectively. Horseradish peroxidase-conjugated anti-mouse and anti-rabbit IgG secondary antibodies were purchased from Dako (Carpinteria, CA, USA). Hilymax transfection reagent was purchased from DOJINDO (Kumamoto, Japan). E64D was obtained from Wako, and pepstatin A was from Peptide Institute (Osaka, Japan). MG132 was obtained from Life Sensors (Malvern, PA, USA), and TNF-α was obtained from Wako.

### Plasmids

A 3.4-kb fragment of the human *CADM1* promoter with the transcription initiation site was isolated by polymerase chain reaction (PCR) amplification. The PCR product was directly subcloned into the pGL3B firefly luciferase reporter vector (Promega, Madison, WI, USA). A series of 5′ deletion mutants of the *CADM1* promoter constructs were generated from pGL4/4.2 K by PCR. The FLAG-p47 construct was generated by PCR using the p47/pRK5^15^ (a kind gift from J. Inoue, University of Tokyo, Japan) as the template and subcloned into p3XFLAG-myc-CMV-26 (Sigma-Aldrich). The pNF-κB-Luc and pRL-TK were purchased from Agilent Technologies (Santa Clara, CA, USA) and Promega, respectively. To construct the short hairpin RNA (shRNA) expression vector targeting Tax, p47, or ATG5, the following oligonucleotides were annealed and inserted into the RNAi-Ready pSIREN-RetroQ-ZsGreen vector (Clontech, Palo Alto, USA): for Tax, 5′ GCAGATGACAATGACCATGA 3′; for p47, 5′ GAGTGGATTCAGCCTGGAT 3′; for ATG5, 5′ GCCTGAACAGAATCATCCTTAA 3′. A negative control shRNA targeting luciferase was obtained from Clontech.

### Reverse transcription (RT)-PCR analysis

Total RNA was extracted from cells using TRIzol RNA Isolation Reagents (Thermo Fisher Scientific), and 1 μg of total RNA was used to synthesize first-strand cDNA using the PrimeScript 1st strand cDNA Synthesis Kit (Takara Bio, Otsu, Japan) following the manufacturer’s recommendations. Semi-quantitative RT-PCR was performed using Ex Taq (Takara Bio). Real-time PCR was performed using GeneAce SYBR qPCR Mix α (Wako) and the Applied Biosystems StepOne System (Thermo Fisher Scientific). The data were normalized to β-actin mRNA levels and were expressed relative to the value in the MOLT4 cell line or the mean value from healthy volunteers as the means ± the standard deviation from triplicates. The experiments were repeated three times, and Student’s *t* test was used for statistical analysis. The primer sequences are listed in Supplementary Table S1.

### Western blot analysis

Cells were washed with phosphate-buffered saline (PBS) and were lysed in NP-40 lysis buffer (50 mM of Tris-HCl, pH 8.0, 150 mM of NaCl, 5 mM of EDTA, 1% NP-40) supplemented with protease inhibitor cocktail (Sigma-Aldrich) and phosphatase inhibitors (PhosStop, Roche, Indianapolis, IN, USA). After centrifugation, the lysates were boiled for 5 min in 1 × SDS sample buffer (62.5 mM of Tris–HCl, pH 6.8, 2% SDS, 25% glycerol, 5% β-mercaptoethanol and 0.01% bromophenol blue), separated on 10% SDS-polyacrylamide gels, and transferred to polyvinylidene fluoride (PVDF) membranes (Immobilon-P, Millipore, Billerica, MA, USA). The membranes were blocked with 5% non-fat dried milk or 1% bovine serum albumin (BSA) in TBS-T (10 mM of Tris-HCl, pH 7.4, 100 mM of NaCl, and 0.1% Tween 20) or Blocking One (Nacalai Tesque, Kyoto, Japan) for 1 h at room temperature and then were incubated with the primary antibodies diluted in blocking buffer overnight at 4 °C. After washing 3 times with TBS-T, the membranes were incubated with horseradish peroxidase-conjugated secondary antibodies diluted in blocking buffer for 1 h and washed again, and then the signals were visualized on LAS-3000 (Fuji-film, Tokyo, Japan) using the EzWestLumi plus Western blotting substrate (ATTO, Tokyo, Japan).

### Luciferase reporter assays

Cells were transfected with the firefly luciferase reporter plasmids carrying the human *CADM1* promoter or three repeated NF-κB-responsive elements along with the internal control Renilla luciferase plasmid pRL-TK using the Hilymax transfection reagent (Dojindo) or by electroporation using the Gene Pulser system (Bio-Rad, Hercules, CA, USA). At 24 h post-transfection, luciferase activity was measured with the Dual-Luciferase-Assay kit (Promega) according to the manufacturer’s instructions with a luminometer (Turner Designs Luminometer, Model TD-20/20; Promega). For JPX-9 cells, at 24 h post-transfection, cells were treated with ZnCl_2_ for 12 h, and luciferase activity was measured. For shRNA transfection, cells were transfected with the plasmids encoding shRNA against p47 or luciferase as a control by electroporation using the Gene Pulser system (Bio-Rad). At 48 h post-transfection, cells were treated with TNF-α for 12 h, and luciferase activity was measured.

### Cell proliferation assay

Cells were seeded into 25-cm^2^ T-flasks at a density of 1 × 10^5^ cells per ml in complete RPMI 1640 medium in a 5% CO_2_ chamber at 37 °C. The number of viable cells was determined every 24 h for 6 days by the Trypan blue exclusion method.

### Electrophoretic mobility shift assay (EMSA)

To prepare the nuclear extracts, cells were extracted in buffer containing 10 mM of HEPES-KOH, pH 7.8, 10 mM of KCl, 0.1 mM of EDTA, 0.1% NP-40, 1 mM of dithiothreitol, and protease inhibitor cocktail (Sigma-Aldrich). The nuclear pellet was resuspended on ice in nuclear extraction buffer (50 mM of HEPES-KOH, pH 7.8, 420 mM of KCl, 0.1 mM of EDTA, 5 mM of MgCl_2_, 2% glycerol, 1 mM of dithiothreitol, and protease inhibitor cocktail). The protein concentrations were determined using the BCA Protein Assay (Pierce, Rockford, IL). The double-stranded DNA probe was labeled with [α-^32^P]dCTP using the Klenow enzymes. The binding reaction was performed at room temperature for 15 min in binding buffer (10 mM of HEPES-KOH, pH 7.8, 50 mM of KCl, 1 mM of MgCl_2_, 2 µM of ZnSO_4_, 1 mM of EDTA, 10% glycerol, 1 mM of dithiothreitol, and protease inhibitor cocktail) containing approximately 50,000 c.p.m. of the labeled probe, 2 µg of poly (dI-dC), and the nuclear extracts. Unlabeled oligonucleotides were added in a 200-fold molar excess as a competitor. The DNA-protein complexes were resolved in a native 4% acrylamide gel.

### Chromatin immunoprecipitation (ChIP) assay

ChIP assays were performed using standard procedures. After transfection of the pGL3B-729 promoter constructs by electroporation, cells were cross-linked with 1% formaldehyde and sonicated with a Sonifier 450 (Branson). After the lysates were immunoprecipitated with antibodies against Tax (MI73), p50, p65, p52, or p68 or control IgG, immunoprecipitated chromatin was reverse cross-linked at 65 °C overnight, digested with RNase A and proteinase K, and then purified. Purified DNA fragments were amplified using primers for the specific NF-κB-binding site at the promoter, forward 5′-TATTAAAACCAGGGAGGAGACCC-3′ and reverse 5′-GCTTTGCACGTCCGGCGGAG-3′.

### Statistical analysis

Statistical significance was analyzed using Student’s *t*-test from the GraphPad Prism software, version 5.00 for Windows (GraphPad Software Inc., San Diego, CA, USA). Values of *P* < 0.05 were considered significant.

## Results

### CADM1 expression is specifically up-regulated through the sequence from −729 to −680 of the *CADM1* promoter in ATLL

**Figure 1:**
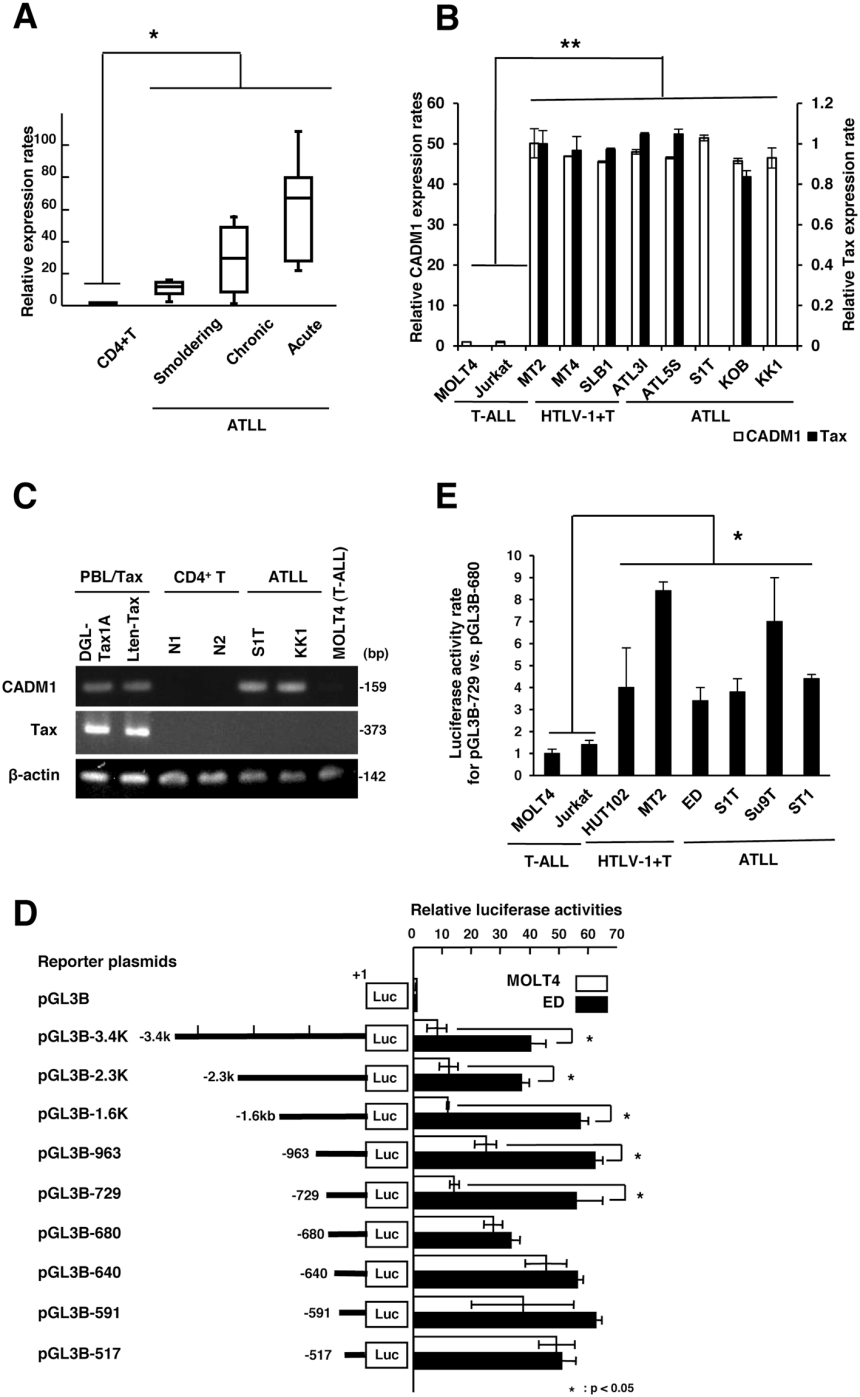
Activation of *CADM1* gene expression and identification of the transcriptional activator region in the *CADM1* promoter in ATLL. (**A**) Real-time RT-PCR analysis for *CADM1* in CD4^+^ T-cells from three healthy volunteers and ATLL cells from patients with three different subtypes of ATLL (each six cases of the smoldering, chronic and acute types)^5^. Box plots of the relative *CADM1* mRNA expression are shown. *P < 0.05 (Student’s *t* test). (**B**) Real-time RT-PCR analysis for *CADM1* and *Tax* in two HTLV-1-negative T-ALL cell lines (T-ALL), three HTLV-1-infected T-cell lines (HTLV-1 + T), and six ATLL cell lines (ATLL) as indicated in the figure. The data represent the means ± S.D. of triplicate determinations and are presented relative to the MOLT4 (for CADM1) and MT2 (for Tax) cell lines (set as 1). **P < 0.01 (Mann-Whitney *U* test). (**C**) Semiquantitative RT-PCR analysis for *CADM1* and *Tax* in two samples of Tax-immortalized T-cells (PBL/Tax), two samples of CD4^+^ T-cells from healthy volunteers, two ATLL cell lines (S1T and KK1), and one T-ALL cell line (MOLT4). β-actin was used as a loading control. (**D**) Luciferase activity of the serial *CADM1* promoter deletion mutants from −3,400 to −517 bp, as indicated in the figure, was determined by transfection into the ED/ATLL cell line (black box) or MOLT4/T-ALL cell line (white box). The data represent the means ± S.D. of triplicate determinations and are presented relative to pGL3-basic activity. *P < 0.05 (Student’s *t* test). (**E**) Luciferase activity of the pGL3B-729 and pGL3B-680 constructs in two T-ALL (Jurkat and MOLT4; white box) and six ATLL-related cell lines (HUT102, MT2, ED, S1T, Su9T, and ST1; black box). The data represent the fold change in the luciferase activity of pGL3B-729 over that of pGL3B-680 and are presented relative to the MOLT4 cell line. *P < 0.05 (Mann-Whitney *U* test).

Because we found specific expression of CADM1 in ATLL cells, we initially determined the expression of CADM1 in leukemia samples from patients with various types of ATLL and in ATLL-related cell lines by quantitative PCR. As shown in Fig. 1A, *CADM1* expression was significantly increased with disease progression through smoldering-, chronic- and acute-type ATLL. Moreover, approximately 50-fold up-regulation of *CADM1* was observed in the HTLV-1-infected and ATLL-derived cell lines compared with the HTLV-1-negative T-ALL cell lines (Fig. 1B). Because these HTLV-1-infected T-cell lines preserve Tax expression (Fig. 1B), we next determined whether Tax induces the expression of *CADM1*. We found that peripheral blood lymphocytes immortalized by Tax expressed CADM1, which was comparable to the levels in ATLL-derived cell lines (Fig. 1C), indicating that CADM1 is overexpressed in both Tax-positive HTLV-1-infected T-cells and Tax-negative ATLL cells.

Based on the expression study of *CADM1* in ATLL cells, we next determined the promoter activity of *CADM1* in ATLL cells after isolation and subcloning of the 3.4-kb of the 5′ region of *CADM1* into the pGL3B luciferase basic vector. After subcloning 3.4 kb of the 5′ region of CADM1, various deletion mutant clones were established as shown in Fig. 1D. After transfection of these luciferase-reporter plasmids to ED/ATLL cells or MOLT4/T-ALL cells as a control, the luciferase activity of each promoter was compared between ED and MOLT4 cells. The luciferase activities of pGL3B-3.4 K to pGL3B-729 in ED cells were significantly higher than those in MOLT4 cells (Fig. 1D). Interestingly, the enhancement of the *CADM1* promoter in ED cells was no longer detected in the promoter from −680 to −517. To confirm the results, we used two control cell lines (MOLT4/T-ALL and Jurkat/T-ALL) and two HTLV-1-infected cell lines (HUT102 and MT2), as well as four ATLL cell lines (ED, S1T, Su9T, and ST1), and determined the transcriptional activity of the −729 relative to the −680 of the *CADM1* promoter region after transfection of the pGL3B/Mock, pGL3B-729 or pGL3B-680 plasmid. Although the transcriptional activity of pGL3B-729 in the two control cell lines was less than that of pGL3B-680, the two HTLV-1-infected cell lines and four ATLL cell lines showed higher transcriptional activities of pGL3B-729 than those of pGL3B-680 with a significant difference (Figs 1E and S1). Therefore, it was suggested that a transcriptional enhancer element of *CADM1* in HTLV-1-infected and ATLL cell lines exists in the promoter region of *CADM1* between −729 and −680.

### The NF-κB binding site is primarily responsible for the transcriptional activation of *CADM1* in ATLL

**Figure 2:**
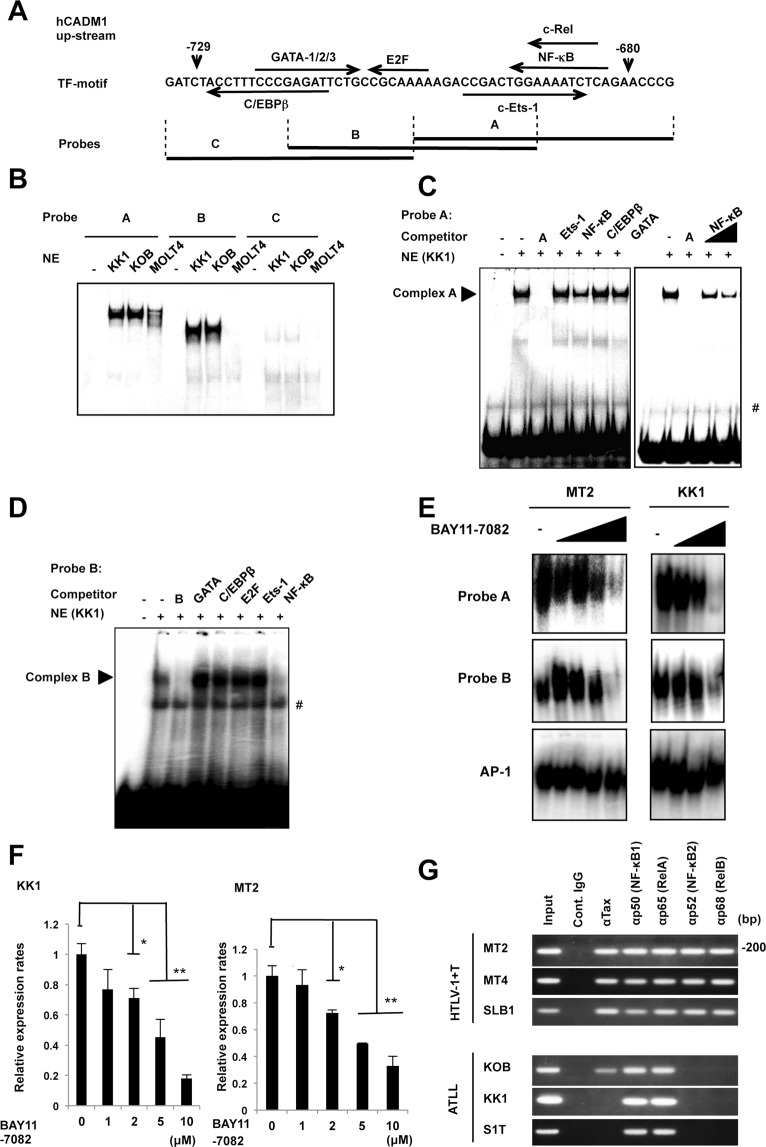
NF-κB transcription factors function in the transcriptional activation of *CADM1* in ATLL cells. (**A**) Schematic representation of the sequence from −729 to −680 in the *CADM1* promoter and potential transcription factor-binding sites. The position of each probe fragment (**A**–**C**) used in the EMSA is also shown. (**B**) EMSA performed with fragment (**A**–**C**) probe and nuclear extracts (NE) from two ATLL cell lines (KK1 and KOB) and the MOLT4/T-ALL cell line. A reaction using a probe alone with no nuclear extract was included as a negative control (−). (**C**,**D**) Competitive EMSA performed with fragment **A** or **B** probe and nuclear extracts from the KK1/ATLL cell line in the presence of a 200-fold excess of the indicated competitor DNA fragments (**A**, **B**, or each binding sequence of transcription factors). A specific shifted band is indicated as Complex **A** or **B**, and # indicates the background signal. (**E**) EMSA performed with the fragment A or B probe, or AP-1 probe as a control, and nuclear extracts from the two ATLL cell lines (MT2 and KK1) treated or untreated with various concentrations (0, 1, 3, 5 or 10 μM) of the NF-κB inhibitor BAY11-7082. (**F**) Real-time RT-PCR analysis for CADM1 in two ATLL-related cell lines (KK1 and MT2) 12 h after treatment with various concentrations of the NF-κB inhibitor Bay11-7082. The data represent the means ± S.D. of triplicate determinations and are presented relative to the untreated control (set as 1). *P < 0.05, **P < 0.01 (Student’s *t* test). (**G**) ChIP-PCR performed on HTLV-1-infected T-cell lines (MT2, MT4, and SLB1) and ATLL cell lines (KOB, KK1, and S1T) with each indicated antibody and amplified primers specific for the NF-κB-like site in the *CADM1* promoter. One-hundredth of the total DNA from nuclear extracts is the positive control (Input), and precipitated DNA with non-immune immunoglobulin of the same isotype (cont. IgG) was used as a negative control.

Because the sequence between −729 and −680 in the *CADM1* promoter is important for the transcriptional activation of *CADM1* in ATLL cells, we next searched for transcription factor-binding sites in this region using the TFSEARCH program^23^. As shown in Fig. 2A, we found several potential binding motifs for the GATA family, c-ETS-1, C/EBPβ, E2F, NF-κB and c-Rel, as indicated by the arrow, in the −729 to −680 in the *CADM1* promoter. We performed electrophoretic mobility shift assay (EMSA) with three DNA fragments ~30 nucleotides in length (A to C) covering the region between −729 and −680 (Fig. 2A), nuclear extracts from MOLT4/T-ALL as a control and two ATLL cell lines (KK1 and KOB). In probes A and B, the shifted bands corresponding to the DNA-protein complex were clearly detected in nuclear lysates from two ATLL cell lines but were not or weakly detected in the no lysate control or lysate from MOLT4 cells (Fig. 2B), suggesting that the sequence covered by the probes A and B might be responsible for the transcriptional activation of the *CADM1* promoter in ATLL cells. Next, we determined which of the potential transcription factor binding elements are involved in the DNA-protein complex in the *CADM1* promoter by EMSA assay with each excess cold competitor, Ets-1, NF-κB, C/EBPβ, GATA, and fragments A and B. In probe A, the intensity of the shifted band was completely abrogated by the addition of an excess amount of cold fragment A and was weakly inhibited by the cold NF-κB consensus sequence (Fig. 2C). Additionally, the band intensity of the DNA-protein complex was dose-dependently inhibited by the cold NF-κB consensus sequence, although no change in the intensity of the DNA-protein-binding was observed with other cold competitor fragments (Fig. 2C). Moreover, in EMSA with probe B, the band intensity was clearly reduced with cold competitors of the NF-κB consensus sequence or fragment B (Fig. 2D). These results suggest the NF-κB-like binding sequence in the −729 to −680 region of the *CADM1* promoter in probes A and B is important for its enhanced transcriptional activation in ATLL cells.

To determine whether the NF-κB transcription factors are responsible for the enhanced expression of *CADM1* in ATLL cells, we treated the MT2/HTLV-1-infected T-cell line and KK1/ATLL-derived cell line with the NF-κB inhibitor BAY11-7082 and analyzed for EMSA using probe A or B and the expression of *CADM1* by real-time RT-PCR analysis. In EMSA using nuclear extracts from MT2 and KK1 cell lines, treatment with BAY11-7082 dose-dependently inhibited the shifted bands of probes A and B and had no effect on the shifted band of AP-1 as a control (Fig. 2E). In addition, the expression of *CADM1* was suppressed by treatment with BAY11-7082 in a dose-dependent manner (Fig. 2F), and BAY11-7082 treatment also significantly inhibited the expression of known NF-κB target genes, *IL-6* and *INOS* (Fig. S2A). Therefore, *CADM1* expression is dependent on the activation of the NF-κB signaling pathway through the activation of NF-κB-like binding sequences at the *CADM1* promoter in ATLL cells.

Therefore, to determine the binding of NF-κB transcriptional factors to the NF-κB-like binding sequences in the *CADM1* promoter, ChIP-PCR was performed with various antibodies against different subunits of the NF-κB transcription factor complexes or HTLV-1/Tax for immunoprecipitation and primers targeting to the DNA fragment containing NF-κB regions for the amplification of DNA in the immunoprecipitated protein-DNA complex. Three HTLV-1-infected cell lines (MT2, MT4, and SLB) with high Tax expression and three ATLL-derived cell lines (KOB, KK1, and S1T) with low Tax expression were used for this experiment. In three HTLV-1-infected cell lines, the transcription factors p50/p65 complex of the canonical NF-κB pathway and p52/ RelB complex of the non-canonical NF-κB pathway with Tax bound to the NF-κB-like binding sequences in the *CADM1* promoter (Fig. 2G). Moreover, in three ATLL cell lines with very low Tax expression, the p50/p65 complex bound to the *CADM1* promoter, while the p52/RelB complex did not (Fig. 2G). Therefore, although Tax enhances *CADM1* transcription in association with p50/p65 and p52/p68 in HTLV-1-infected T cells, an additional activation mechanism for the canonical NF-κB pathway may exist in ATLL cells with low Tax expression (Fig. S2B).

### Tax expression induces the transcriptional activation of *CADM1* cooperatively with the NF-κB signaling pathway in HTLV-1-infected T cells

**Figure 3:**
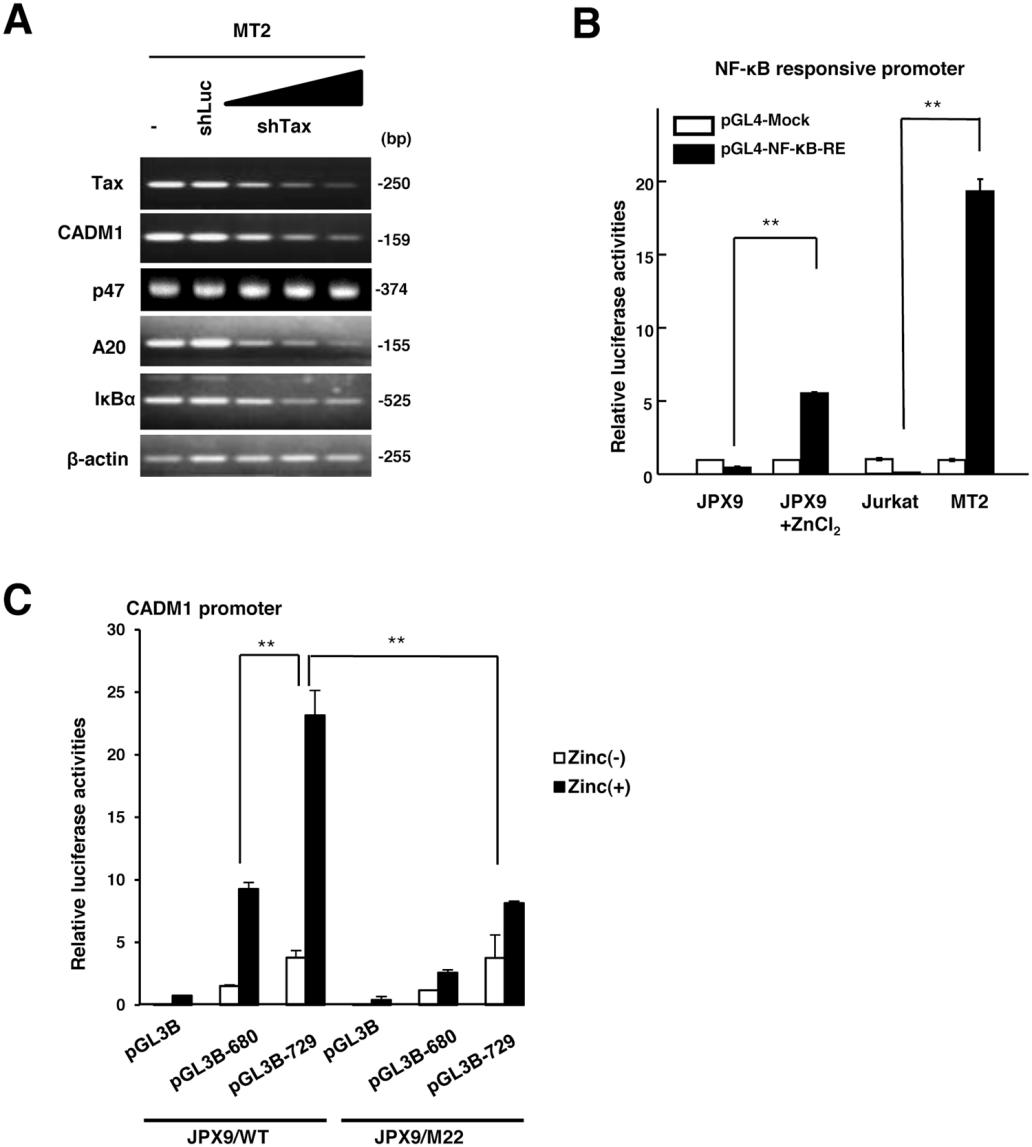
HTLV-1/Tax-dependent activation of *CADM1* expression via the NF-κB pathway. (**A**) Semiquantitative RT-PCR analysis was performed for Tax, CADM1, p47, A20, IκBα in the HTLV-1-infected T-cell line MT2 after transfection with various concentrations of shRNA-expression vector for Tax (shTax) or a shRNA expression vector for luciferase (shLuc) as a control. β-actin was used as a loading control. (**B**) Luciferase activity of the NF-κB-responsive luciferase reporter vector (pGL4-NF-κB-RE) or mock vector (pGL4-Mock) was determined in Tax-inducible JPX-9 cells with or without 120 µM of ZnCl_2_ treatment_,_ MT2, and Jurkat cells. The data represent the means ± S.D. of triplicate determinations and are presented relative to the luciferase activity of JPX-9 cells transfected with pGL4-Mock. **P < 0.01 (Student’s *t* test). (**C**) Luciferase activity of the pGL3B-729 or pGL3B-680 construct was determined in the JPX-9 or NF-κB-mutated JPX-9/M22 cells with or without 120 µM ZnCl_2_ treatment. The data represent the means ± S.D. of triplicate determinations and are presented relative to the JPX-9 cells transfected with pGL3B/Mock without ZnCl_2_ treatment. **P < 0.01 (Student’s *t* test).

Given that Tax-immortalized T-cells express high levels of CADM1, we next determined whether the transcriptional activation of *CADM1* by Tax expression is dependent on the activation of the NF-κB signaling pathway. We down-regulated Tax expression in MT2 cells by transfecting the increased amounts of a Tax shRNA vector and found that the expression level of CADM1 mRNA, as well as those of the NF-κB -responsive genes *A20* and *IκBα*, was decreased with Tax down-regulation in a dose-dependent manner (Fig. 3A). On the other hand, the induction of Tax expression by treatment with zinc chloride activated the NF-κB reporters with NF-κB response elements as well as the *CADM1* promoter in the Tax-inducible Jurkat cell line JPX9 (Fig. 3B,C), and this induction of the *CADM1* promoter was severely inhibited in the JPX9/M22 cells, which contain the Tax mutant defective in NF-κB activation^24^ (Fig. 3C). These results suggest that Tax can activate the transcription of *CADM1* via the activation of the NF-κB pathway in HTLV-1-infected T-cells.

### Down-regulation of the NF-κB negative regulator p47 in most of the ATLL-related cell lines

**Figure 4:**
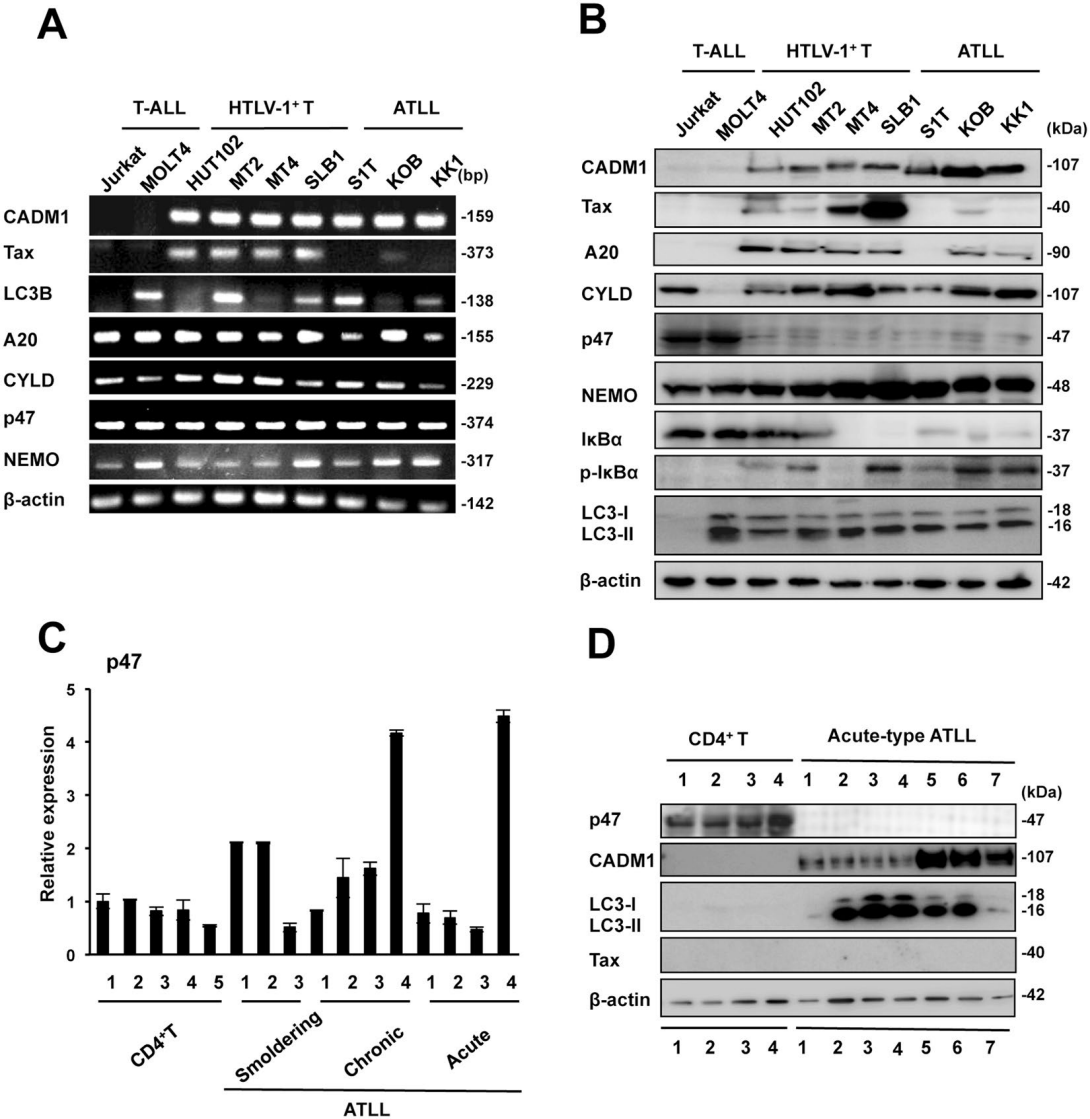
p47 is posttranslationally down-regulated in HTLV-1-infected and ATLL cell lines. (**A**) Semiquantitative RT-PCR analysis for *CADM1, Tax, LC3B, A20, CYLD, p47* and *NEMO* was performed in two T-ALL cell lines (Jurkat and MOLT4), three HTLV-1-infected T-cell lines (HUT102, MT2, and MT4), and three ATLL cell lines (S1T, KOB, and KK1). β-actin was used as a loading control. (**B**) Immunoblot analysis of CADM1, Tax, and the indicated NF-κB and autophagy signaling proteins was performed in the same series of cell lines used in Fig. 4A. β-actin was used as a loading control. The cropped gels/blots are used in the figure, and the full-length gels/blots are presented in Supplementary Fig. S7. (**C**) Real-time RT-PCR analysis for *p47* in CD4^+^ T cells from five healthy volunteers and ATLL cells from three smoldering-type, four chronic-type, and four acute-type ATLL patients. The data represent the means ± S.D. of triplicate determinations and are presented relative to the p47 expression level in control CD4^+^ T-cells (lane 1). (**D**) Immunoblot analysis of p47, CADM1, Tax, and LC3B was performed in four CD4^+^ T-cells from healthy volunteers and ATLL cells from eight acute-type ATLL patients. β-actin was used as a loading control. The cropped gels/blots are used in the figure, and the full-length gels/blots are presented in Supplementary Fig. S7.

One of the activation mechanisms of the canonical NF-κB pathway in the advanced stages of ATLL is derived from the accumulated activating mutations in the NF-κB pathways^11^ and others from the inactivation of negative regulators of the canonical NF-κB pathway, A20, CYLD, and p47^6,15,25^_._ In this study, we focused on the association between the levels of expression of these negative regulators and CADM1 expression in the activation of canonical NF-κB pathways. We initially performed semiquantitative RT-PCR analyses with various types of leukemia cell lines. The mRNA expression of *A20*, *CYLD*, and *p47* was not significantly different between the HTLV-1-negative T-ALL cell lines and HTLV-1-related cell lines (Fig. 4A). On the other hand, in the immunoblot analysis, p47 expression was significantly decreased in all the HTLV-1-related cell lines compared with the T-ALL cell lines, although the expression levels of A20 and CYLD were variable among the HTLV-1-related cell lines (Fig. 4B). Additionally, along with p47 down-regulation, we observed high expression of CADM1 and enhanced NEMO with increased degradation of total IκBα and increased phosphorylated-IκBα in most of the HTLV-1-related cell lines, resulting in enhanced activation of the canonical NF-κB signaling pathway (Figs 4B and S3). To confirm the results, we next analyzed the levels of p47 expression in primary leukemic cells from various types of ATLL patients with CD4^+^ T-lymphocytes from healthy volunteers as controls by quantitative RT-PCR and immunoblot analyses. The expression levels of p47 in leukemic cells from acute-type ATLL patients were comparable or higher than those in CD4^+^ T lymphocytes (Fig. 4C); however, the p47 protein levels were drastically decreased with the converse up-regulation of CADM1 in leukemia cells from all ATLL patients compared with the CD4^+^ T lymphocytes (Fig. 4D). These results suggest that the down-regulation of p47 is likely involved in the constitutive activation of the canonical NF-κB pathway through enhanced stabilization of NEMO, resulting in the up-regulation of CADM1 expression in ATLL.

### CADM1 is negatively regulated in T-ALL and ATLL cells by p47 through the activity of the canonical NF-κB pathway

**Figure 5:**
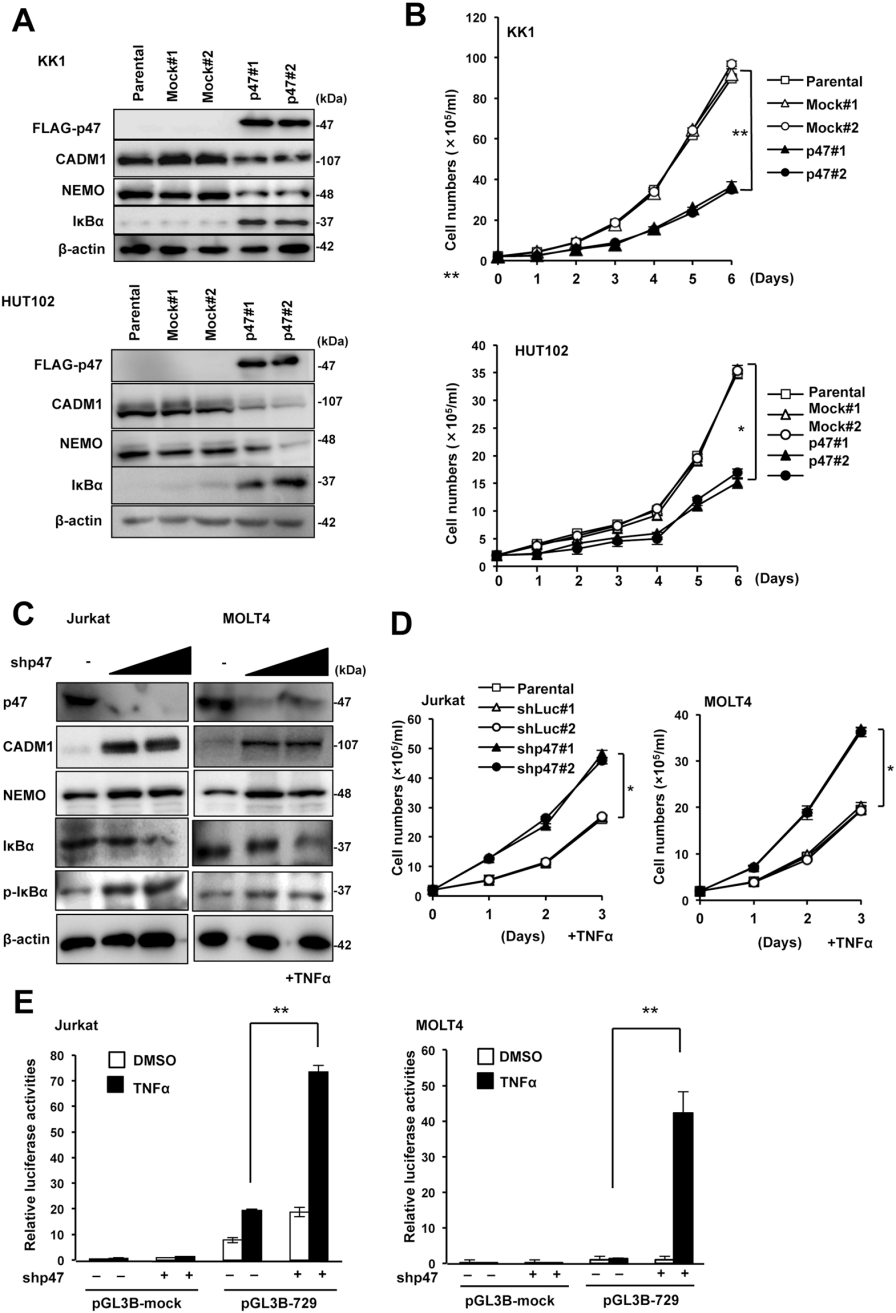
Down-regulation of p47 contributes to the activation of the canonical NF-κB pathway with enhanced expression of CADM1 in ATLL cells. (**A**) Immunoblot analysis of p47, CADM1, NEMO, and IκBα in KK1 or HUT102 cells was performed after transfection of a p47 expression or mock vector. Duplicated experiments were performed (#1 and #2), and β-actin was used as a loading control. The cropped gels/blots are used in the figure, and the full-length gels/blots are presented in Supplementary Fig. S7. (**B**) The cell proliferation of KK1 and HUT102 cells transfected with a p47 expression or mock vector was determined under the same condition as that in (**A**). The data represent the means ± S.D. of triplicate determinations. *P < 0.05, **P < 0.01 (Student’s *t* test). (**C**) Immunoblot analysis of p47, CADM1, NEMO, IκBα, and p-IκBα (Ser32/36) in Jurkat or MOLT4 cells was performed after transfection with an increasing dose of a p47 shRNA expression vector (0, 1, and 3 µg) under TNF-α treatment. β-actin was used as a loading control. The cropped gels/blots are used in the figure, and the full-length gels/blots are presented in Supplementary Fig. S7. (**D**) Cell proliferation was determined in Jurkat and MOLT4 cells under TNF-α treatment and the same condition as that in (**C**). Duplicated experiments were performed (#1 and #2). The data represent the means ± S.D. of triplicate determinations. *P < 0.05 (Student’s *t* test). (**E**) Luciferase activity was determined in Jurkat and MOLT4 cells with or without TNF-α treatment after the transfection of pGL3B-729 (pGL3B-CADM1) or pGL3-basic (pGL3B-mock). The data represent the means ± S.D. of triplicate determinations and are presented relative to pGL3-basic activity in the absence of TNF-α. **P < 0.01 (Student’s *t* test).

To determine whether the down-regulation of p47 expression is associated with the overexpression of CADM1 in ATLL cells, we transfected with the p47 expression vector into HTLV-1-related cell lines (HUT102 and KK1) and analyzed for the expression of CADM1 and NF-κB signaling proteins, NEMO and IκBα. In all the HTLV-1-related cell lines expressing high levels of CADM1, the enforced expression of p47 significantly down-regulated CADM1 along with the efficient degradation of NEMO and increased total IκBα (Figs 5A and S4A), resulting in the reduction of the cell growth rates in HUT102 and KK1 (Fig. 5B). On the other hand, shRNA-mediated down-regulation of p47 in the Jurkat or MOLT4/T-ALL cell lines induced the accumulation of NEMO with a reduction of the total IκBα and enhanced phosphorylation of IκBα, resulting in the induction of CADM1 expression and acceleration of cell growth via activation of the canonical NF-κB signaling pathway under TNF-α stimulation (Figs 5C,D and S4B). We also confirmed that p47 down-regulation in these cell lines significantly activated the *CADM1* promoter after TNF-α stimulation (Fig. 5E). These results suggest that p47 down-regulation in ATLL cells may contribute to the enhanced expression of CADM1 via the activation of the canonical NF-κB signaling pathway.

### p47 is constitutively degraded by the lysosome-dependent pathway in ATLL

**Figure 6:**
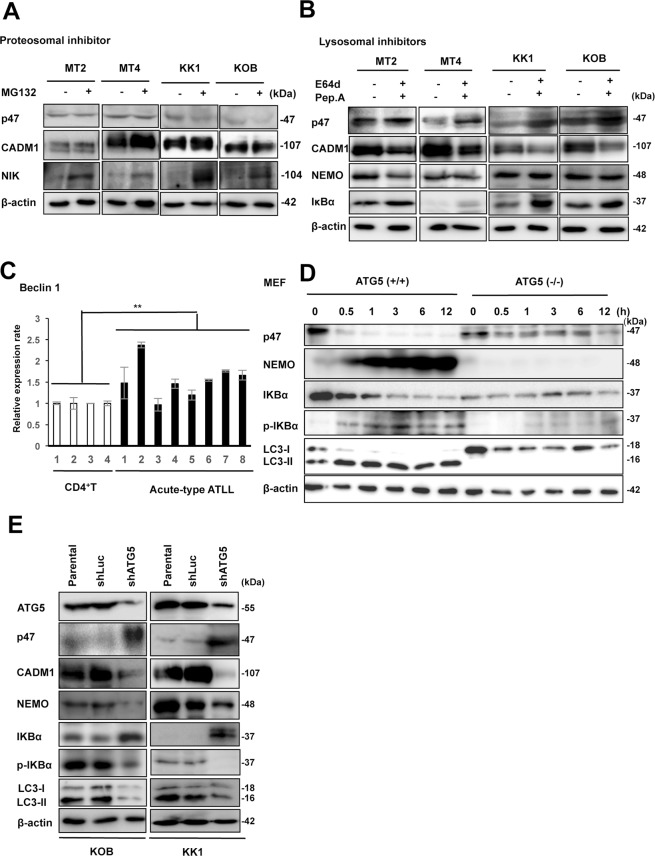
p47 protein is efficiently degraded by the lysosome-dependent pathway in ATLL-related cell lines. (**A**) Immunoblot analysis of p47, CADM1, and NIK was performed in two HTLV-1-infected T-cell lines (MT2 and MT4) and two ATLL cell lines (KK1 and KOB) after treatment with or without MG132 for 48 hours. β-actin was used as a loading control. The cropped gels/blots are used in the figure, and the full-length gels/blots are presented in Supplementary Fig. S7. (**B**) Immunoblot analysis of p47, CADM1, NEMO, and IκBα was performed in two HTLV-1-infected T-cell lines (MT2 and MT4) and two ATLL cell lines (KK1 and KOB) after treatment with or without E64d and pepstatin A for 48 hours. β-actin was used as a loading control. The cropped gels/blots are used in the figure, and the full-length gels/blots are presented in Supplementary Fig. S7. (**C**) Relative expression of Beclin 1 was determined by quantitative RT-PCR in CD4^+^ T-cells from four healthy cases and leukemia cells from eight acute-type ATLL patients. The data represent the means ± S.D. of triplicate experiments and are presented relative to control CD4^+^ T-cells (lane 1). **P < 0.01 (Student’s *t* test). (**D**) After autophagy-deficient primary Atg5(−/−) MEF cells and wild-type primary Atg5(+/+) MEF cells as control were cultured under starvation for each indicated time, and immunoblot analysis of p47 and various NF-κB and autophagy-related signaling molecules was performed using each specific antibody. The cropped gels/blots are used in the figure, and the full-length gels/blots are presented in Supplementary Fig. S7. (**E**) Two ATLL cell lines (KOB and KK1) were transfected using an shRNA expression vector for ATG5 (shATG5) or luciferase as a control (shLuc), and the transfected cells were analyzed for the expression of p47 and protein with various NF-κB and autophagy-related signaling molecules by immunoblotting. β-actin was used as a loading control. The cropped gels/blots are used in the figure, and the full-length gels/blots are presented in Supplementary Fig. S7.

Because there are two major cellular protein degradation systems, the proteasomal or lysosomal degradation pathways, we used inhibitors specific for proteasomal or lysosomal degradation to assess which pathway was involved in the p47 protein degradation in ATLL cells. After treatment of the two HTLV-1-infected cell lines (MT2 and MT4) and two ATLL-derived cell lines (KK1 and KOB) with the proteasomal inhibitor MG132, p47 and CADM1 protein levels were not affected, although NIK levels were increased as a positive control (Fig. 6A). On the other hand, p47 protein levels were restored by inhibiting the lysosome pathways via treatment with the lysosomal inhibitors E64d and pepstatin A in two HTLV-1-infected and two ATLL cell lines (Fig. 6B). Furthermore, this increase in p47 protein expression was accompanied by the down-regulation of CADM1 expression with an increase in IκBα, consistent with the direct inhibition of IκBα protein degradation by the lysosomal inhibitors as previously reported^26^. In addition, the restored p47 expression did not change the NEMO protein levels under treatment with the lysosomal inhibitors (Fig. 6B), a finding that is also in accordance with a previous report that p47 induces NEMO protein degradation through the lysosomal pathway^15^. Therefore, it was suggested that p47 protein is efficiently degraded by a lysosome-dependent mechanism in HTLV-1-infected and ATLL cells.

Because both the activation of the NF-κB pathway and lysosomal/autophagy were reported in HTLV-1-infected cells, we determined the expression of the autophagy-regulatory genes *Beclin 1* (the mammalian orthologue of yeast Atg6) and *LC3B* (microtubule-associated protein 1 A/1B-light chain 3) as targets of NF-κB activation^27^, as well as the levels of autophagy activation in ATLL-related cell lines and leukemia cells from ATLL patients. *Beclin 1* was clearly increased in the leukemia cells from ATLL patients (Fig. 6C), and the levels of LC3-II processing (autophagic flux) were also increased in all the HTLV-1-infected and ATLL-derived cell lines (Fig. 4B) and leukemia cells from ATLL patients (Fig. 4D), although the *LC3B* mRNA levels were maintained compared with those in CD4^+^ T lymphocytes (Fig. S5). To determine the relationship between autophagy activation and p47 degradation with NF-κB activation, we used MEFs from ATG5 (−/−) and ATG5 (+/+) mice^28^ and induced autophagy by starvation. In ATG5 (+/+) MEFs, p47 was down-regulated after 30 min of starvation with enhanced LC3-II processing and NF-κB signaling until 12 hours of starvation. On the other hand, in MEFs with ATG5 (−/−), LC3-II processing was never detected, and the p47 level was maintained with no expression of NEMO and inactivation of NF-κB signaling during the starvation (Fig. 6D). When the expression of ATG5 was knocked down by the introduction of shRNA for ATG5 to HTLV-1-related cell lines (KOB and KK1), LC3-II was disappeared; however, p47 was inversely recovered along with reduction of NEMO, phosphorylated IκBα, and CADM1 (Fig. 6E). Therefore, p47 degradation was closely linked to the activation of autophagy and NF-κB pathways, suggesting that activation of the two pathways might be a very important mechanism for the survival of HTLV-1-infected T-cells and ATLL cells.

## Discussion

In the present study, we studied the activation mechanism of *CADM1* expression in HTLV-1-infected T and ATLL cells and found that the down-regulation of p47 through lysosome/autophagy-dependent protein degradation plays an important role in the constitutive activation of the NF-κB pathway in ATLL cells, leading to the overexpression of CADM1. p47, a cofactor of the AAA ATPase p97/VCP that functions to regulate Golgi membrane assembly^29^, negatively regulates the NF-κB pathway via the induction of NEMO degradation^15^. Although the mRNA levels of p47 were maintained in most ATLL patients and ATLL-related cell lines compared with CD4^+^ T cells from healthy subjects, p47 protein was consistently degraded by the lysosome pathway. Furthermore, the lysosome-dependent degradation of p47 was mediated by the activation of the autophagy pathway in ATLL cells. Therefore, the autophagy and NF-κB signaling pathways are reciprocally activated through the degradation of p47 in ATLL cells, inducing the overexpression of CADM1. Thus, the activation of the two pathways might be one of the important events for the survival and maintenance of HTLV-1-infected cells during ATLL development.

The autophagy pathway is an intracellular protein degradation system that utilizes the lysosome for degradation. In normal cells, autophagy functions to degrade nonspecifically cellular proteins and organelles to reuse constituents, such as amino acids, or works as one of the host defenses against infections to degrade invading pathogens, including viruses^30^. Recently, several studies have shown that autophagy is constitutively activated in certain cancer cells to maintain their efficient cell growth^31^. In HTLV-1-infected T-cells, Tax has been shown to activate the early phase of NF-κB activation through interaction with autophagy-regulatory proteins such as Beclin 1, which promotes the recruitment of IKK complex to an autophagy molecular complex containing Beclin 1 and induces efficient autophagosome formation^32,33^. In addition, the NF-κB pathway is known to activate the transcription of the genes encoding autophagy-regulatory proteins such as LC3B, ATG5, and Beclin 1 to promote the initiation of autophagy^27,34^. Notably, our expression studies using cDNA microarray and quantitative RT-PCR showed an increased tendency for the expression of these autophagy-related genes (data not shown and Fig. 6C). Therefore, it is speculated that the activation of the NF-κB pathway may up-regulate the autophagy-lysosome pathway in HTLV-1-infected and ATLL cells and that the enhanced degradation of p47 by targeting it to the autophagy pathway via an unknown mechanism may promote further activation of the NF-κB pathway, likely contributing to the sustained activation of autophagy. Interestingly, the yeast homolog of p47 was reported to play an essential role in autophagosome biogenesis along with p97/VCP^35^. Further studies are required to clarify the roles of p47 in the regulation of autophagy in relation to NF-κB signaling and the interaction with p97/VCP, which may also provide an important clue regarding how HTLV-1 escapes autophagy upon its invasion into the host cell. It may also be valuable to clarify the deregulation of expression of p47 in other cancers because the NF-κB pathway is known to be highly activated in multiple cancer types, and autophagy is activated under cellular stress conditions with viral infection^36^, providing a possible link between the NF-κB signaling and cell stress responses in cancer cells.

CADM1 is a cell adhesion molecule that participates in the intercellular adhesion of epithelial cells via homophilic and heterophilic interactions. During T-cell differentiation, CADM1 is not expressed. However, upon the infection of HTLV-1, CADM1 is transcriptionally activated via the binding of the NF-κB transcription factors to the *CADM1* promoter (this study). Because CADM1 plays important roles in cell adhesion, migration, and invasion of ATLL cells^2–4^, CADM1 expression may help HTLV-1-infected T-cells or ATLL cells to move to a more favorable environment for efficient cell growth during leukemogenesis. It was also noticeable that CADM1 expression appears to be gradually increased with the disease progression of ATLL (Fig. 1A). This may reflect the degree of the malignant phenotype of ATLL cells and may be caused by the increased activation of the NF-κB pathway during leukemogenesis. Indeed, recent genomic analyses of ATLL patients revealed frequent mutations of the genes in the canonical TCR-NF-κB signaling pathway, which was found to be the most affected pathway by somatic mutations in ATLL^11^. Moreover, activation of PI3K/AKT signaling contributes to constitutive activation of the NF-κB pathway through PTEN inactivation via NDRG2/PP2A dysregulation^37,38^ or CCR4 mutation^11,39^ (Fig. S6). Thus, the accumulation of genetic alterations may elevate the activation of NF-κB signaling along with p47 degradation, which may increase the level of CADM1 expression in ATLL cells. Because the NF-κB pathway is crucial for the survival of various cancer cells, several NF-κB inhibitors have now been developed under preclinical or clinical trials^40^. Because inhibition of the autophagy pathway efficiently decreases CADM1 expression via NF-κB signaling inactivation, a new concept that NF-κB inhibitors induce autophagy suppression may be adapted for the suppression of not only ATLL cell survival but also organ invasion with resistance to anti-cancer drugs as a valuable target for ATLL.

## Supplementary information

Supplementary information
