# Supplementary material for: Degradation of p47 by autophagy contributes to CADM1 overexpression in ATLL cells through the activation of NF-κB
Source: Sci Rep. 2019 Mar 5;9:3491. doi: 10.1038/s41598-019-39424-7 (PMC6400899; doi:10.1038/s41598-019-39424-7)
Supplement: Supplementary file 1 — Supplementary information [file 41598_2019_39424_MOESM1_ESM.pdf]

Supplementary Information for

Degradation of p47 by autophagy contributes to CADM1 overexpression in ATLL cells through the activation of NF-κB

Bidhan Sarkar[1,6)], Ichiro Nishikata[1,6)], Shingo Nakahata[1)], Tomonaga Ichikawa[1)], Toshiyuki Shiraga[1,2)], Hasi Rani Saha[1)], Masahiro Fujii[3)], Yuetsu Tanaka[4)], Kazuya Shimoda[5)] and Kazuhiro Morishita[1)]

[1]Division of Tumor and Cellular Biochemistry, Department of Medical Sciences, University of Miyazaki, Miyazaki, Japan

[2]Department of Foods and Human Nutrition, Faculty of Human Life Sciences, Notre Dame Seishin University, Okayama, Japan;

[3]Division of Virology, Niigata University Graduate School of Medical and Dental Sciences, Niigata, Japan.

[4]Department of Immunology, Graduate School of Medicine, University of the Ryukyus, Okinawa, Japan.

[5]Division of Gastroenterology and Hematology, Department of Internal Medicine, Faculty of Medicine, University of Miyazaki, Miyazaki, Japan

To whom correspondence should be addressed. E-mail: kmorishi@med.miyazaki-u.ac.jp

Supplementary Tables S1 and S2
Supplementary Figures S1 to S7
Supplementary references

Supplementary table S1 List of primers for semiquantitative RT-PCR

Name	Sequence	
**Human**	**Forward (5' to 3')**	**Reverse (5' to 3')**
β-actin (255 bp)	AAGAGATGGCCACGGCTGCT	TCCTTCTGCATCCTGTCGGC
β-actin (142 bp)	GACAGGATGCAGAAGGAGAT	TGATCCACATCTGCTGGAAGGT
p47	GAGGGGATGAAGACATTGTGA	GAATGCTGCCTCTTTTCTCCT
CADM1	TCAACACGCCGTACTGTCTG	GTGGGAGGAGGGATAGTTGTG
NEMO	CTTTTGGGGTAGATGCG	GGTTAAATACACATCGGTCTG
Tax (373 bp)	CTCTGGGGGACTATGTTCGGCC	GTACATGCAGACAACGGAGCCT
Tax (250 bp)	CCGGCGCTGCTCTCATCCCGGT	GGCCGAACATAGTCCCCCAGA
	GAGGATGAGGAGAGCTATGAC	
IKB-α	ACAG	AGGAGGGTAACACAAACCTTGACAG
A20	GAGAGCACAATGGCTGAACA	TCCAGTGTGTATCGGTGCAT
CYLD	TGCCTTCCAACTCTCGTCTTG	AATCCGCTCTTCCCAGTAGG
ATG5	GCAAGCCAGACAGGAAAAAG	GACCTTCAGTGGTCCGGTAA

Supplementary table S2 List of primers for quantitative RT-PCR

Name	Sequence	
**Human**	**Forward (5' to 3')**	**Reverse (5' to 3')**
β-actin	GACAGGATGCAGAAGGAGAT	TGATCCACATCTGCTGGAAGGT
p47	GAGGGGATGAAGACATTGTGA	GAATGCTGCCTCTTTTCTCCT
CADM1	TCAACACGCCGTACTGTCTG	GTGGGAGGAGGGATAGTTGTG
NEMO	CTTTTGGGGTAGATGCG	GGTTAAATACACATCGGTCTG
Tax	CTCTGGGGGACTATGTTCGGCC	GTACATGCAGACAACGGAGCCT
IL6	CCACACAGACAGCCACTCACC	CTACATTTGCCGAAGAGCCCTC
INOS	CAGTACGTTTGGCAATGGAGACTGC	GGTCACATTGGAGGTGTAGAGCTTG
BECLIN1	GGCTGAGAGACTGGATCAGG	CTGCGTCTGGGCATAACG
MAP1LC3B	GAGAAGCAGCTTCCTGTTCTGG	GTGTCCGTTCACCAACAGGAAG

Figure S1

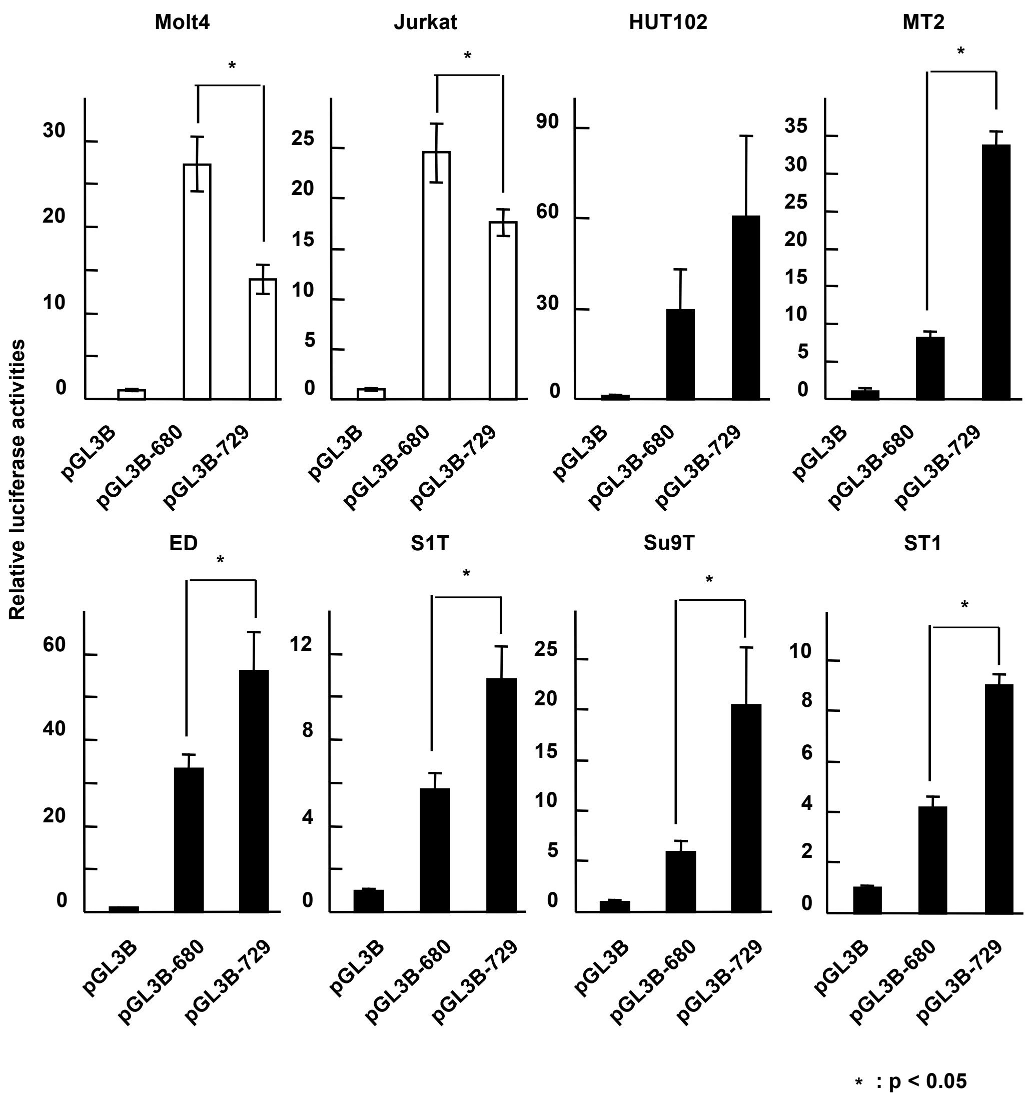

Fig. S1. Identification of transcriptional activator region in the *CADM1* promoter in ATLL.

Luciferase activity of the pGL3B-729 and pGL3B-680 constructs in two T-ALL (Jurkat and MOLT4; white box) and six ATLL-related cell lines (HUT102, MT2, ED, S1T, Su9T, and ST1; black box). Data represent the mean ± S.D. of triplicate determinations and are presented relative to pGL3-basic activity. *$P < 0.05$ (Student's *t* test).

Figure S2

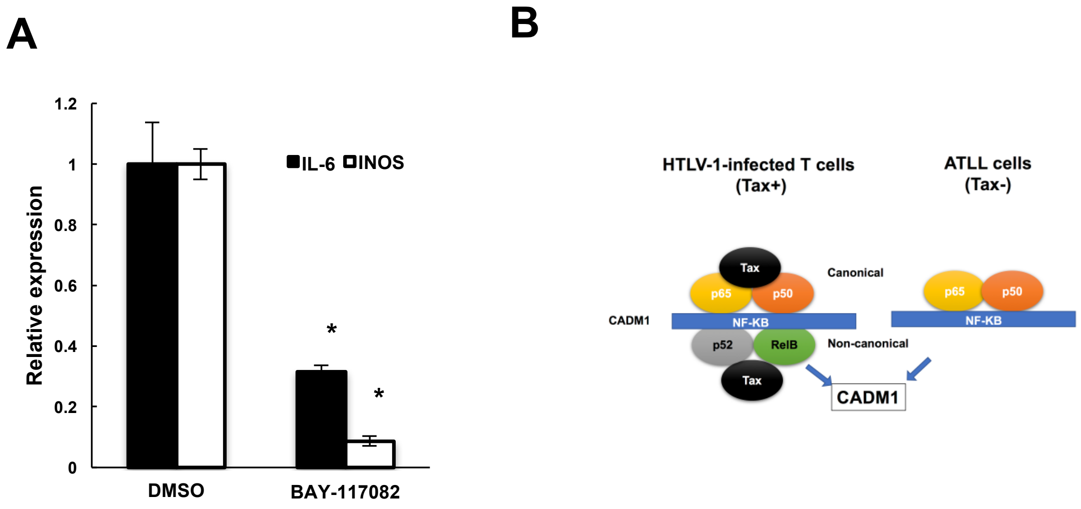

Fig. S2. The activation of NF-κB pathway induces CADM1 expression in ATLL cells.

(A) Real-time RT-PCR analysis for IL-6 and INOS in KK1 12 h after the treatment with 10 μM Bay11-7082. Data represent the mean ± S.D. of triplicate determinations and are presented relative to the untreated control (set as 1). *$P < 0.05$ (Student's *t* test).

(B) Schematic diagram showing the complexes formed on the NF-κB-like binding sequence in the *CADM1* promoter by the NF-κB transcription factors and Tax based on Fig. 2G.

Figure S3

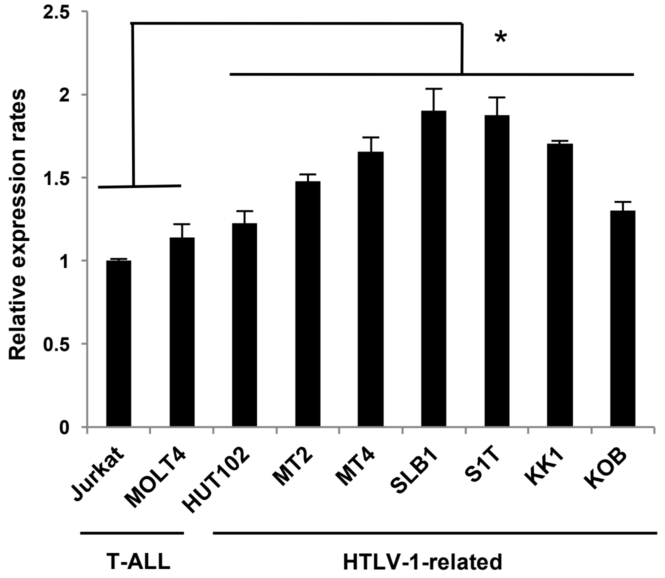

Fig. S3 Expression of NEMO in various leukemia cell lines

Relative expression of NEMO in various leukemia cell lines (two T-ALL and seven HTLV-1-related) was calculated from the immunoblot analysis of NEMO in Fig. 4B. Data represent the mean ± S.D. of triplicate determinations and are presented relative to the expression in Jurkat cells (set as 1). *$P < 0.05$ (Student's *t* test).

Figure S4

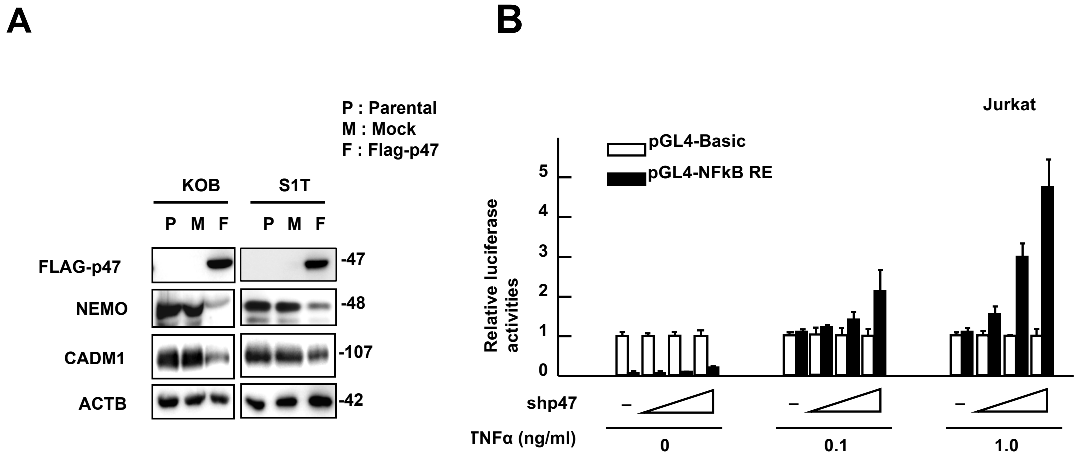

Fig. S4 The expression level of p47 is negatively related to activation of the NF-κB canonical pathway and is important for activation of CADM1 transcription

(A) Immunoblot analysis of FLAG-p47, NEMO, and CADM1 in KOB or S1T cells was performed after transfection of a p47 expression vector or mock vector. β-actin was used as a loading control.

(B) Luciferase activity of the NF-κB-responsive luciferase reporter vector (pGL4-NF-κB-RE) or mock vector (pGL4-Basic) is determined in Jurkat cells after transfection with various concentrations of shRNA-expression vector for p47 (shp47) with or without TNF-α treatment. Data represent the mean ± S.D. of triplicate determinations and are presented relative to the Jurkat cells transfected with pGL4-Basic without TNF-α treatment.

Figure S5

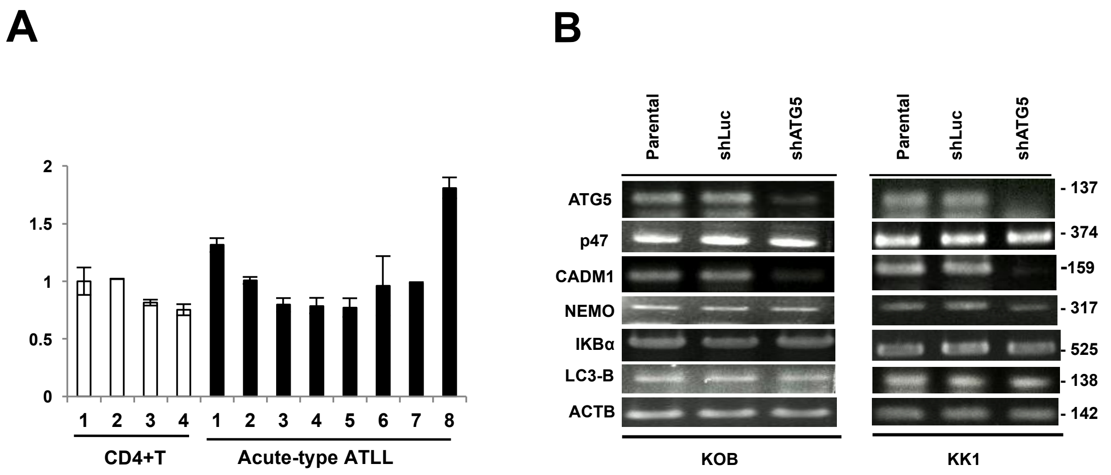

Fig. S5 Expression analysis for *MAP1LC3B/LC3B* in patient samples and for ATG5 with other genes in ATG5 knockdown ATLL cell lines

(A) Real-time RT-PCR analysis for *MAP1LC3B/LC3B* in CD4+ T-cells from four healthy volunteers and ATLL cells from eight acute-type ATLL patients. Data represent the mean ± S.D. of triplicate determinations and are presented relative to the *MAP1LC3B/LC3B* expression of control $CD4^{+}$ T-cells (lane 1).

(B) Semiquantitative RT-PCR analysis for ATG5 in two ATLL cell lines (KOB and KK1) with transfection of shRNA specific for ATG5 or for luciferase as a control. Expression of p47, CADM1, NEMO, IκBα and LC3-B was determined in each indicated cell line by each pair of primers.

Figure S6

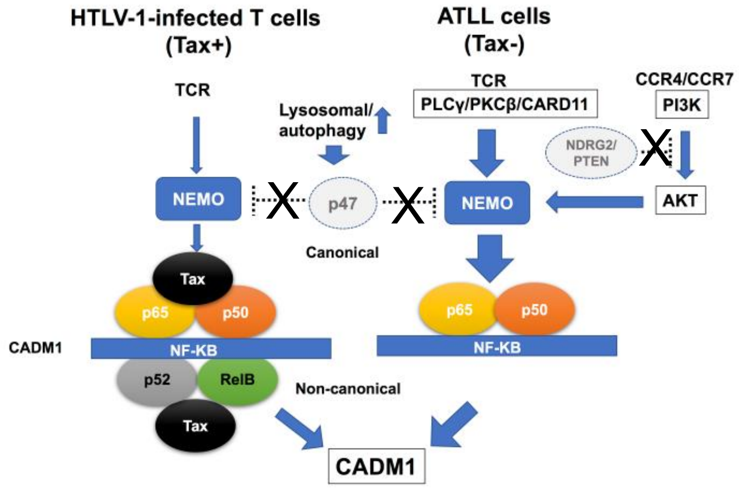

Fig. S6 Schematic diagram showing activation mechanism of CADM1 in HTLV-1-infected T-cells and ATLL cells

CADM1 is transcriptionally activated in HTLV-1-infected T- and ATLL cells. In HTLV-1-infected T-cells with high HTLV-1/Tax expression, Tax activates NF-κB signaling pathways by its direct interaction to p65/p50 complex in canonical pathway and to p52/RelB in non-canonical pathway and p47 degraded by lysosomal/autophagy pathway stabilizes NEMO to activate canonical pathway. On the other hand, in ATLL cells, several mutations (PLCγ, PKCβ, and CARD11) under TCR signaling enhance activation of NF-κB canonical pathway, which is also activated by PI3K/AKT signaling through CCR4 or CCR7 mutations and inactivation of PTEN by NDRG2 inactivation.[1,2]

Fig. S7 Original gels and blots

Fig. 1C

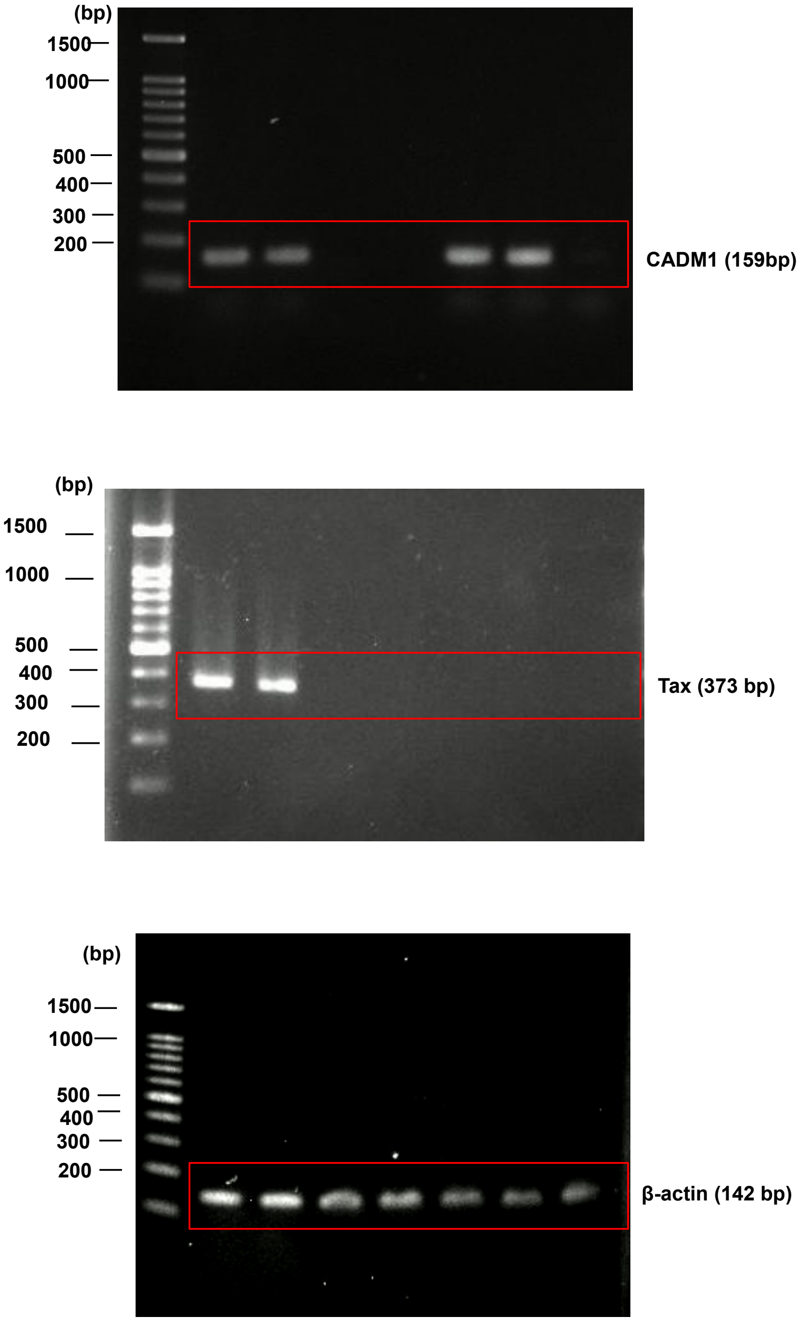

Fig. 2G

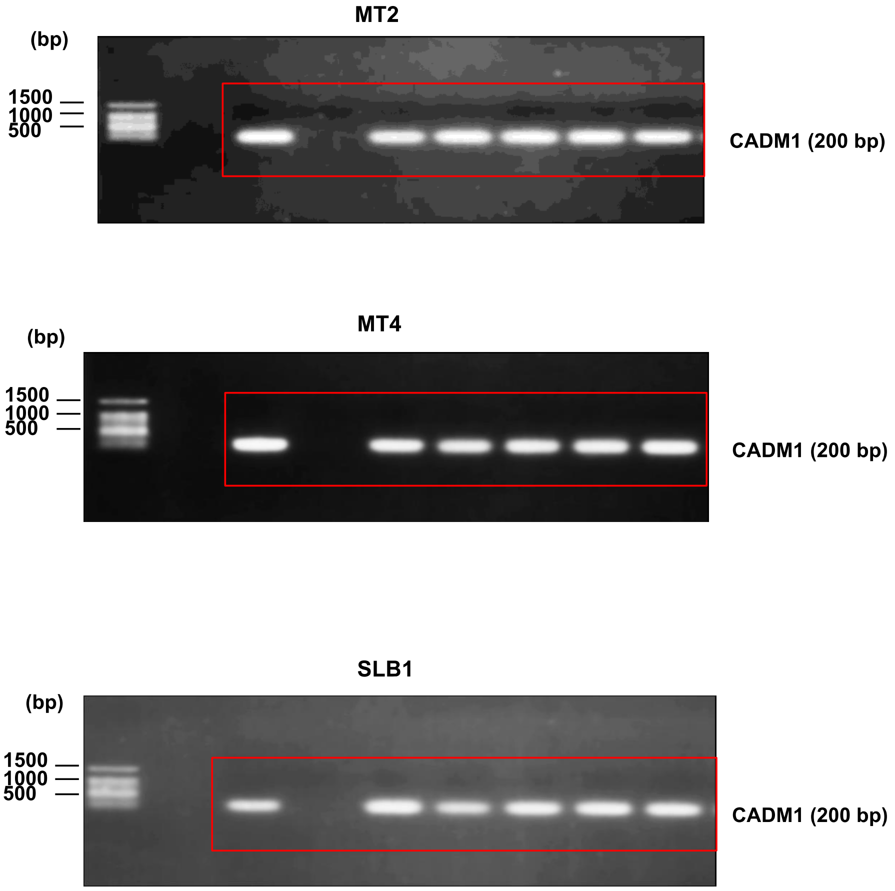
MT2
(bp)
1500
1000
500
CADM1 (200 bp)
MT4
(bp)
1500
1000
500
CADM1 (200 bp)
SLB1
(bp)
1500
1000
500
CADM1 (200 bp)

Fig. 2G

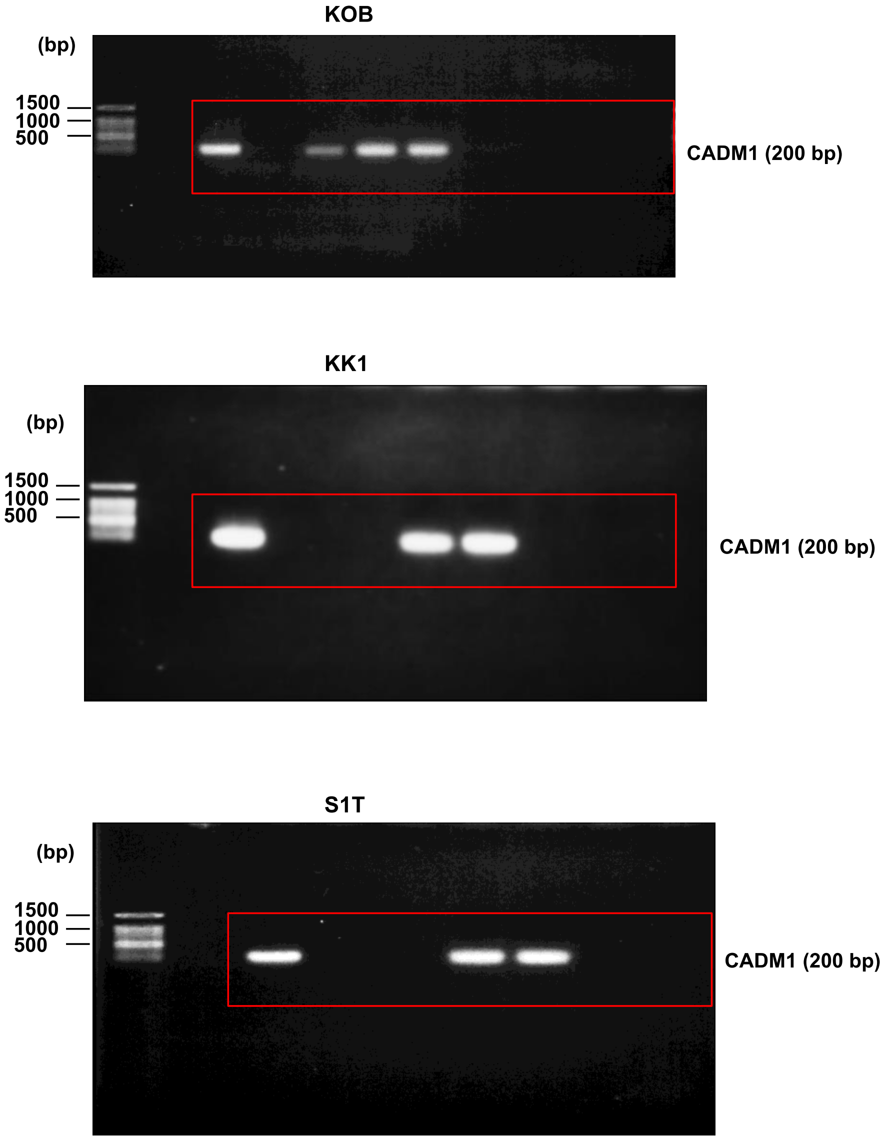
KOB
(bp)
1500
1000
500
CADM1 (200 bp)
KK1
(bp)
1500
1000
500
CADM1 (200 bp)
S1T
(bp)
1500
1000
500
CADM1 (200 bp)

Fig. 3A

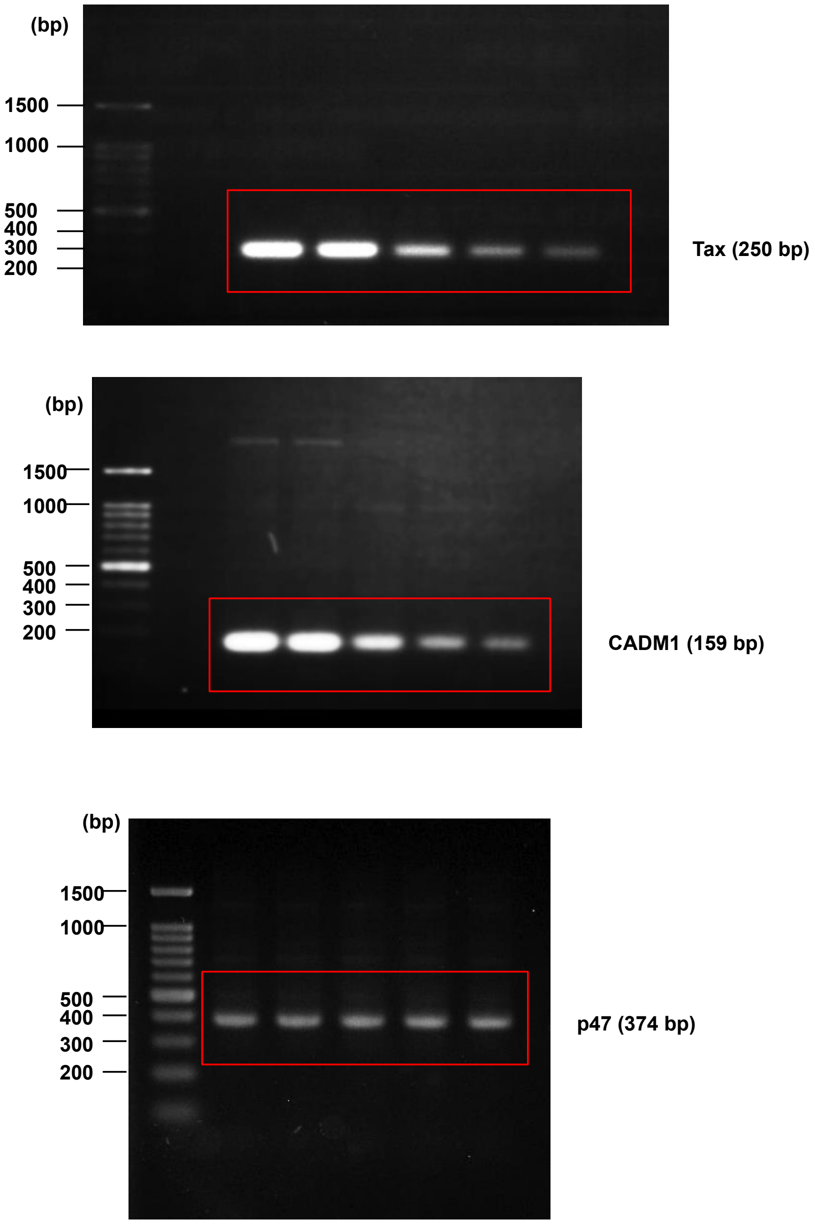
(bp)
1500
1000
500
400
300
200
Tax (250 bp)
(bp)
1500
1000
500
400
300
200
CADM1 (159 bp)
(bp)
1500
1000
500
400
300
200
p47 (374 bp)

Fig. 3A

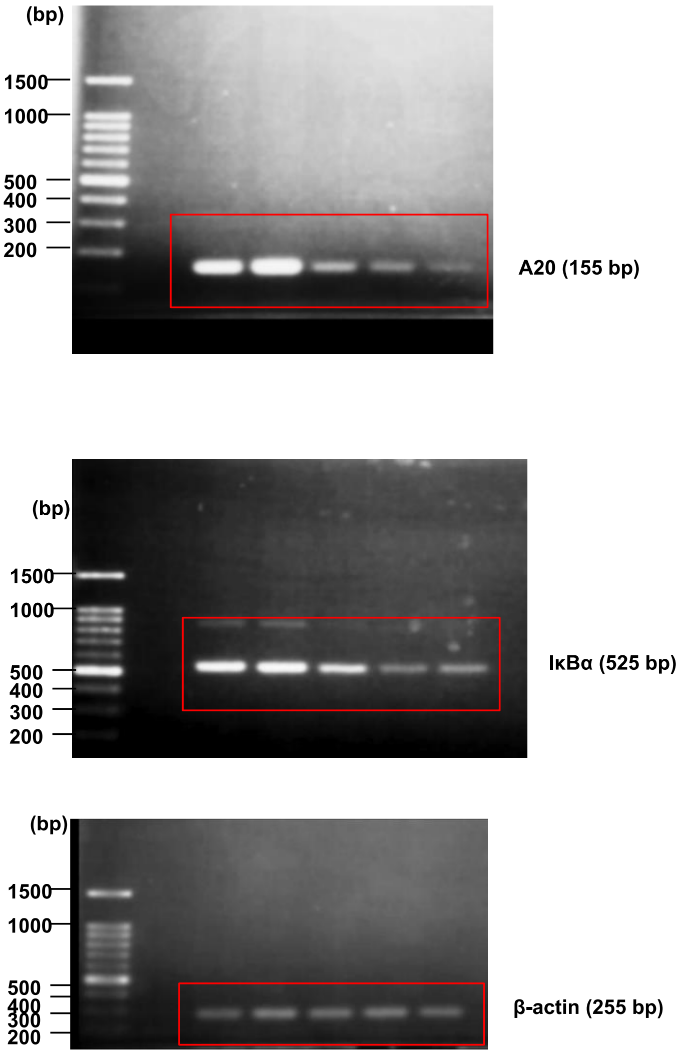
(bp)
1500
1000
500
400
300
200
A20 (155 bp)
(bp)
1500
1000
500
400
300
200
IκBα (525 bp)
(bp)
1500
1000
500
400
300
200
β-actin (255 bp)

Fig. 4A

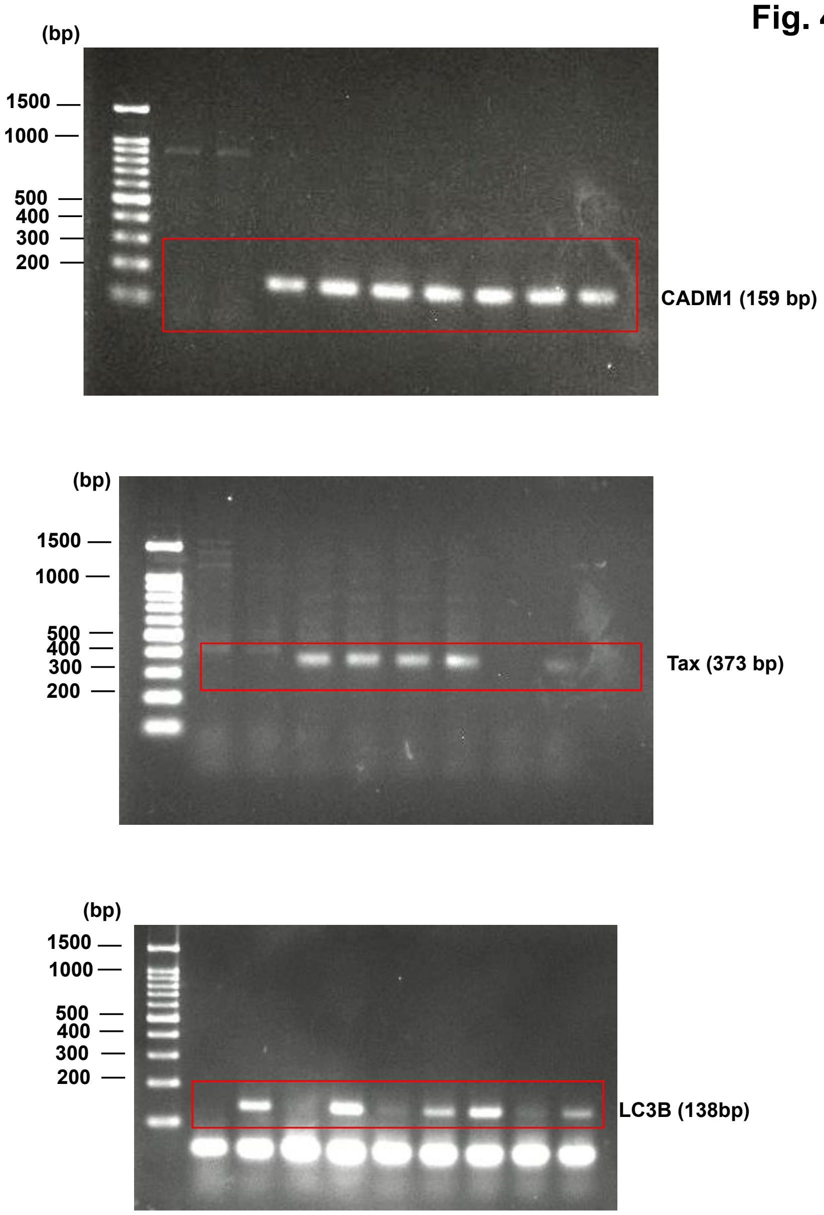
(bp)
1500
1000
500
400
300
200
CADM1 (159 bp)
(bp)
1500
1000
500
400
300
200
Tax (373 bp)
(bp)
1500
1000
500
400
300
200
LC3B (138bp)

Fig. 4A

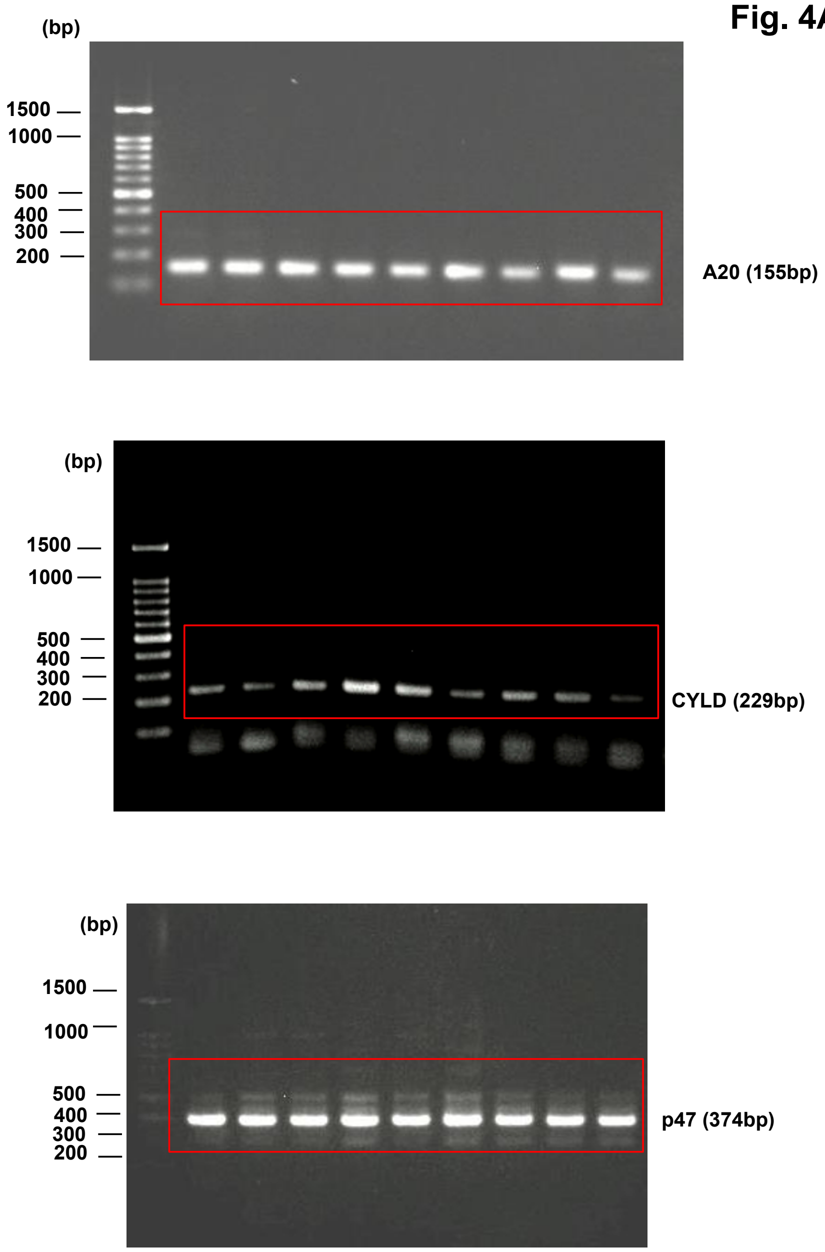

(bp)
1500
1000
500
400
300
200
A20 (155bp)
(bp)
1500
1000
500
400
300
200
CYLD (229bp)
(bp)
1500
1000
500
400
300
200
p47 (374bp)

Fig. 4A

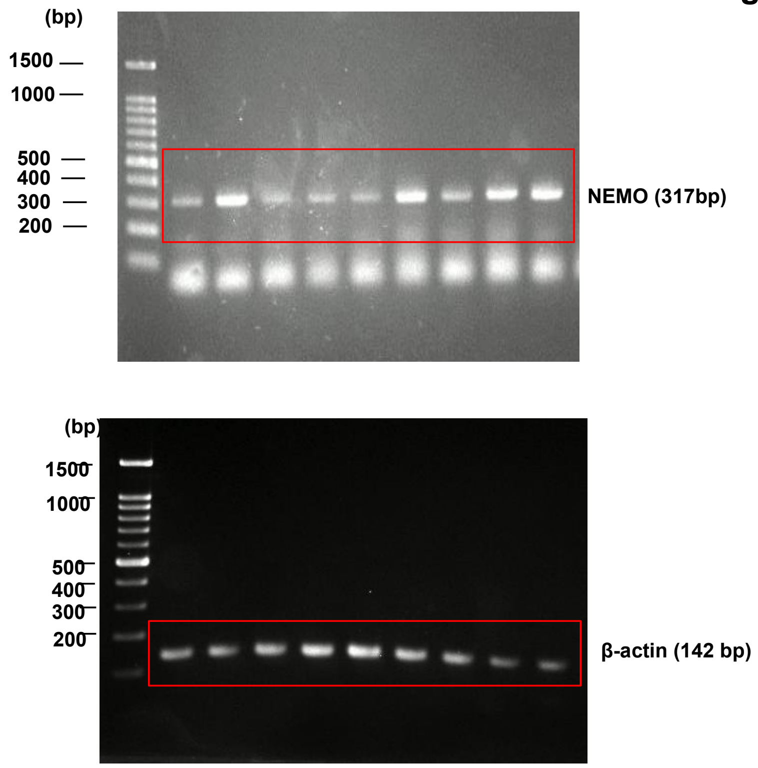
(bp)
1500
1000
500
400
300
200
NEMO (317bp)
(bp)
1500
1000
500
400
300
200
β-actin (142 bp)

Fig.4B

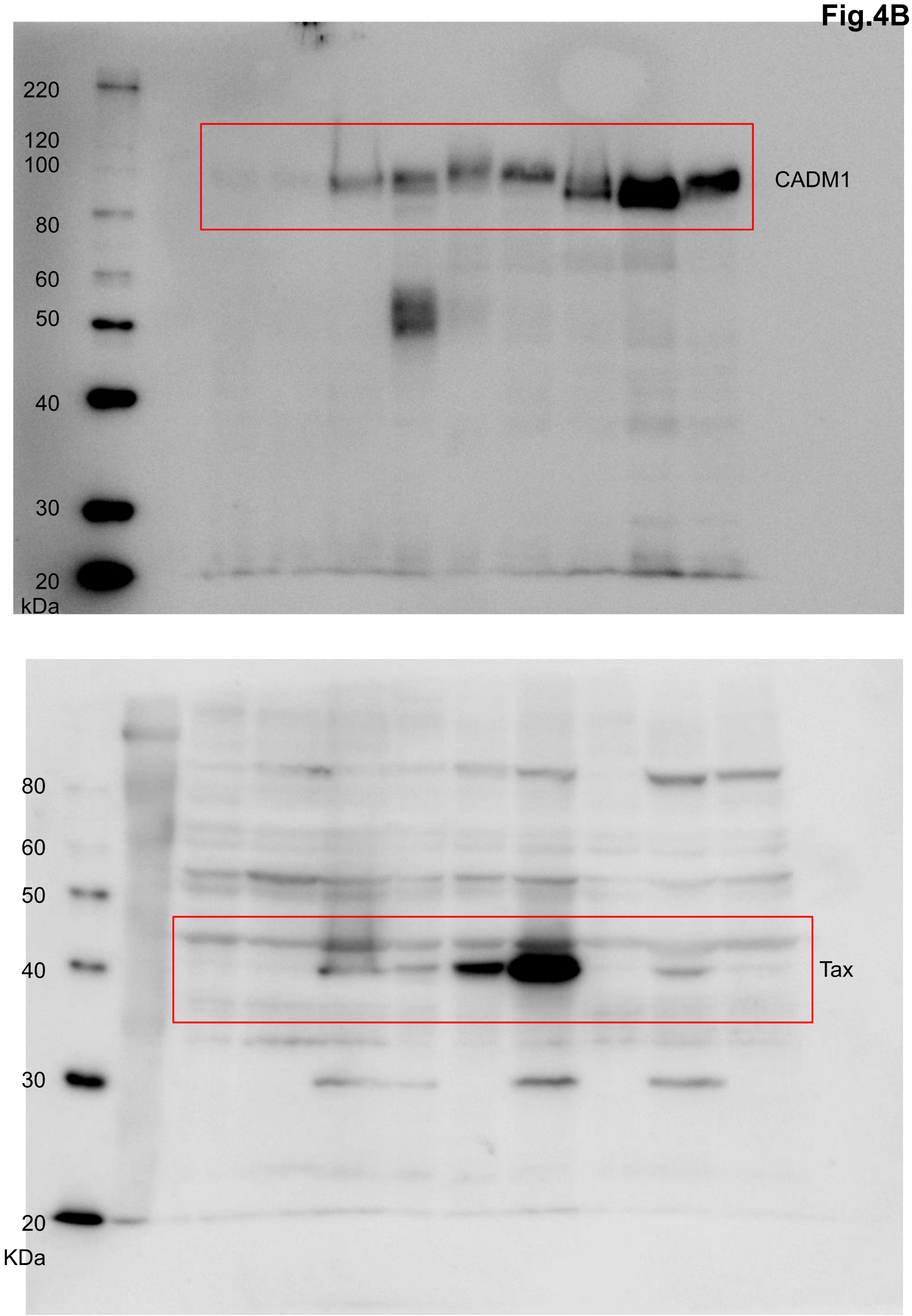

Fig.4B

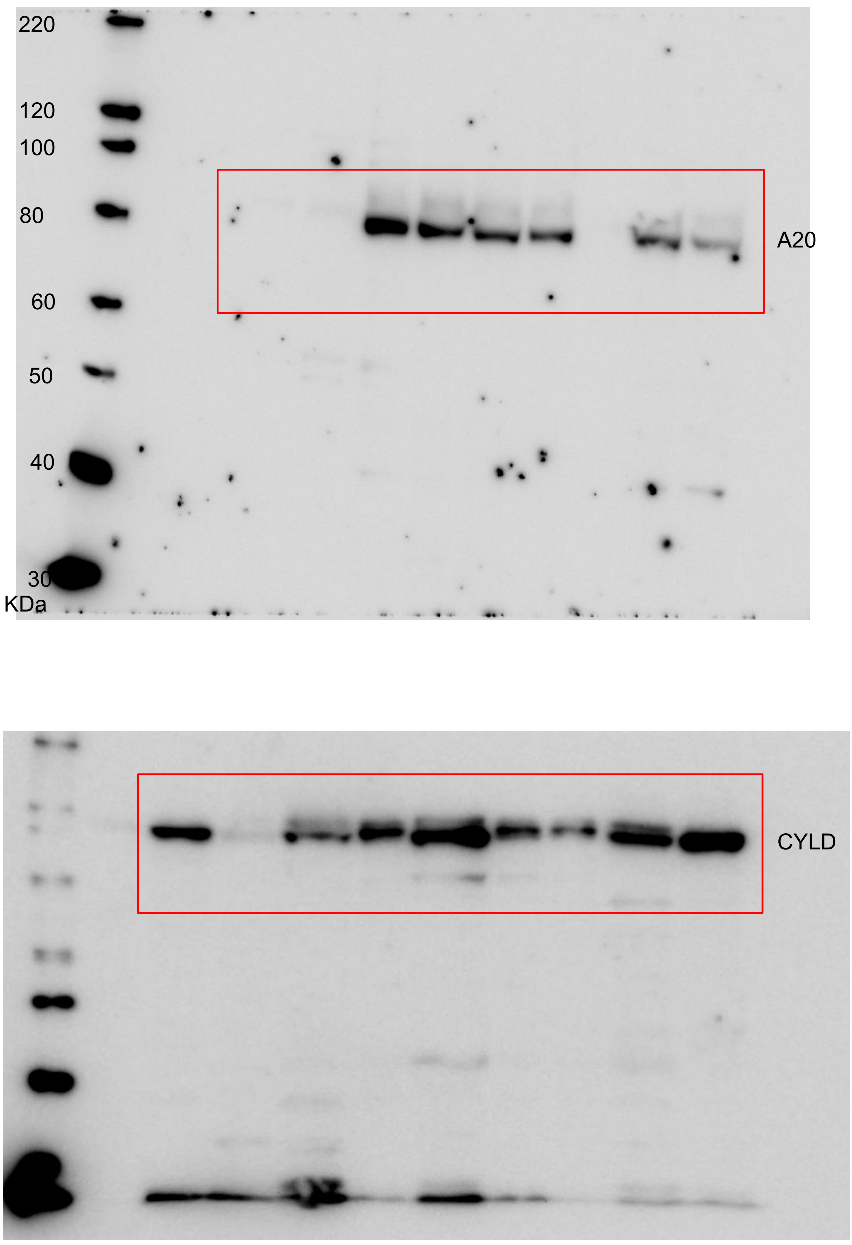

Fig.4B

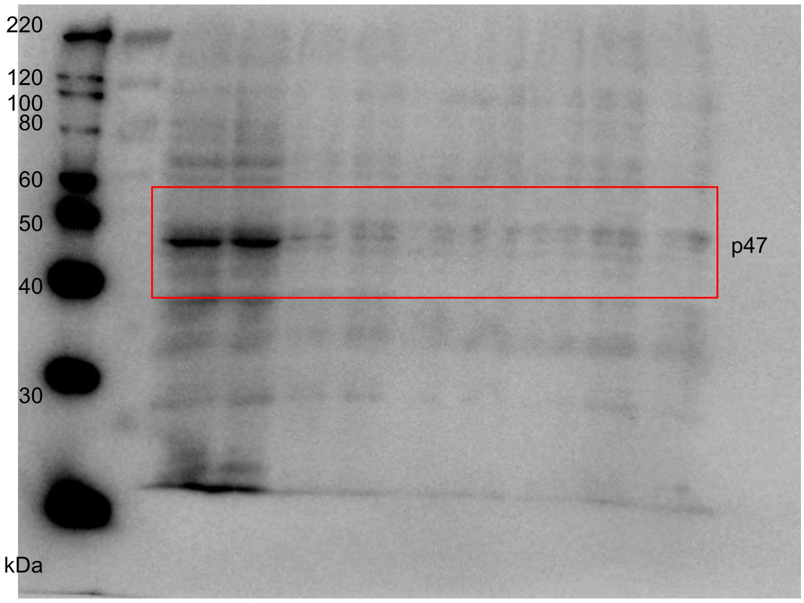

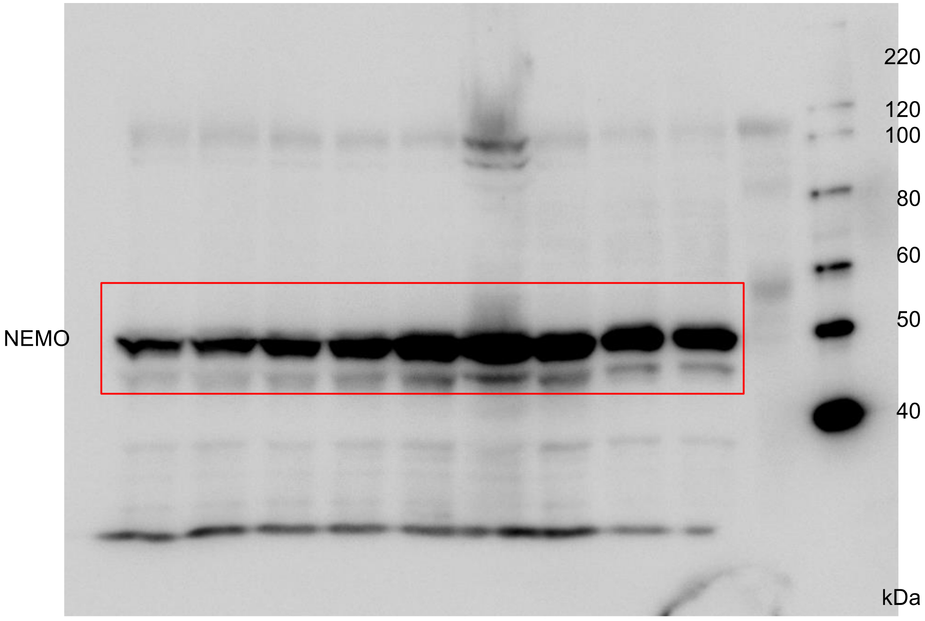

Fig.4B

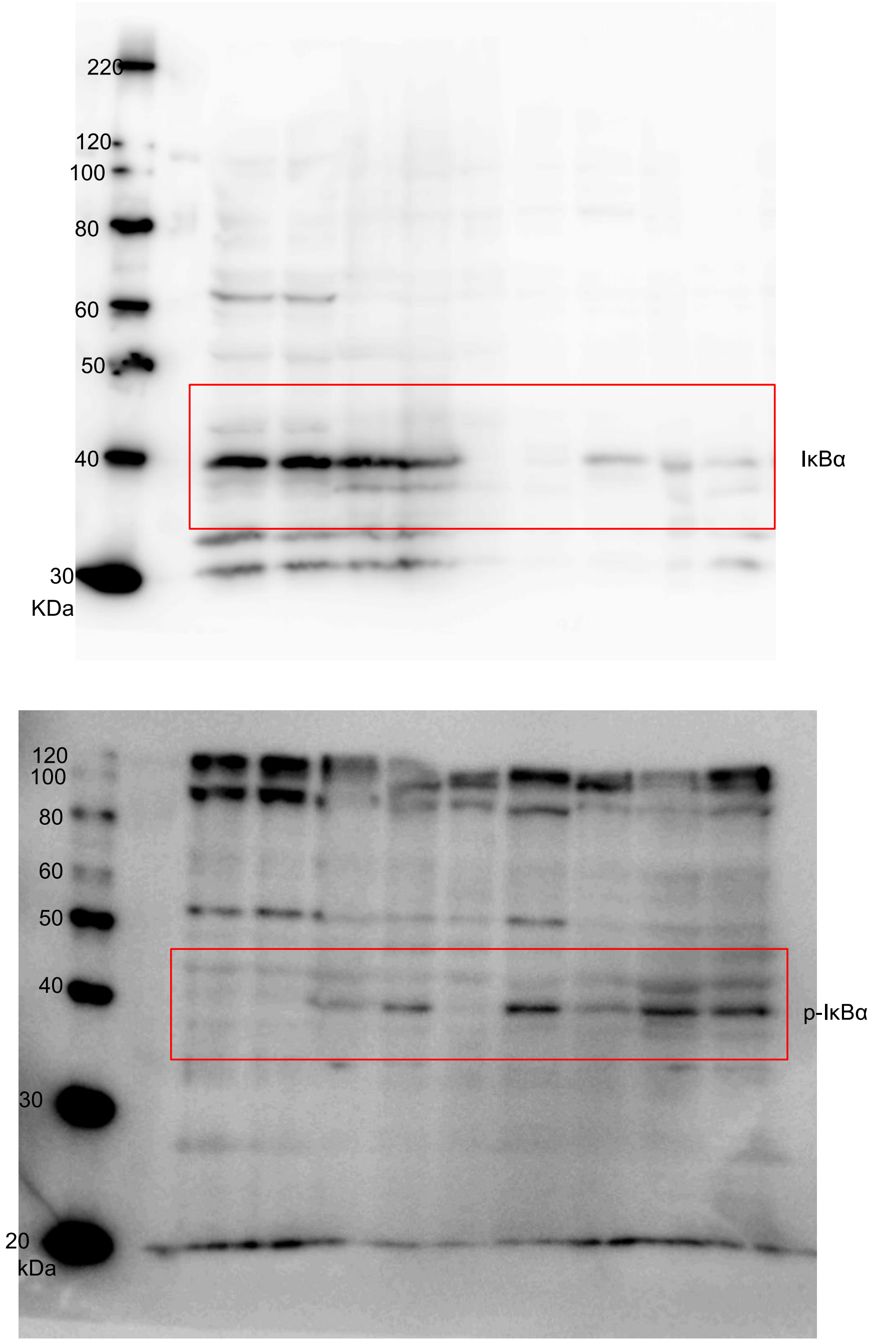
220
120
100
80
60
50
40
30
KDa
IκBα
120
100
80
60
50
40
30
20
kDa
p-IκBα

Fig.4B

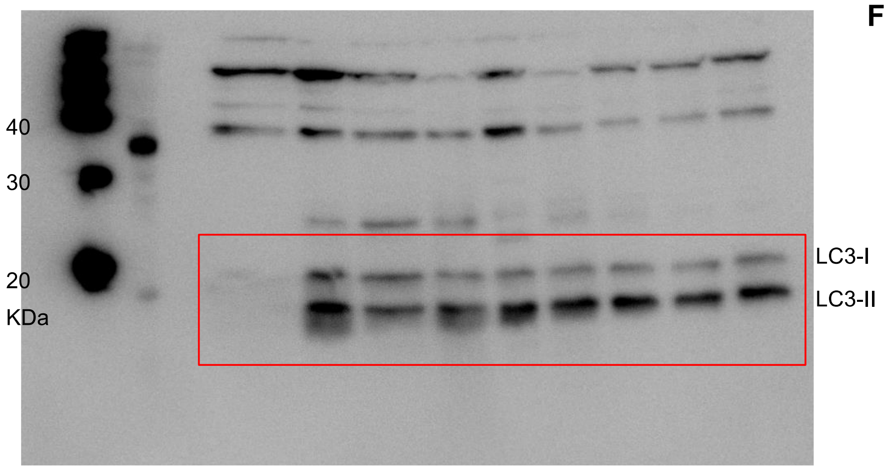

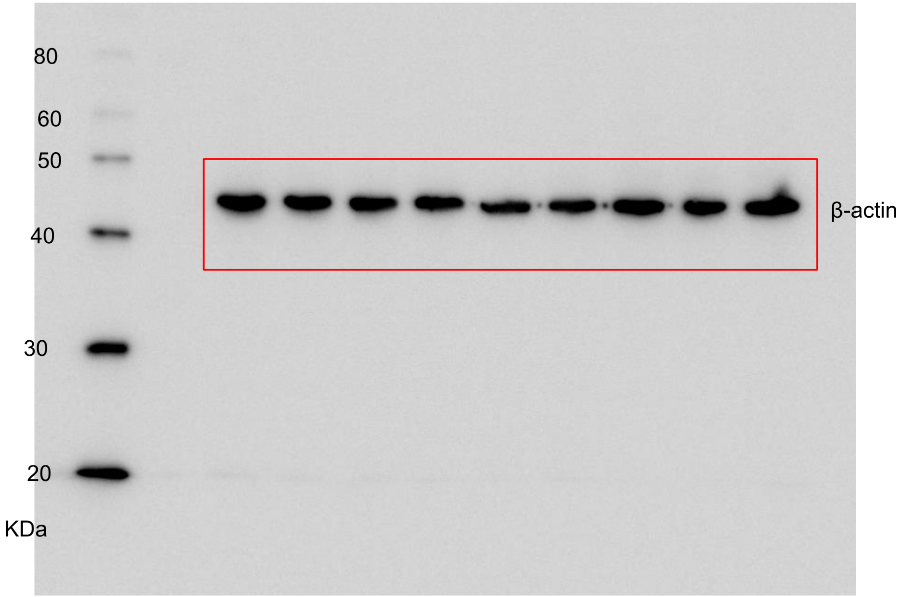

Fig.4D

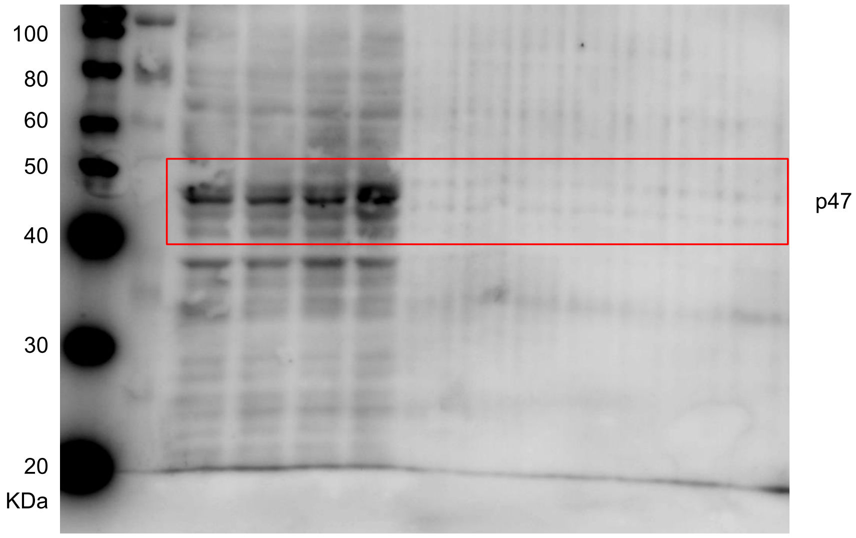

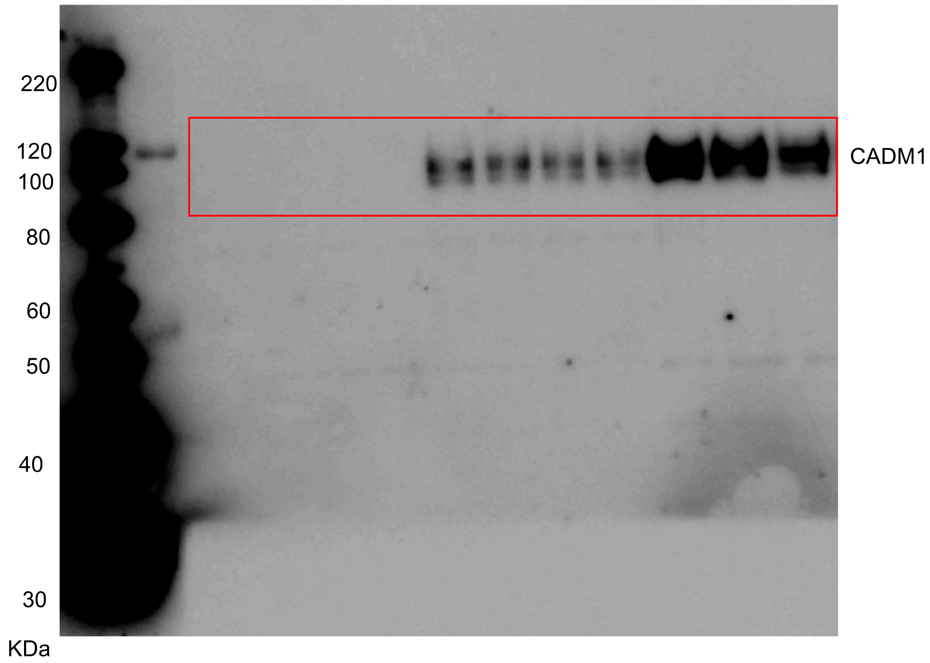

Fig.4D

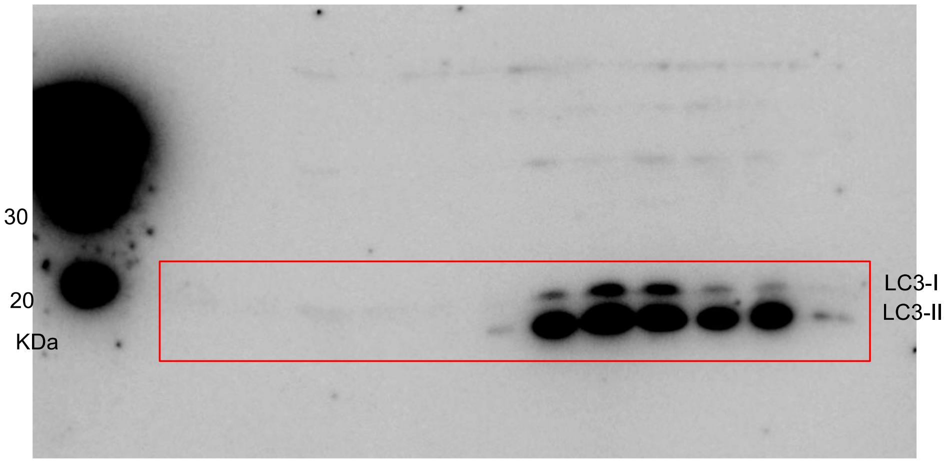

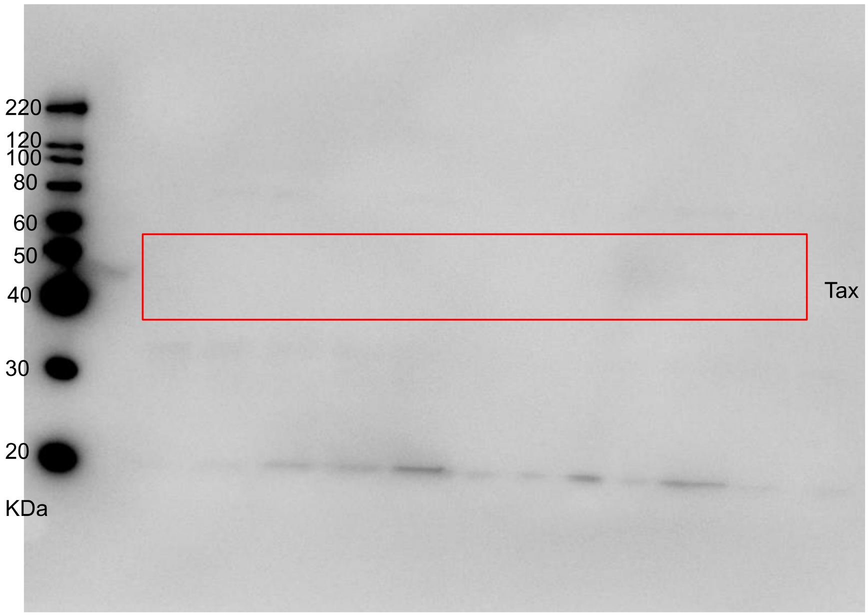

Fig.4D

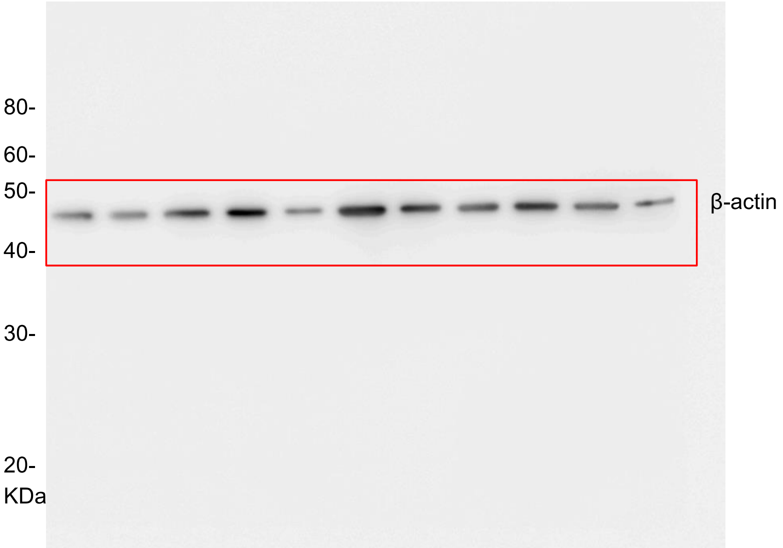

Fig.5A/KK1

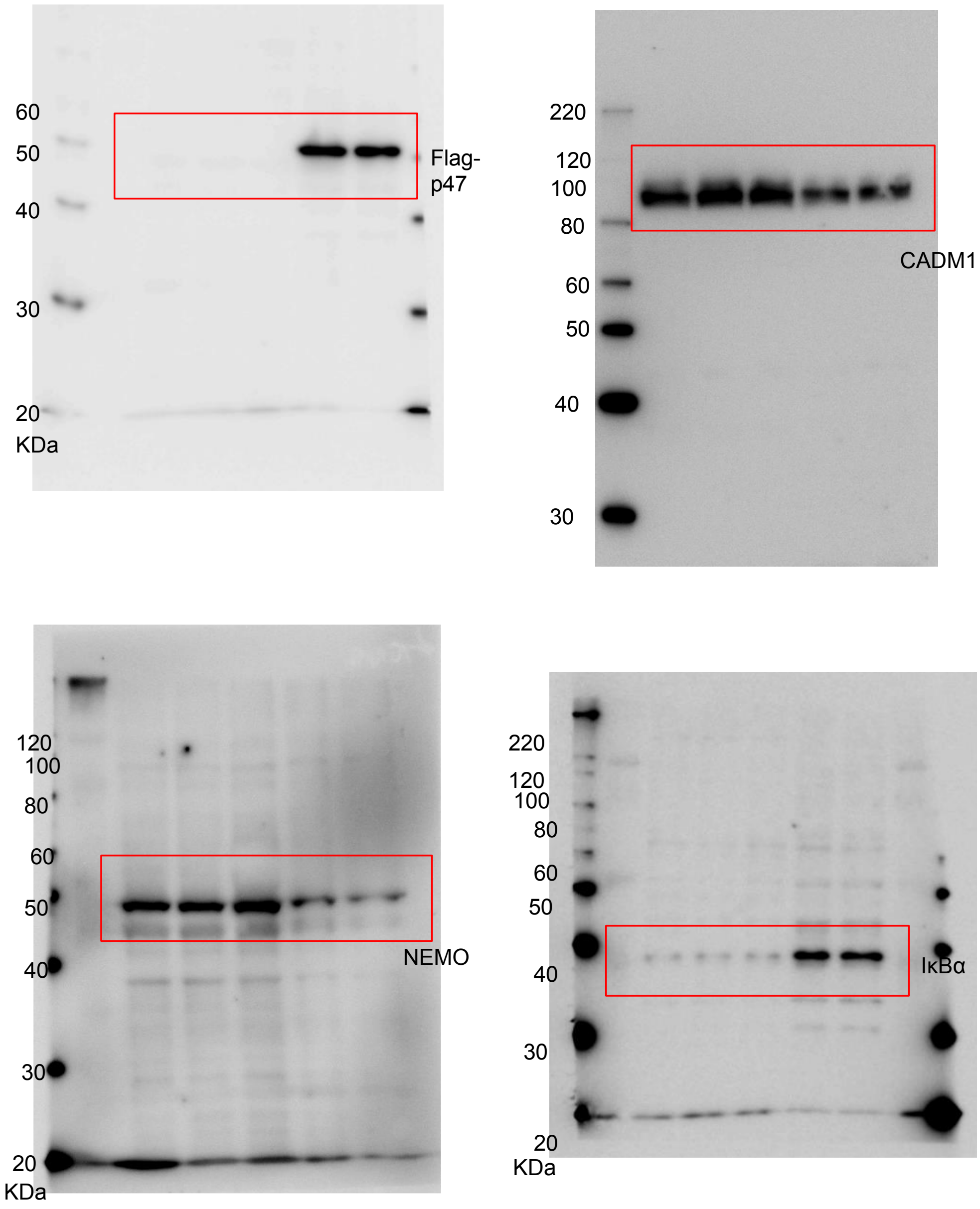
60
50
40
30
20
KDa
Flag-
p47
220
120
100
80
60
50
40
30
CADM1
120
100
80
60
50
40
30
20
KDa
NEMO
220
120
100
80
60
50
40
30
20
KDa
IκBα

Fig.5A
/KK1

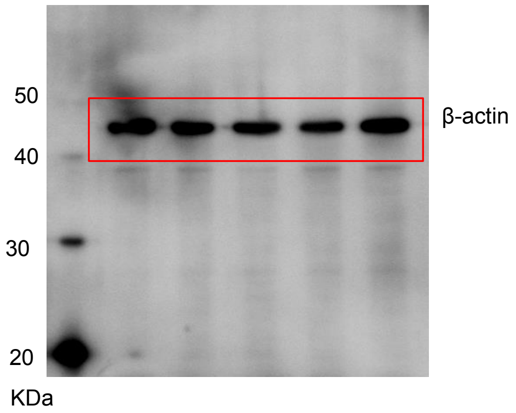

Fig.5A/
HUT102

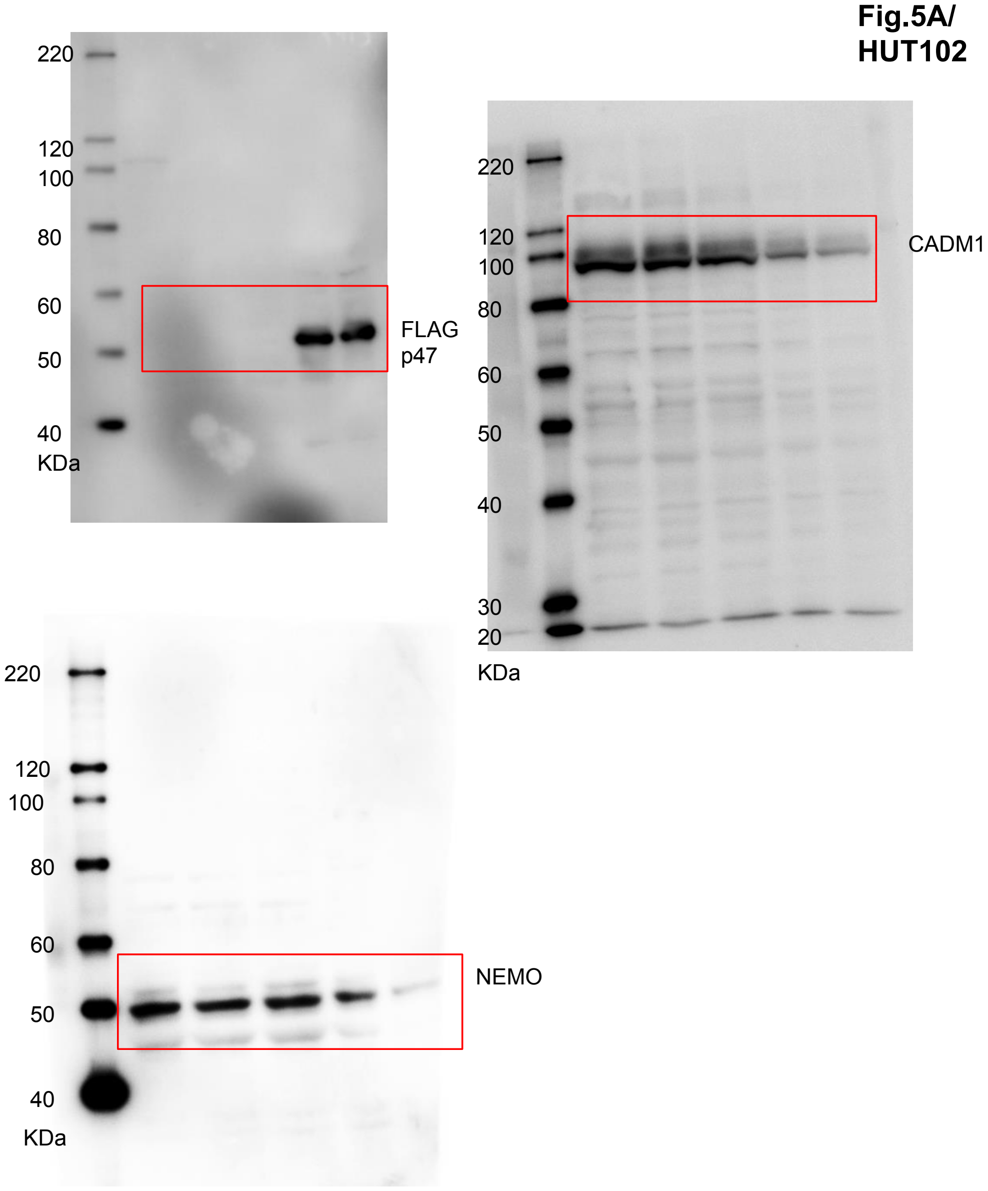

Fig.5A /HUT102

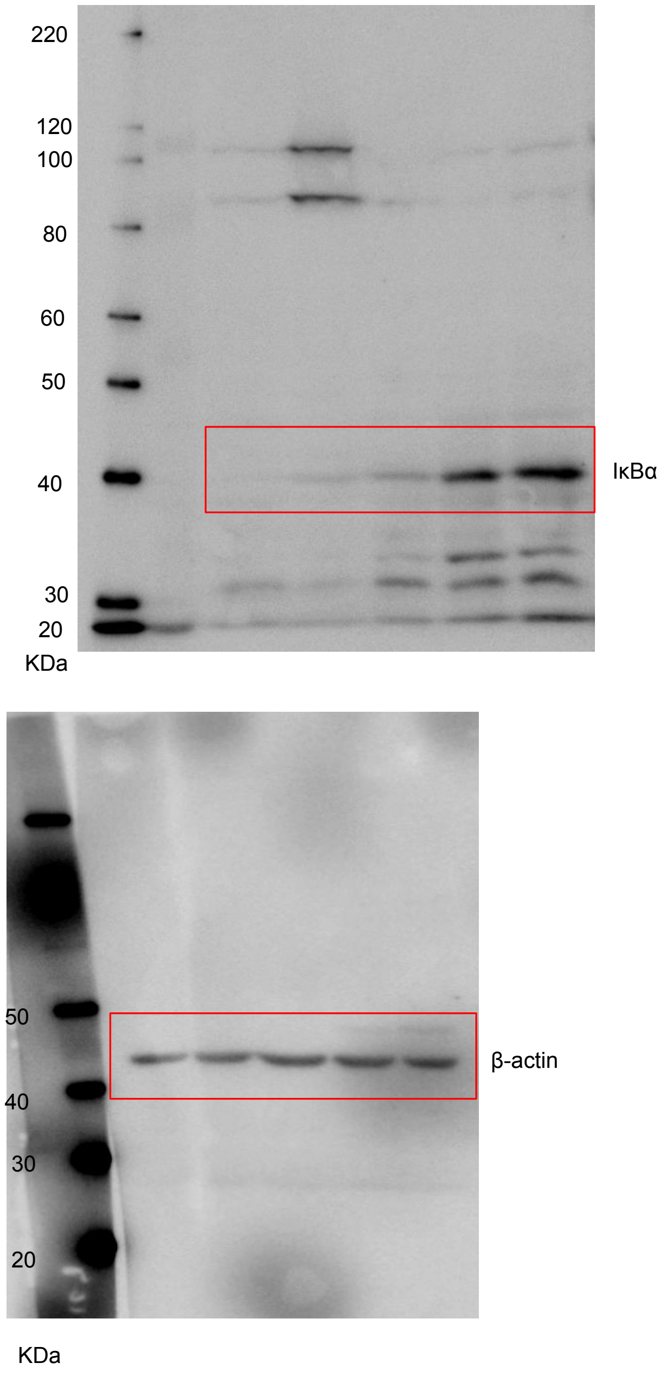

Fig.5C
/Jurkat

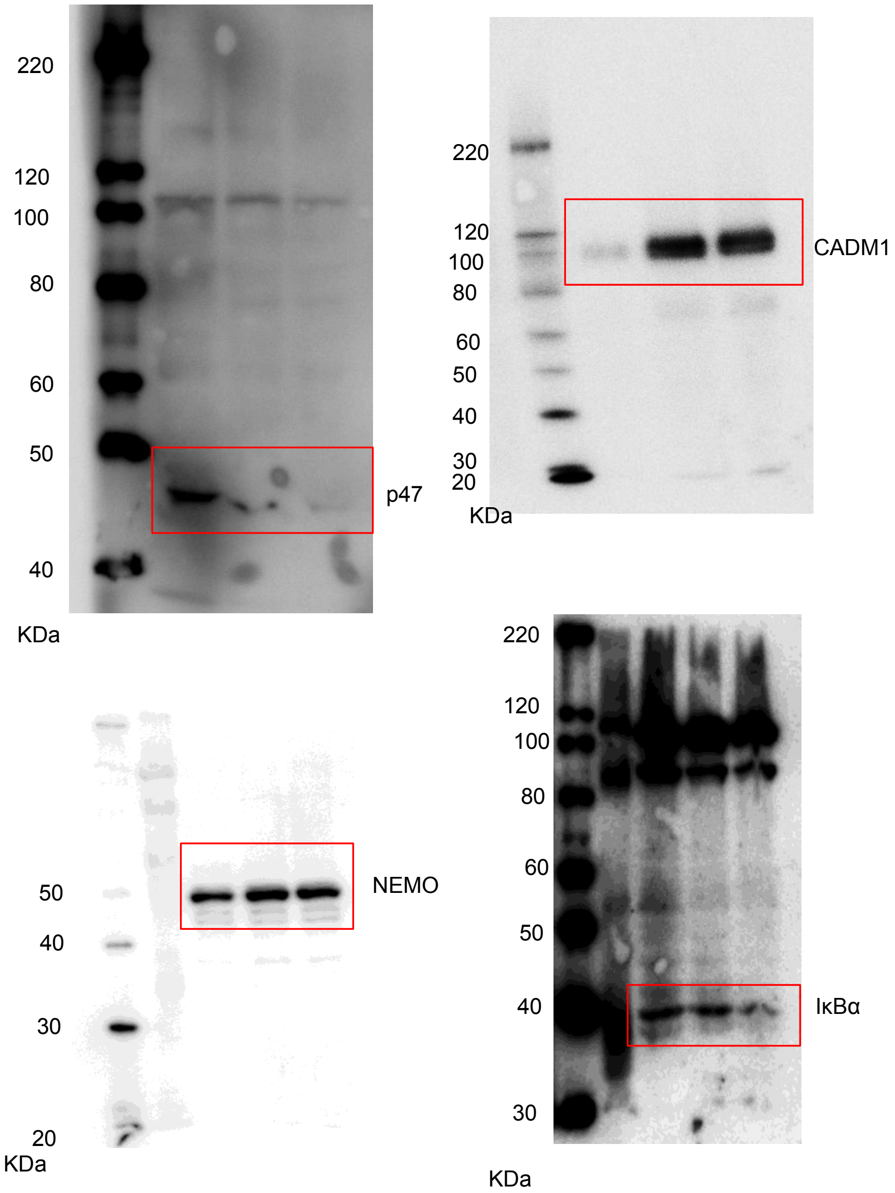

Fig.5C
/Jurkat

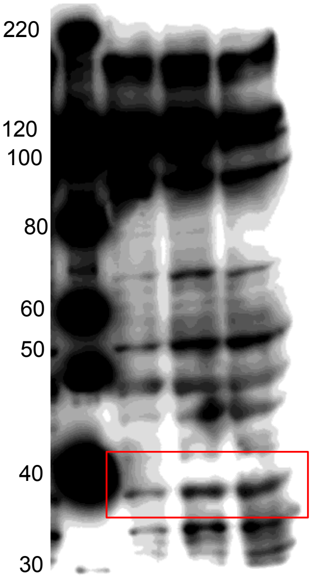

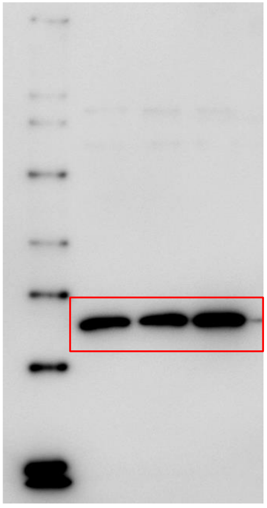

Fig.5C /MOLT4

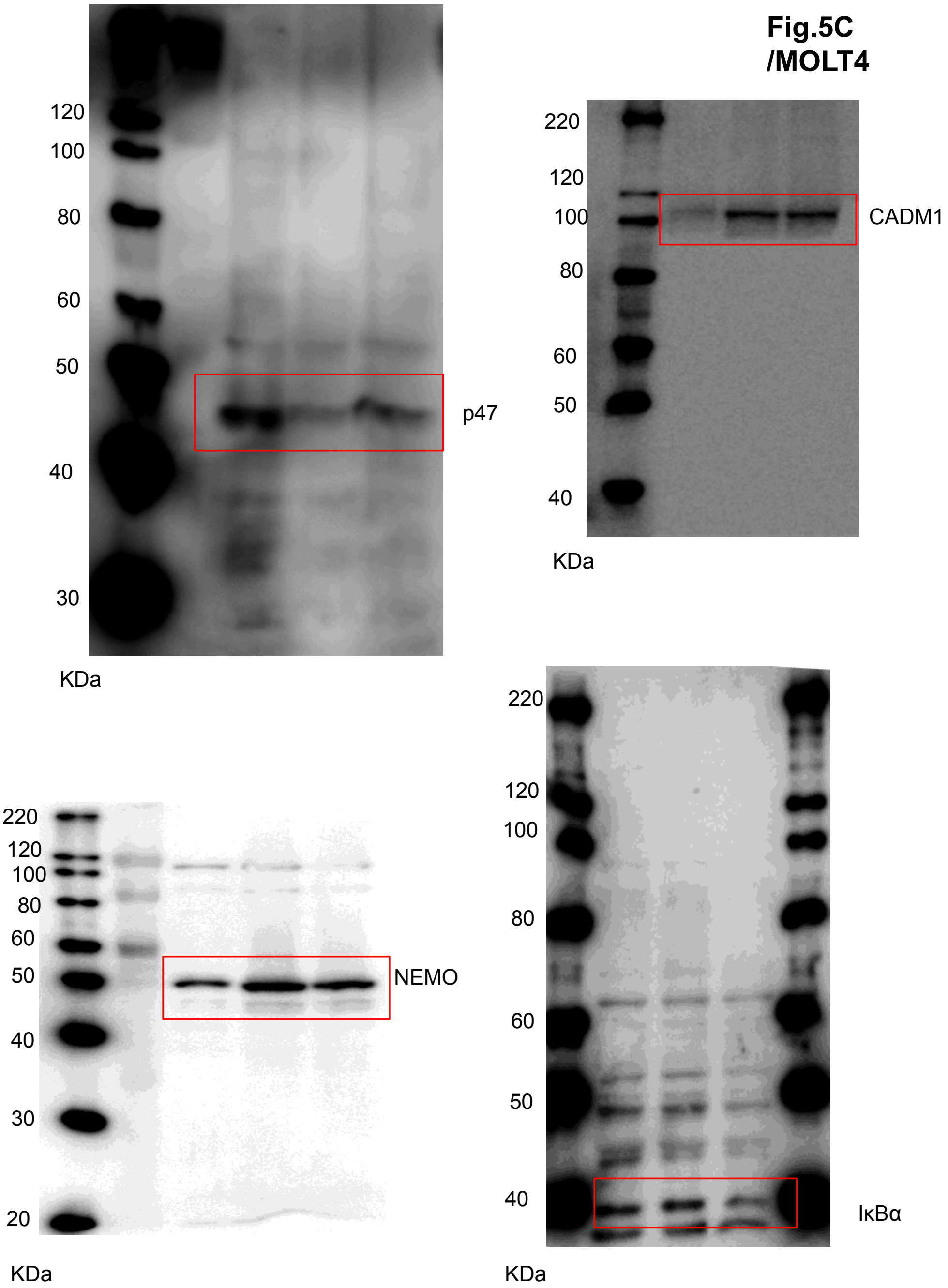

120
100
80
60
50
40
30
KDa
p47
220
120
100
80
60
50
40
KDa
CADM1
220
120
100
80
60
50
40
30
20
KDa
NEMO
220
120
100
80
60
50
40
KDa
IκBα

Fig.5C
/MOLT4

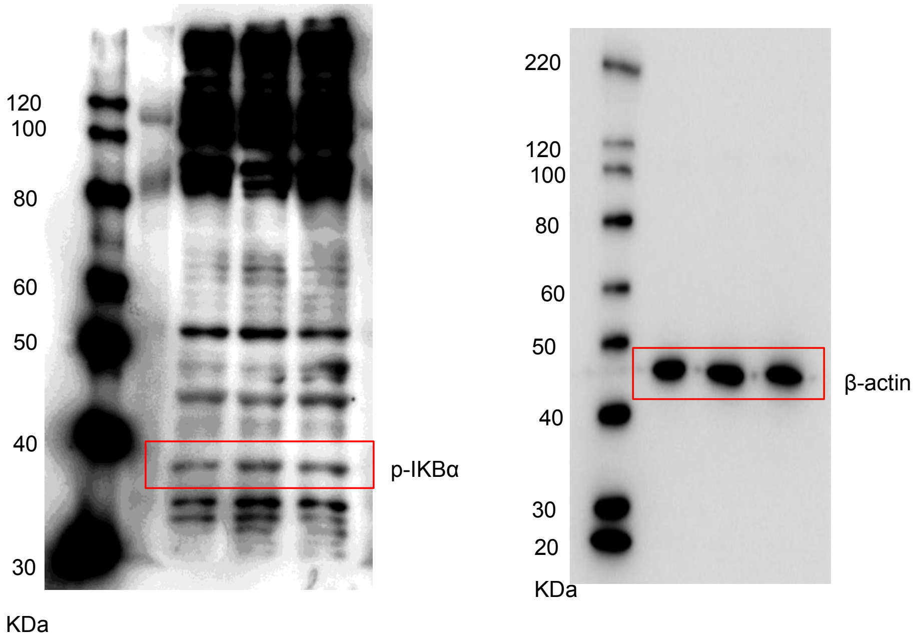
120
100
80
60
50
40
30
KDa
p-IKBα
220
120
100
80
60
50
40
30
20
KDa
β-actin

Fig.6A

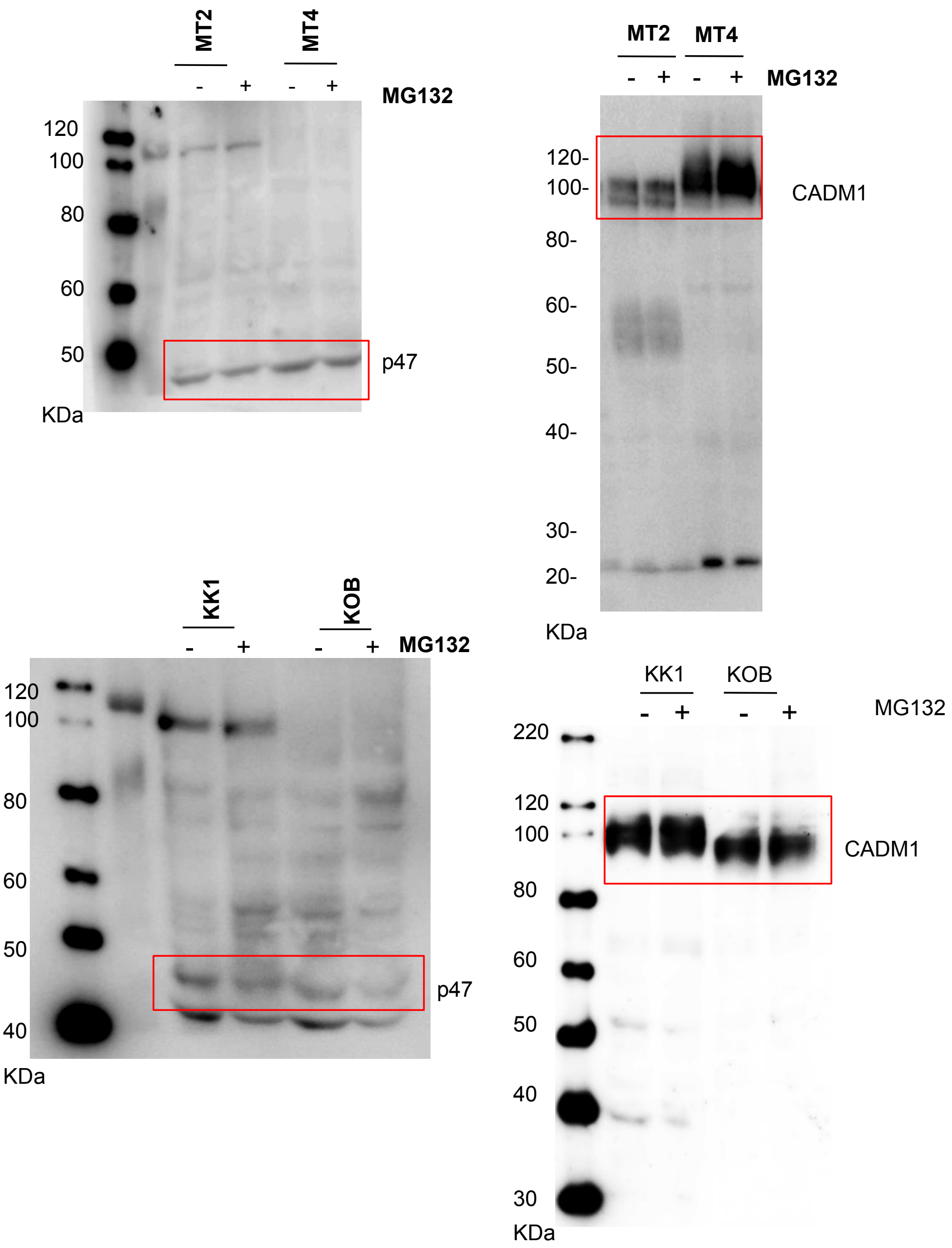
MT2
MT4
- + - +
MG132
120
100
80
60
50
KDa
p47
MT2
MT4
- + - +
MG132
120-
100-
80-
60-
50-
40-
30-
20-
KDa
CADM1
KK1
KOB
- + - +
MG132
120
100
80
60
50
40
KDa
p47
KK1
KOB
- + - +
MG132
220
120
100
80
60
50
40
30
KDa
CADM1

Fig.6A

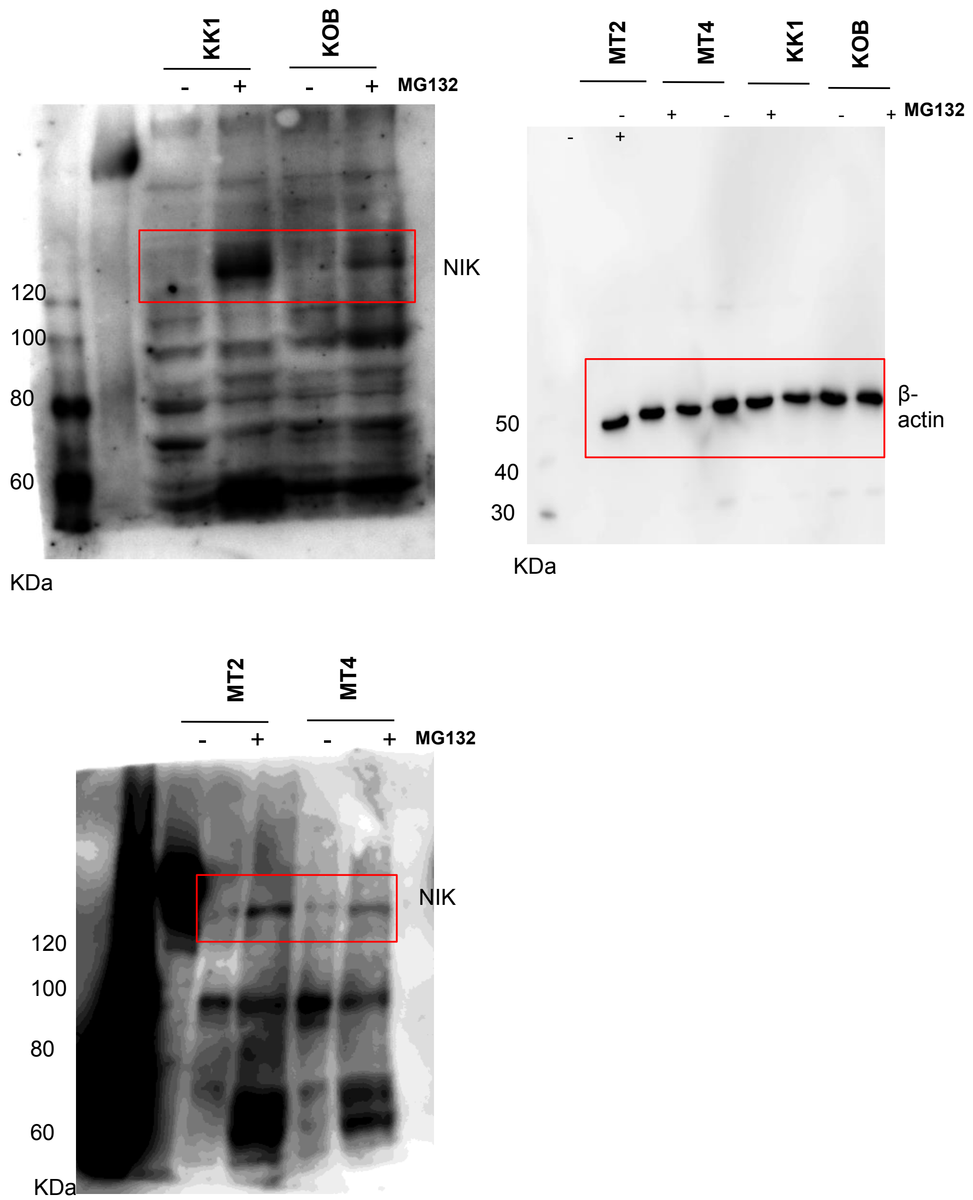
KK1
KOB
- + - + MG132
NIK
120
100
80
60
KDa
MT2
MT4
KK1
KOB
- + - + - + MG132
- +
β-
actin
50
40
30
KDa
MT2
MT4
- + - + MG132
NIK
120
100
80
60
KDa

Fig.6B

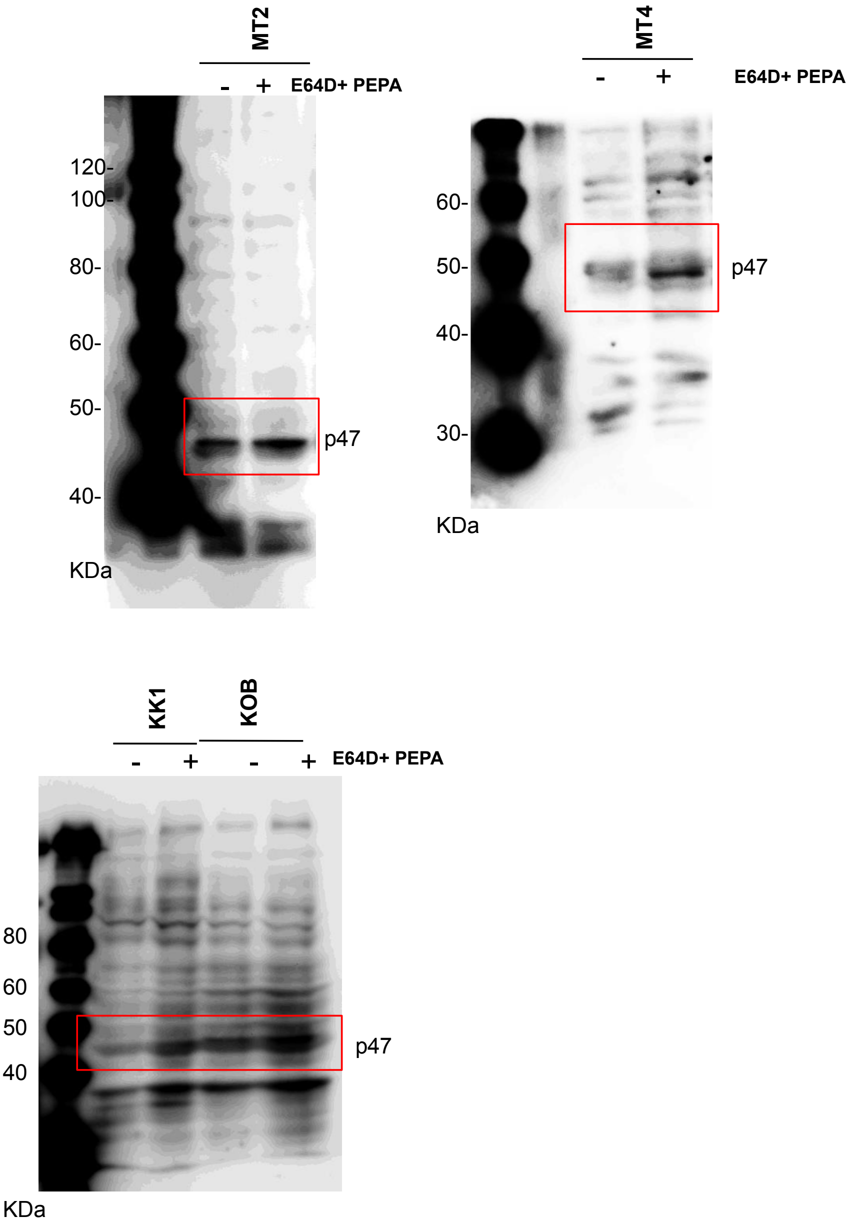
MT2
- + E64D+ PEPA
120-
100-
80-
60-
50-
p47
40-
KDa
MT4
- + E64D+ PEPA
60-
50-
p47
40-
30-
KDa
KK1
KOB
- + - + E64D+ PEPA
80
60
50
p47
40
KDa

Fig.6B

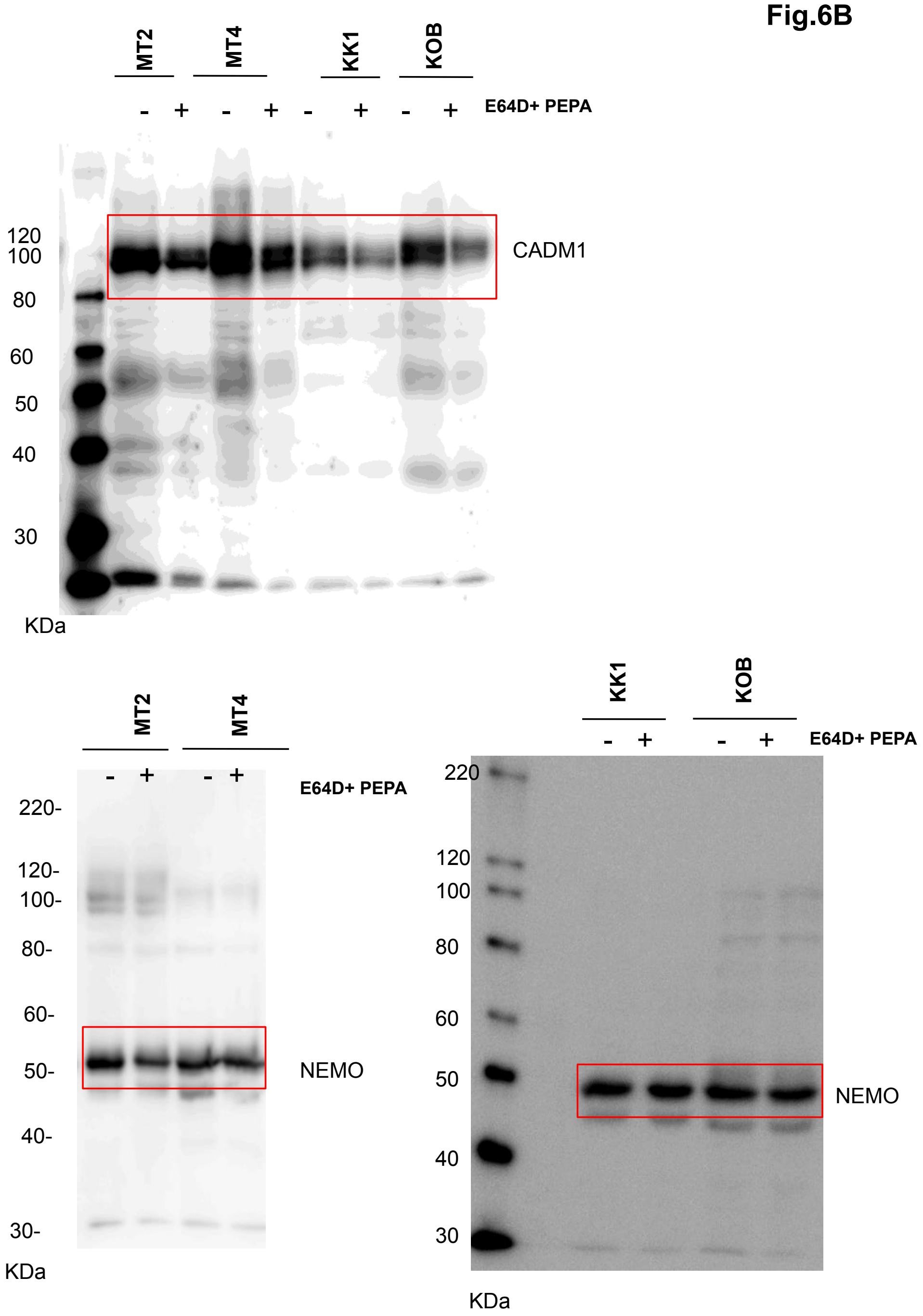
MT2
MT4
KK1
KOB
- + - + - + - +
E64D+ PEPA
120
100
80
60
50
40
30
KDa
CADM1
MT2
MT4
- + - +
E64D+ PEPA
220-
120-
100-
80-
60-
50-
40-
30-
KDa
NEMO
KK1
KOB
- + - +
E64D+ PEPA
220
120
100
80
60
50
40
30
KDa
NEMO

Fig.6B

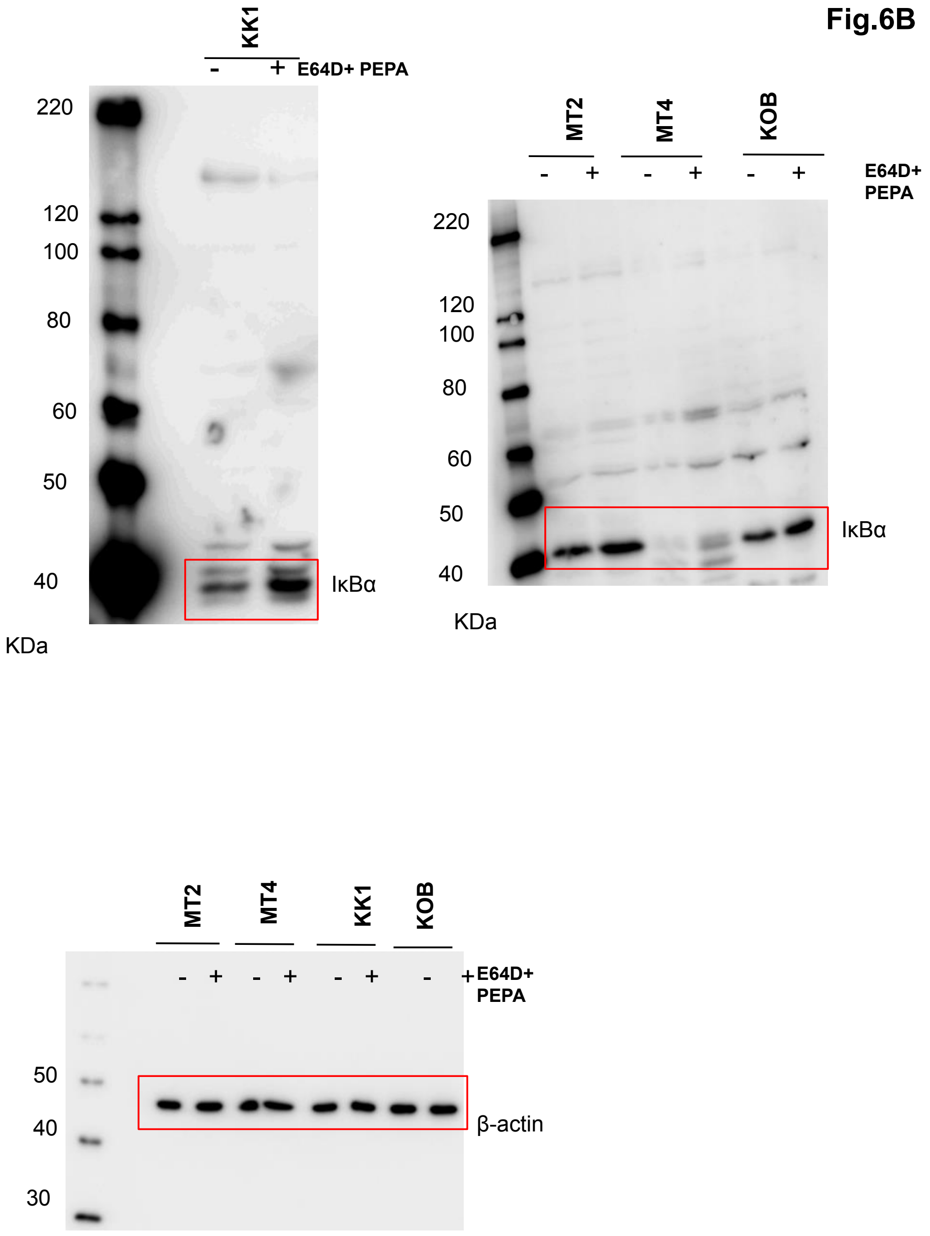

KK1
- + E64D+ PEPA
220
120
100
80
60
50
40
KDa
IκBα
MT2
MT4
KOB
- + - + - +
E64D+
PEPA
220
120
100
80
60
50
40
KDa
IκBα
MT2
MT4
KK1
KOB
- + - + - + - +
E64D+
PEPA
50
40
30
β-actin

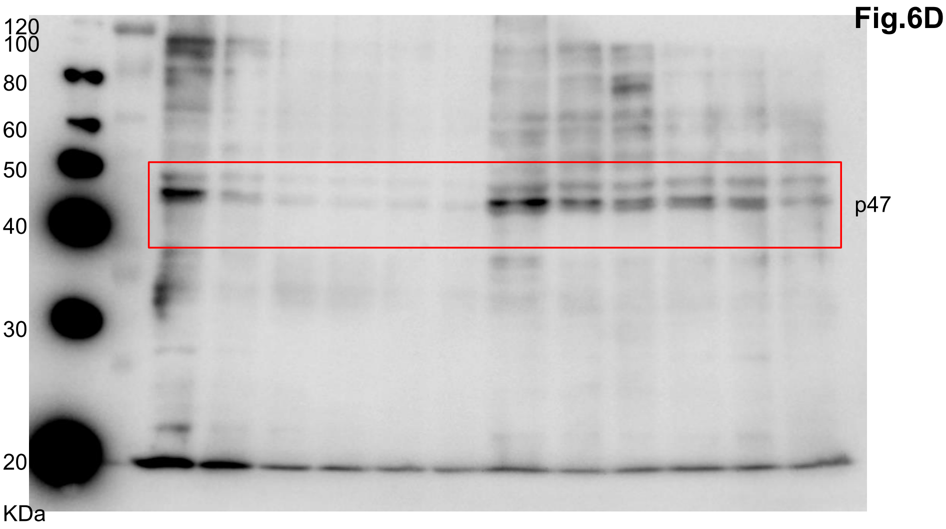
Fig.6D
120
100
80
60
50
40
30
20
KDa
p47

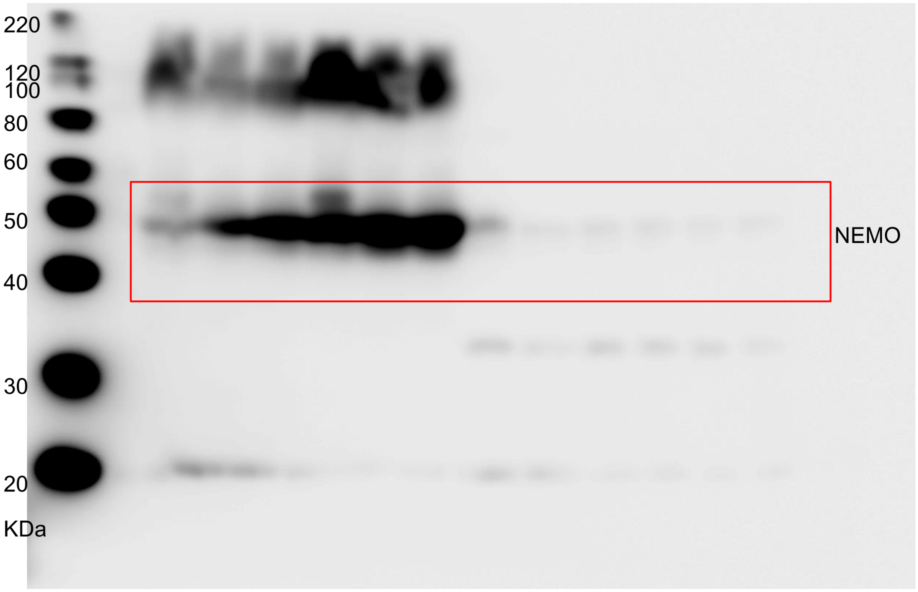
220
120
100
80
60
50
40
30
20
KDa
NEMO

Fig.6D

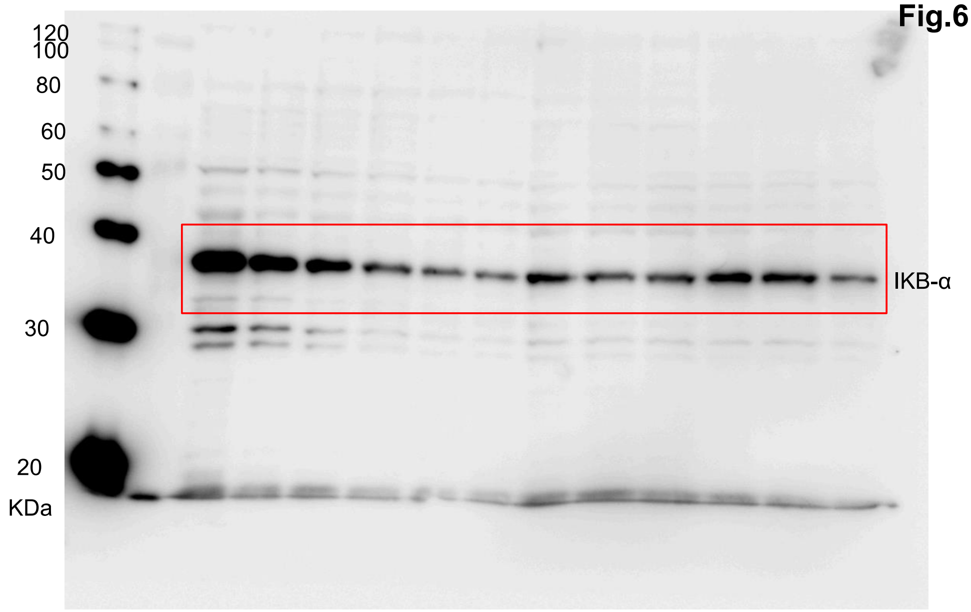

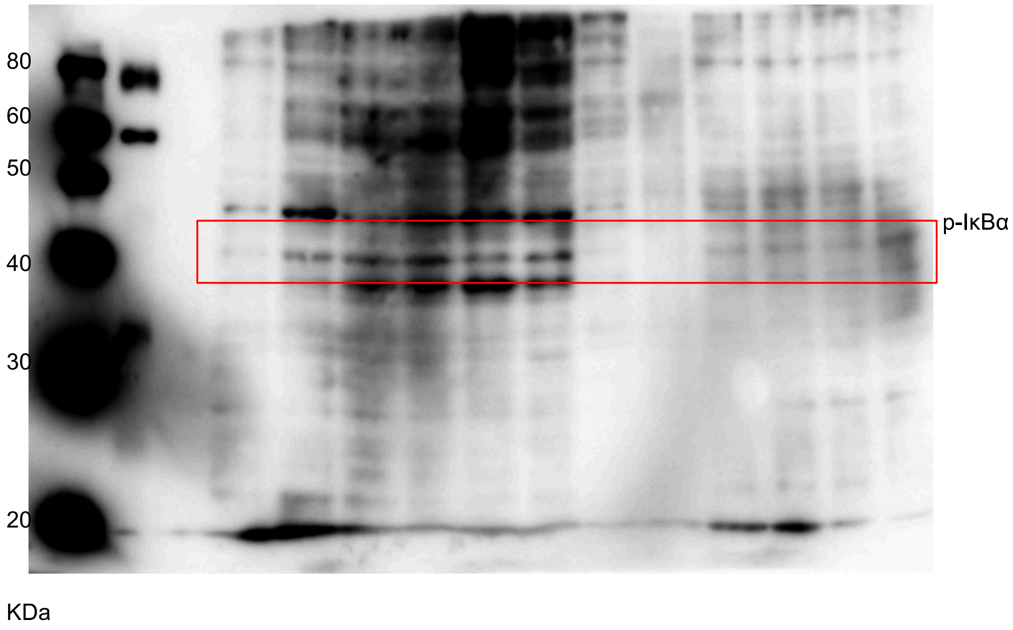

Fig.6D

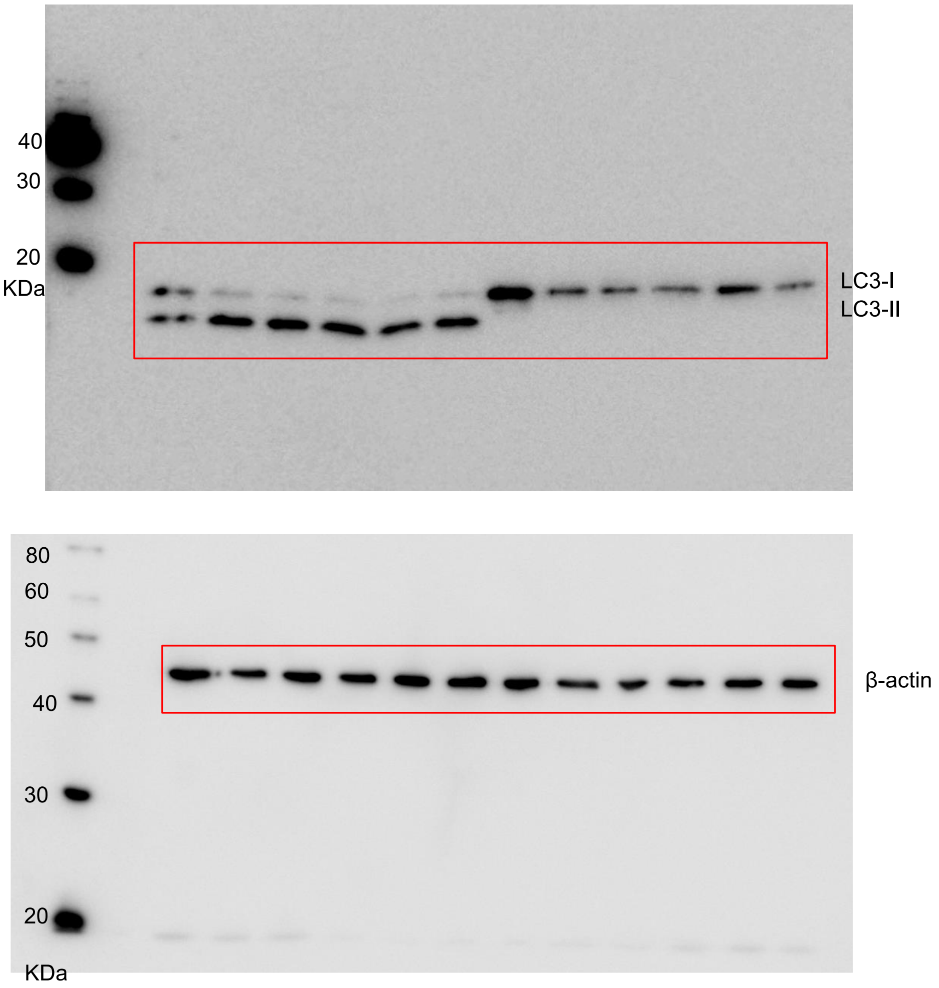

Fig.6E/ KOB

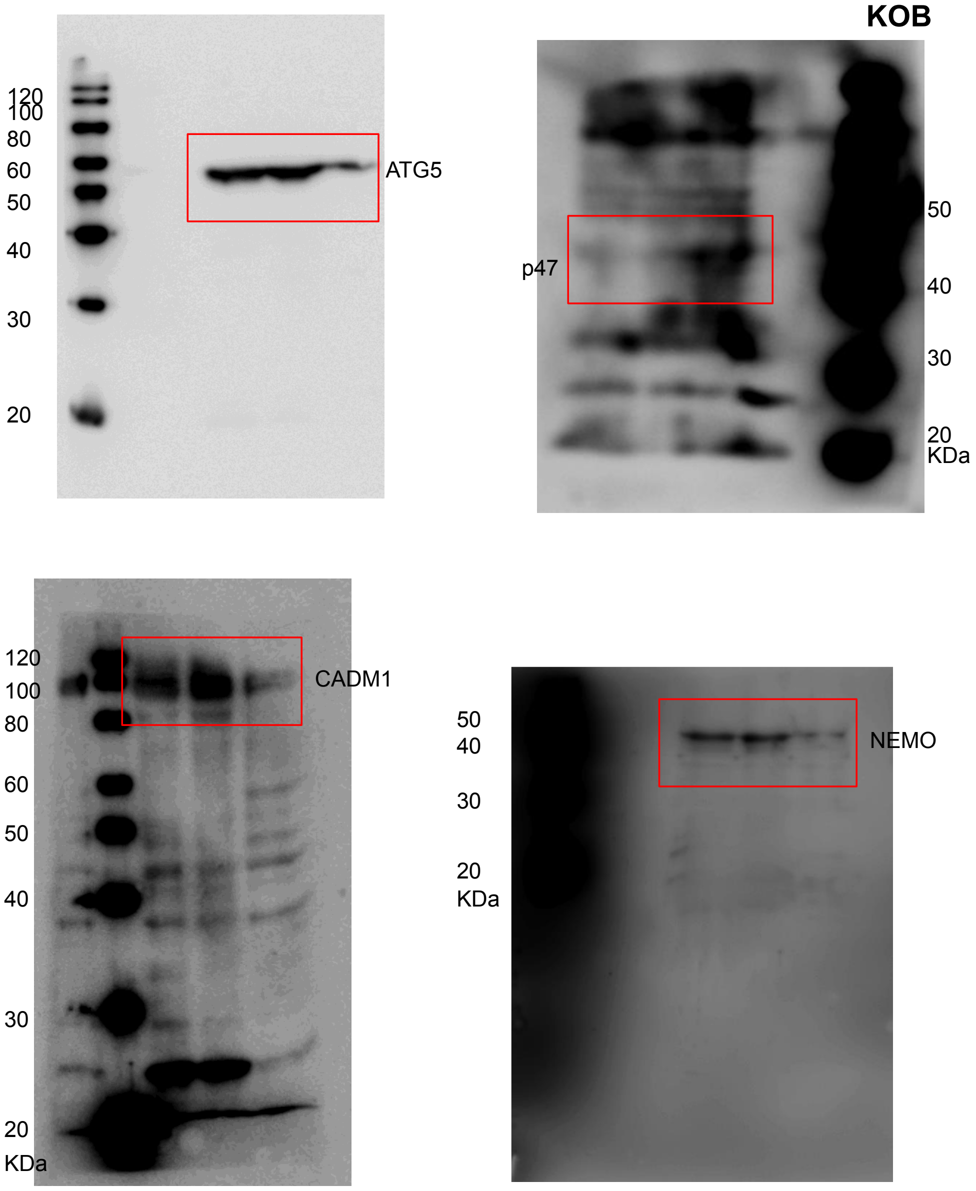

120
100
80
60
50
40
30
20
ATG5
p47
50
40
30
20
KDa
120
100
80
60
50
40
30
20
KDa
CADM1
50
40
30
20
KDa
NEMO

Fig.6E/ KOB

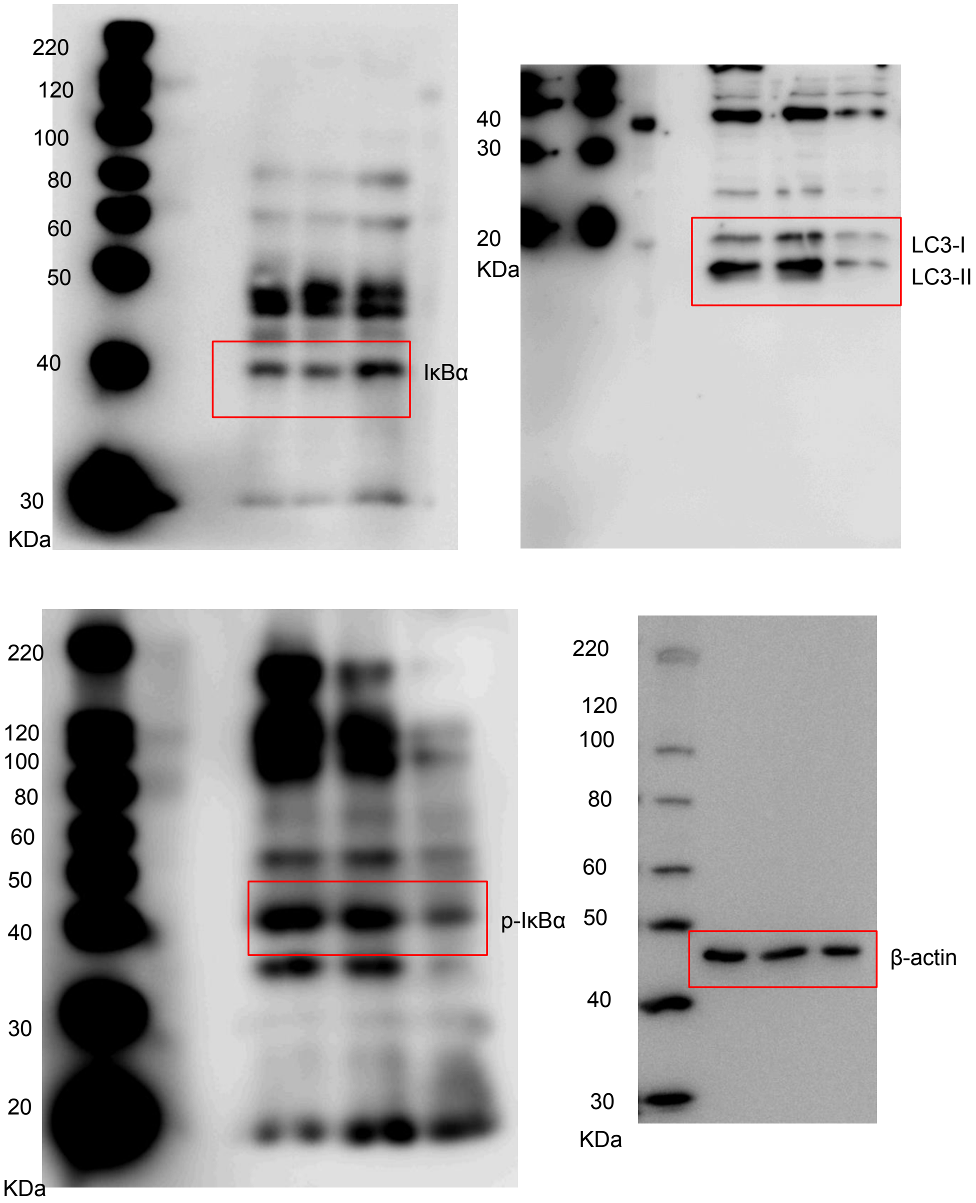
220
120
100
80
60
50
40
30
KDa
IκBα
40
30
20
KDa
LC3-I
LC3-II
220
120
100
80
60
50
40
30
20
KDa
p-IκBα
220
120
100
80
60
50
40
30
KDa
β-actin

Fig.6E/ KK1

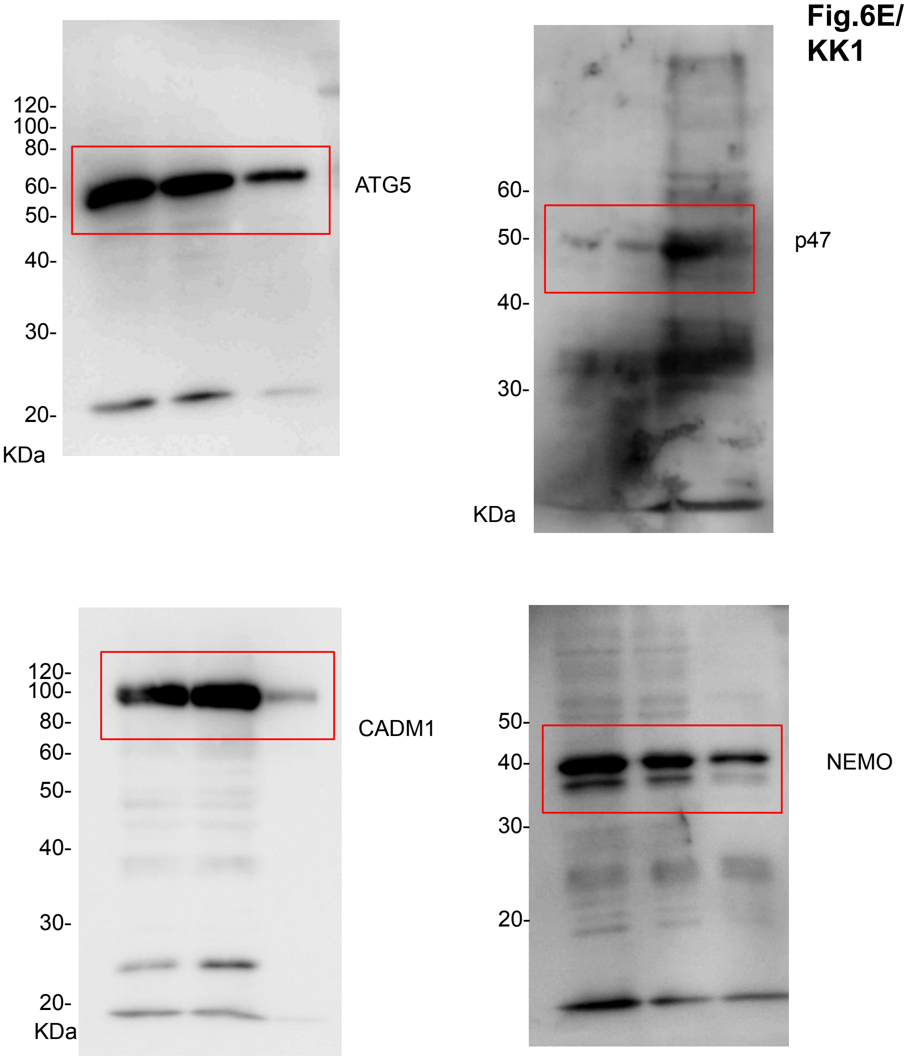
120-
100-
80-
60-
50-
40-
30-
20-
KDa
ATG5
60-
50-
40-
30-
KDa
p47
120-
100-
80-
60-
50-
40-
30-
20-
KDa
CADM1
50-
40-
30-
20-
NEMO

Fig.6E/ KK1

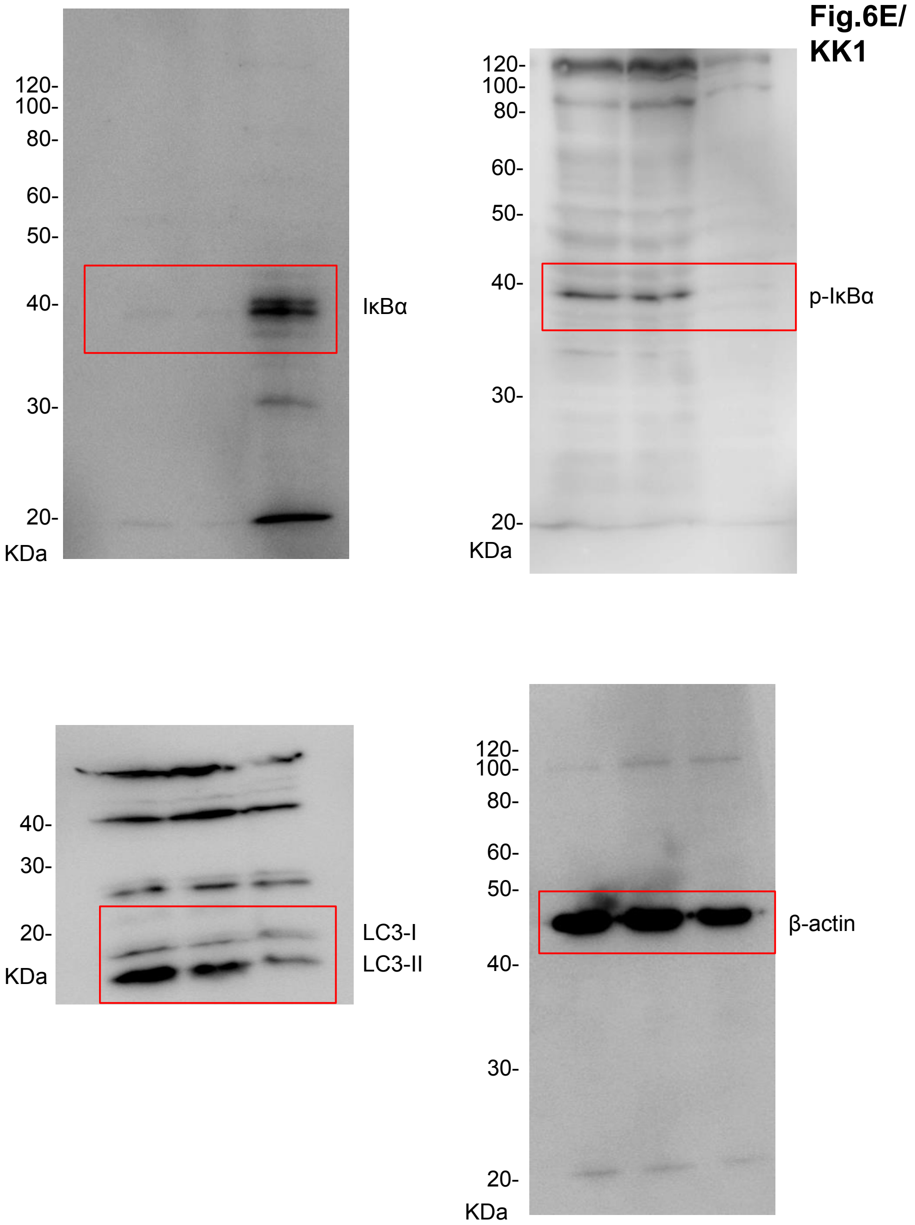

120-
100-
80-
60-
50-
40-
30-
20-
KDa
IκBα
120-
100-
80-
60-
50-
40-
30-
20-
KDa
p-IκBα
40-
30-
20-
KDa
LC3-I
LC3-II
120-
100-
80-
60-
50-
40-
30-
20-
KDa
β-actin

Fig.S4A
/KOB

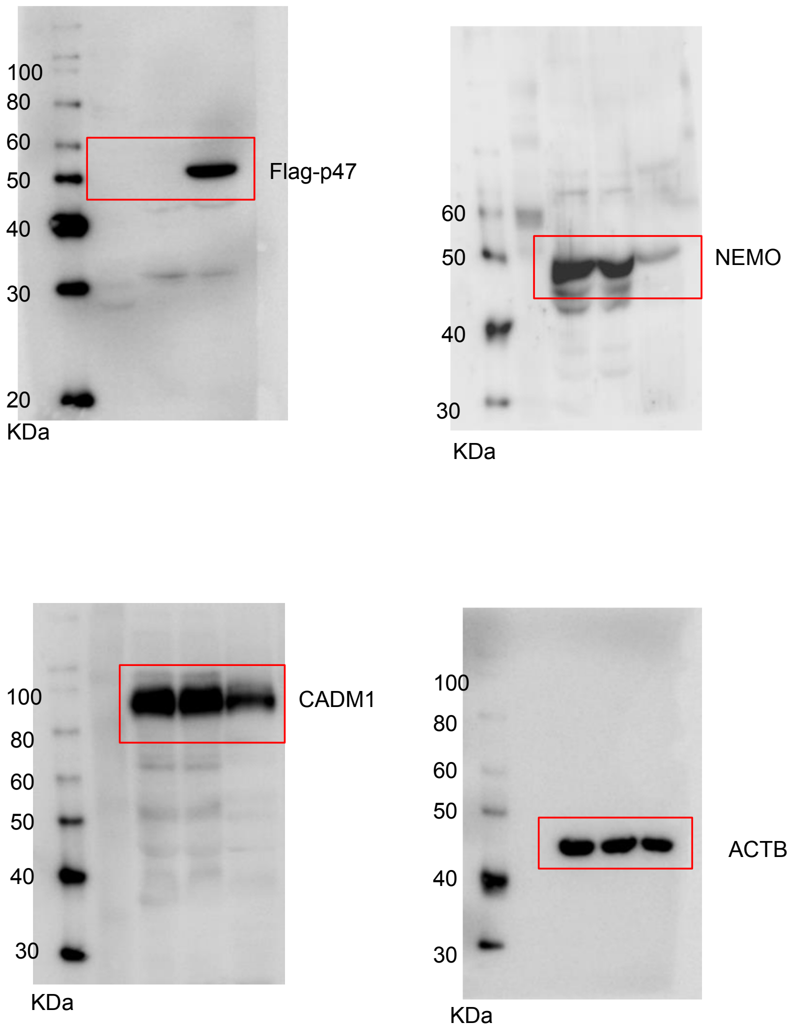
100
80
60
50
40
30
20
KDa
Flag-p47
60
50
40
30
KDa
NEMO
100
80
60
50
40
30
KDa
CADM1
100
80
60
50
40
30
KDa
ACTB

Fig.S4A
/S1T

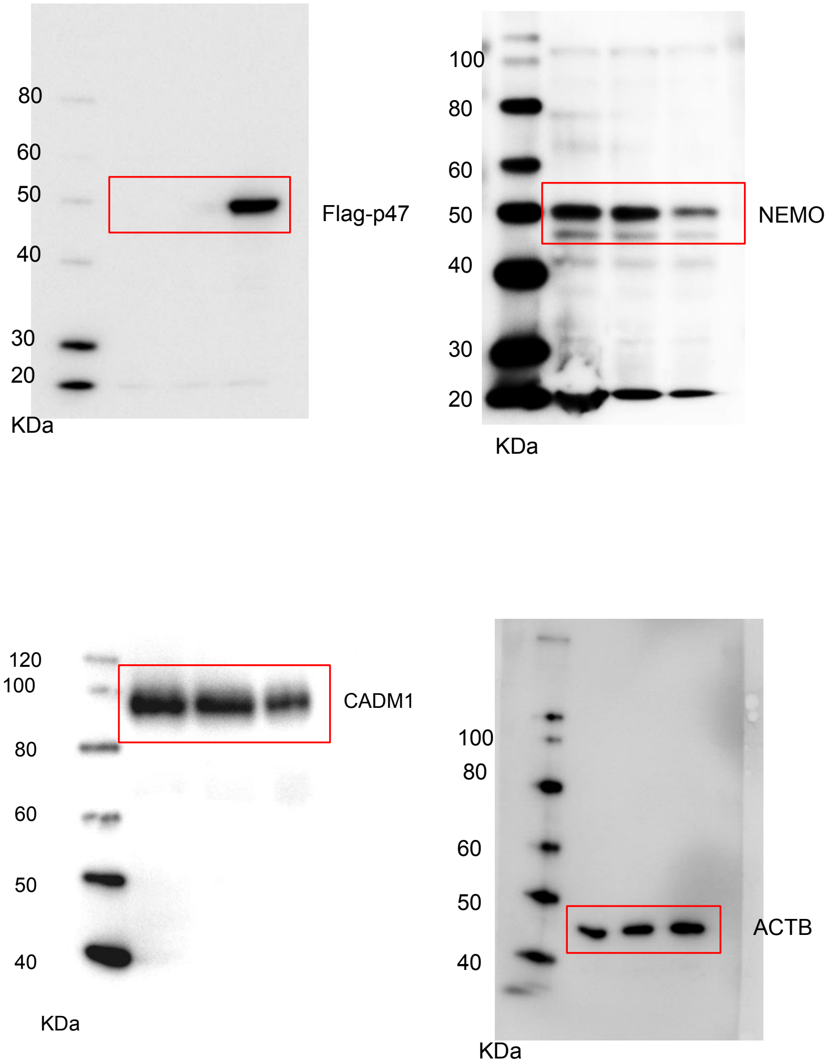
80
60
50
40
30
20
KDa
Flag-p47
100
80
60
50
40
30
20
KDa
NEMO
120
100
80
60
50
40
KDa
CADM1
100
80
60
50
40
KDa
ACTB

Fig. S5B

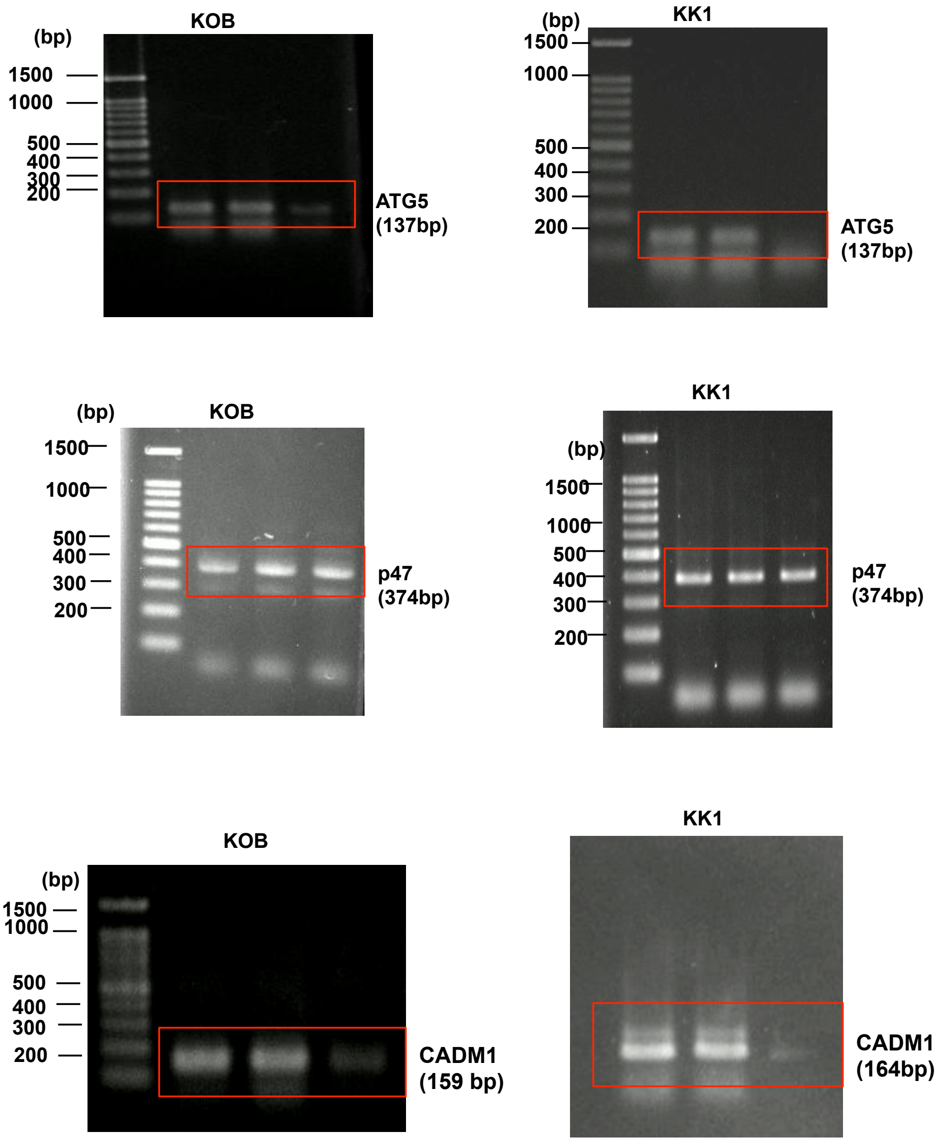
KOB
(bp)
1500
1000
500
400
300
200
ATG5
(137bp)
KK1
(bp)
1500
1000
500
400
300
200
ATG5
(137bp)
KOB
(bp)
1500
1000
500
400
300
200
p47
(374bp)
KK1
(bp)
1500
1000
500
400
300
200
p47
(374bp)
KOB
(bp)
1500
1000
500
400
300
200
CADM1
(159 bp)
KK1
CADM1
(164bp)

Fig. S5B

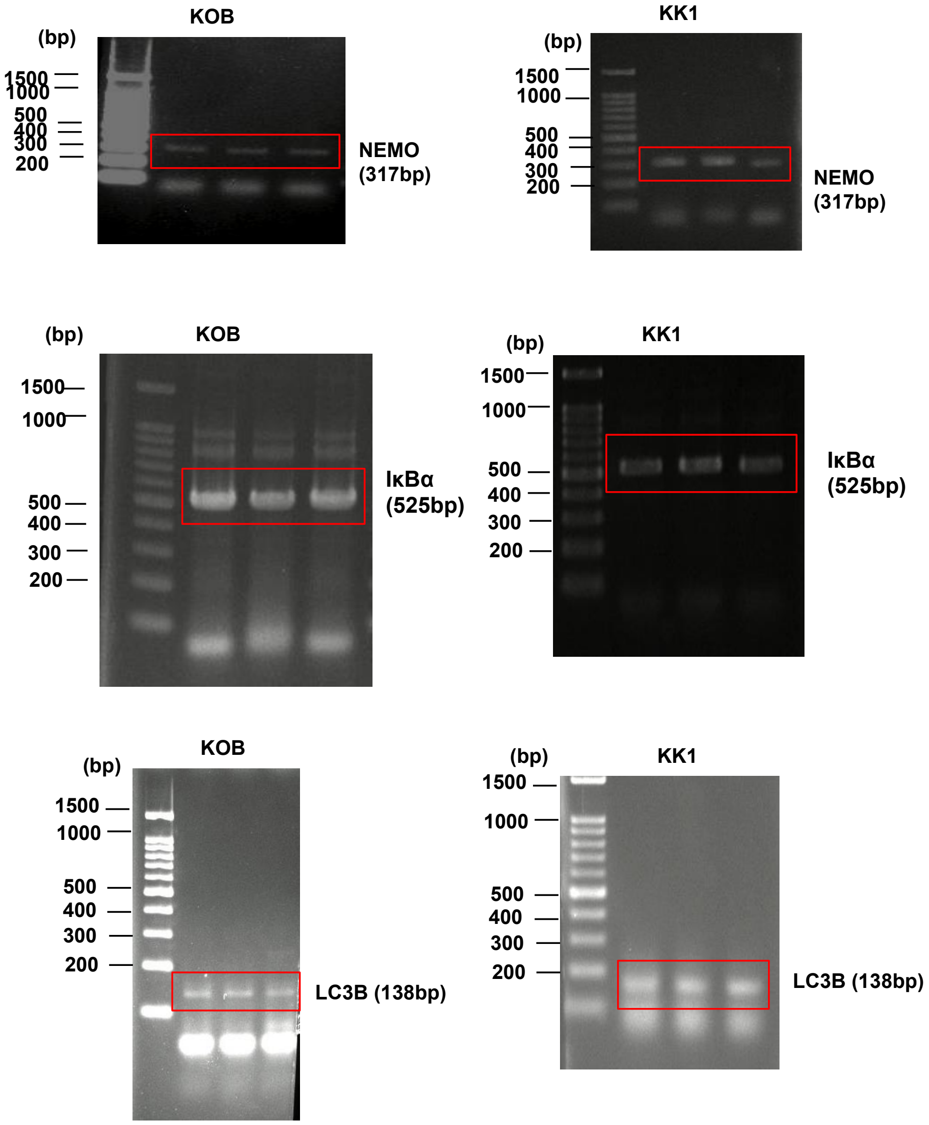
KOB
(bp)
1500
1000
500
400
300
200
NEMO
(317bp)
KK1
(bp)
1500
1000
500
400
300
200
NEMO
(317bp)
KOB
(bp)
1500
1000
500
400
300
200
IκBα
(525bp)
KK1
(bp)
1500
1000
500
400
300
200
IκBα
(525bp)
KOB
(bp)
1500
1000
500
400
300
200
LC3B (138bp)
KK1
(bp)
1500
1000
500
400
300
200
LC3B (138bp)

Fig. S5B

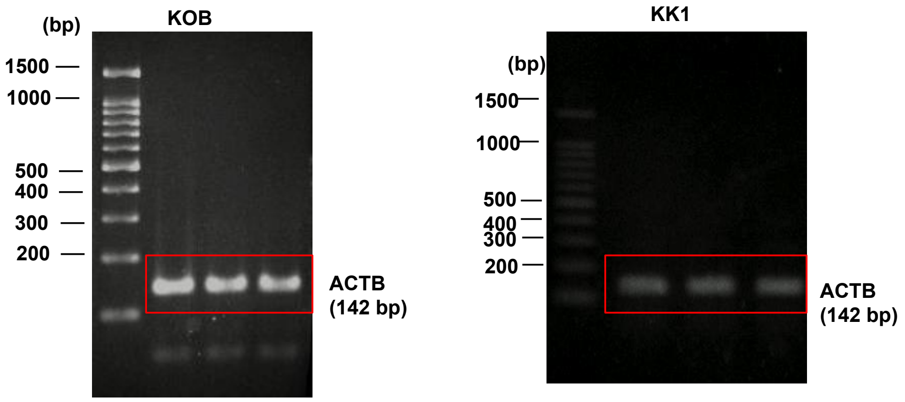
KOB
(bp)
1500
1000
500
400
300
200
ACTB
(142 bp)
KK1
(bp)
1500
1000
500
400
300
200
ACTB
(142 bp)

Supplementary References

1. Ichikawa, T., Nakahata, S., Fujii, M., Iha, H. & Morishita, K. Loss of NDRG2 enhanced activation of the NF-κB pathway by PTEN and NIK phosphorylation for ATL and other cancer development. *Sci Rep* **5,** 12841 (2015).
2. Nakahata S, Ichikawa T, *et al.* Loss of NDRG2 expression activates PI3K-AKT signalling via PTEN phosphorylation in ATLL and other cancers. *Nat Commun.* **5**, 3393 (2014).
